# GABA neurons in the sublaterodorsal tegmental nucleus suppress wakefulness in healthy and narcoleptic mice

**DOI:** 10.1371/journal.pbio.3003303

**Published:** 2026-07-08

**Authors:** HanHee Lee, Jimmy J. Fraigne, John H. Peever

**Affiliations:** Department of Cell & Systems Biology, University of Toronto, Toronto, Ontario, Canada; Institute of Science and Technology Austria, AUSTRIA

## Abstract

The sleep-wake cycle is generated by competing neural circuits that control the oscillation between wakefulness, rapid eye movement (REM) sleep, and non-REM (NREM) sleep. While the sublaterodorsal tegmental nucleus (SLD) is recognized for its role in REM sleep generation, the functional contribution of its GABAergic neurons (SLD^GABA^) to sleep-wake regulation remains poorly understood. Here, we found that SLD^GABA^ neurons function as a suppressor of wakefulness in both healthy (i.e., *orexin*^*+/+*^) and narcoleptic (i.e., *orexin*^*−/−*^) mice. In healthy mice, optogenetic silencing of SLD^GABA^ neurons rapidly induced robust wakefulness, while enhancing cortical and motor activity. Conversely, optogenetic activation of these neurons suppressed wakefulness and promoted NREM sleep. We found traces of SLD^GABA^ axonal projections to wake-promoting brain regions, providing an anatomical basis for their wake-suppressing effects. Importantly, we discovered that SLD^GABA^ neurons play a pathological role in narcolepsy: their activation in orexin-deficient narcoleptic mice triggered characteristic sleep attacks—rapid intrusions of NREM sleep during active wakefulness—while silencing these neurons rescued animals from both sleep attacks and cataplexy. Collectively, these findings establish SLD^GABA^ neurons as a key regulator of arousal state transitions and identify them as a novel therapeutic target for the treatment of narcolepsy.

## Abstract 2

The sublaterodorsal tegmental nucleus in the brainstem is a center for sleep control, but how the sleep-wake cycle is regulated is incompletely understood. This study shows that GABAergic neurons in this region suppress wakefulness, and that perturbing their activity level can regulate narcoleptic sleep attacks.

## Introduction

Current models propose that sleep-wake regulation is orchestrated by distinct neuronal populations and circuits that compete for each arousal state control (i.e., wakefulness, non-REM (NREM), and rapid eye movement (REM) sleep). The brainstem contains critical parts of this regulatory network, yet the precise mechanisms governing arousal state transitions remain incompletely understood [1–3]. Understanding these circuits is essential not only for basic sleep biology but also for developing treatments for sleep disorders.

The sublaterodorsal tegmental nucleus (SLD), located in the dorsal pons near key arousal centers, including the locus coeruleus and parabrachial nucleus, plays a strategic role within the sleep-wake regulatory network [4–6]. While the SLD is considered the generator of REM sleep, emerging evidence suggests it also influences wakefulness. Pharmacological studies demonstrate that SLD activation promotes REM sleep while suppressing wakefulness, whereas its inhibition prevents REM sleep and enhances wakefulness [7,8]. This control over both REM sleep and wakefulness positions the SLD as a critical hub in arousal state regulation

The SLD contains both glutamate and GABA neurons. Glutamate SLD (SLD^GLU^) neurons are well-established promoters of REM sleep and its associated muscle atonia [9–11]. In contrast, the functional role of GABA SLD (SLD^GABA^) neurons remains speculative. For example, some studies suggest that SLD^GABA^ neurons suppress wakefulness by inhibiting the ascending reticular activating systems [2,12,13], while other studies suggest that they regulate REM sleep [14] or have minimal effect on sleep-wake states [11]. These conflicting findings highlight a critical gap in our understanding of the function of SLD^GABA^ neurons in sleep-wake regulation, and underscore the need for precise, cell-type-specific approaches (i.e., genetic tagging and optogenetic) to resolve their role in sleep-wake control.

The clinical relevance of understanding SLD function extends to narcolepsy, a debilitating neurological disorder characterized by pathological intrusions of sleep into wakefulness. Narcolepsy results from the loss of orexin (also called hypocretin) signaling, which normally stabilizes wakefulness. Narcolepsy is characterized by pathological disruptions of wakefulness, manifesting as sleep attacks—sudden intrusion of NREM sleep into wakefulness—and cataplexy—intrusions of REM sleep muscle atonia into wakefulness [9,15]. Neuroanatomical and pharmacological evidence indicates that orexin neurons extensively innervate and interact with the SLD, and the loss of orexin signaling profoundly impacts SLD function [9,14,16–18]. Our recent work demonstrated that SLD^GLU^ neurons, which promote REM sleep muscle atonia in healthy mice (i.e., *orexin*^*+/+*^), drive cataplexy in narcoleptic mice (i.e., *orexin*^*−/−*^*)* [9,19]. This suggests that the SLD may be a central hub where orexin loss triggers the characteristic symptoms of narcolepsy.

Despite the clinical importance of the SLD in narcolepsy, the specific contribution of SLD^GABA^ neurons to the disorder’s pathophysiology remains unknown. Given their proposed role in suppressing wakefulness, we hypothesized that SLD^GABA^ neurons might contribute to the sleep attacks that affect narcoleptic patients. Understanding this relationship could reveal new therapeutic approaches for this disorder.

Here, we used optogenetics, electrophysiology, and genetically-assisted circuit mapping to establish the role of SLD^GABA^ neurons in sleep/wake control. First, our findings reveal that SLD^GABA^ neurons function as powerful suppressors of wakefulness, likely through suppressing wake-promoting brain regions. Then, we show that these neurons play a direct role in triggering narcoleptic sleep attacks, as their activation triggers pathological sleep intrusions while their silencing provides therapeutic rescue. Our findings not only resolve a longstanding question about the function of SLD^GABA^ neurons but also identify a potential intervention target to resolve the excessive sleepiness of narcolepsy.

## Results

### Silencing SLD^GABA^ neurons promotes wakefulness

**Fig 1:**
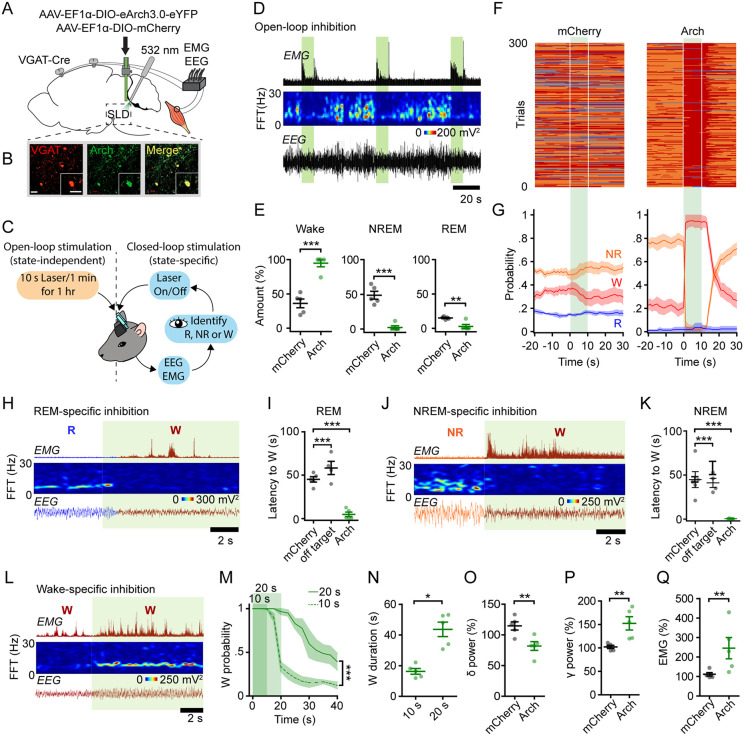
Silencing SLD^GABA^ neurons promotes wakefulness. **(A)** A schematic showing optogenetic silencing of SLD^GABA^ neurons coupled with EEG and EMG recordings. **(B)** Microscope images showing the expression of Arch (green) from *VGAT (*red) neurons in the SLD. **(C)** A schematic of open-loop (i.e., state-independent) and closed-loop (i.e., state-specific) stimulation paradigm. **(D)** Example polysomnogram recording with open-loop inhibition (i.e., 10 s laser stimulation every 60 s for 1 hour) of SLD^GABA^ neurons. Shown are EMG amplitude, EEG spectrogram, and EEG raw traces. **(E)** Mean percentages of wake, NREM, and REM sleep during 10 s open-loop inhibition of SLD^GABA^ neurons (mCherry *n* = 5 and Arch *n* = 5; unpaired *t* test). **(F)** Distribution of sleep-wake states in all laser trials aligned by the time of inhibition onset at t = 0 s (mCherry *n* = 5 and Arch *n* = 5). **(G)** Probability of wake, NREM, and REM sleep before, during, and after 10 s open-loop inhibition (mCherry *n* = 5 and Arch *n* = 5). **(H)** Example polysomnogram recording with REM-specific inhibition of SLD^GABA^ neurons. **(I)** Latency to wake from REM sleep upon inhibition of SLD^GABA^ neurons (mCherry *n* = 5, off-target *n* = 4, and Arch *n* = 5, one-way ANOVA with Tukey’s Multiple Comparison test). **(J)** Example polysomnogram recording with NREM-specific inhibition of SLD^GABA^ neurons. **(K)** Latency to wake from NREM sleep upon inhibition of SLD^GABA^ neurons (mCherry *n* = 5, off-target *n* = 4, and Arch *n* = 5, one-way ANOVA with Tukey’s Multiple Comparison test). **(L)** Example polysomnogram recording with wake-specific inhibition of SLD^GABA^ neurons. **(M)** Probability of wakefulness in response to 10 and 20 s inhibition of SLD^GABA^ neurons (*n* = 5, two-way ANOVA with Bonferroni post-test). **(N)** Duration of wakefulness in response to 10 and 20 s inhibition of SLD^GABA^ neurons (*n* = 5, paired *t* test). **(O** and **P)** Mean change in δ and γ EEG power during wake-specific inhibition (mCherry *n* = 5, Arch *n* = 5, unpaired *t* test). **(Q)** Mean change in EMG activity during wake-specific inhibition (mCherry *n* = 5 and Arch *n* = 5; unpaired *t* test). Brain wave bands: δ (delta, 0.5-4 Hz) and γ (gamma, 30-100 Hz). Green patches indicate time of laser-ON period. All error bars and shades represent ±s.e.m. * p < 0.05, ** p < 0.01, *** p < 0.001 indicate significant differences. Scale bar 25 μm. The data underlying this Figure can be found in S1 Data.

To test the role of SLD^GABA^ neurons in sleep-wake regulation, we used optogenetics to silence their activity across natural sleep-wake states in mice. We did this by bilaterally injecting either AAV-EF1a-DIO-eArch3.0-eYFP or AAV-EF1a-DIO-mCherry (control) into the SLD of male *VGAT-Cre* mice (Fig 1A and 1B). Then, we implanted optic fibers above either Arch- or mCherry-expressing SLD^GABA^ neurons for light (532 nm) delivery (S1A and S1B Fig). EEG and EMG electrodes were implanted to monitor and identify each arousal state.

We first silenced the activity of SLD^GABA^ neurons using an open-loop protocol (i.e., state-independent; 10 s continuous silencing every minute for one hour), then quantified the changes in arousal states (Fig 1C) [20]. We found that silencing the SLD^GABA^ neurons reliably induced episodes of wakefulness (Fig 1D). To quantify this effect, we aligned the 10 s inhibition window from all mice by the time of its onset and then calculated the amount and probability of each arousal state across time (Fig 1F). We found that silencing of SLD^GABA^ neurons induced a robust increase in the amount and probability of wakefulness and a complementary decrease in both NREM and REM sleep (Fig 1E–1G). The same light delivery to the mCherry-expressing SLD^GABA^ neurons (control) had no effect on the arousal states (Figs 1E–1G and S2) nor did light applied to regions adjacent to Arch-expressing SLD^GABA^ neurons (i.e., off-target optic fibers) (Fig 1I and 1K). This finding supports that silencing of SLD^GABA^ neurons is inducing wakefulness rather than the light itself.

To further determine the role of SLD^GABA^ neurons, we applied a closed-loop protocol (i.e., state-specific) whereby we selectively silenced SLD^GABA^ neurons during each arousal state. To do this, we monitored real-time EEG/EMG activity and silenced these neurons as soon as spontaneous episodes of either wakefulness, NREM, or REM sleep occurred (Fig 1C). We found that silencing SLD^GABA^ neurons either during NREM or REM sleep induced instantaneous transitions into alert and motorically engaged wakefulness (Figs 1H–1K and S1C–S1F and S1 Video). We also found that silencing SLD^GABA^ neurons during wakefulness sustained wakefulness. A prolonged silencing of SLD^GABA^ neurons (20 s versus 10 s) induced a longer-lasting wakefulness and maintained a higher probability of wakefulness over time (Fig 1L–1N). These findings demonstrate that silencing SLD^GABA^ neurons not only initiates wakefulness from sleep but also sustains wakefulness.

Next, we investigated whether silencing SLD^GABA^ neurons not only triggers wakefulness but also can enhance the features of wakefulness (i.e., increase brain and muscle activity). To do this, we silenced SLD^GABA^ neurons while the animals were already awake. We found that silencing SLD^GABA^ neurons during wakefulness increased the power of wake-related cortical activity (i.e., gamma, 30–100 Hz; Fig 1P) while simultaneously decreasing sleep-related cortical activity (i.e., delta, 0.5–4 Hz; Figs 1O and S1H–S1L). In addition, we found that silencing SLD^GABA^ neurons during wakefulness also enhanced muscle activity (i.e., EMG) (Figs 1Q and S1G). Consistent with these physiological changes, silencing SLD^GABA^ neurons during wakefulness was frequently accompanied by active wake behaviors, most prominently locomotion (eArch3.0:eYFP; 43.8% ± 19.6%:16.91 ± 3.37%; S1M Fig). Together, our findings show that silencing SLD^GABA^ neurons not only triggers wakefulness but also enhances brain and muscle activity that characterizes active arousal. These findings suggest that SLD^GABA^ neurons play a powerful role in suppressing wakefulness and its wake-related features.

### Activation of SLD^GABA^ neurons suppresses wakefulness

**Fig 2:**
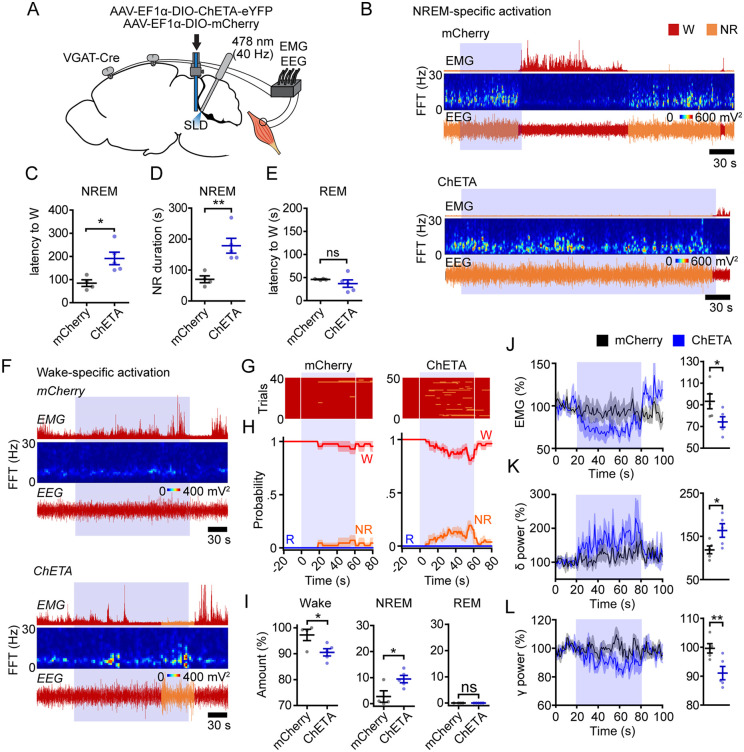
Activating SLD^GABA^ neurons suppresses wakefulness. **(A)** A schematic showing optogenetic activation (478 nm, 40 Hz, 5 ms pulses) of SLD^GABA^ neurons coupled with EEG and EMG recordings. **(B)** Example polysomnograms with NREM-specific activation of SLD^GABA^ neurons (top: mCherry and bottom: ChETA). Shown are EMG amplitude, EEG spectrogram, and EEG raw traces. **(C)** Latency to wake from NREM sleep upon activation of SLD^GABA^ neurons (mCherry *n* = 4 and ChETA *n* = 5; unpaired *t* test). **(D)** Duration of NREM sleep upon activation of SLD^GABA^ neurons (mCherry *n* = 4 and ChETA *n* = 5; unpaired t-tests). **(E)** Latency to wake from REM sleep upon activation of SLD^GABA^ neurons (mCherry *n* = 4 and ChETA *n* = 5, unpaired *t* test). **(F)** Example polysomnograms for wake-specific activation (60 s) of SLD^GABA^ neurons (top: mCherry and bottom: ChETA). **(G)** Distribution of sleep-wake states in all wake-specific activation trials (60 s) aligned by the time of activation onset t = 0 s (mCherry *n* = 4 and ChETA *n* = 5). **(H)** Probability of wake, NREM, and REM sleep before, during, and after the 60 s wake-specific activation (mCherry *n* = 4 and ChETA *n* = 5**)**. **(I)** Mean percentages of wake, NREM, and REM sleep during the 60 s wake-specific activation (mCherry *n* = 4 and ChETA *n* = 5; unpaired *t* test) **(J) *LEFT***: EMG activity before, during, and after 60 s wake-specific activation (mCherry *n* = 5 and ChETA *n* = 5). ***RIGHT***: Mean EMG activity during the 60 s wake-specific activation (mCherry *n* = 5 and ChETA n = 5; unpaired *t* test). **(K** and **L) *LEFT*:** δ and γ EEG activity before, d*n*uring, and after 60 s wake-specific activation (mCherry *n* = 5 and ChETA *n* = 5). ***RIGHT*:** Mean δ and γ EEG activity during the 60 s wake-specific activation (mCherry *n* = 5 and ChETA *n* = 5; unpaired *t* test). EEG bands: δ (delta, 0.5-4 Hz) and γ (gamma, 30-100 Hz). Blue patches indicate time of laser-ON period. All error bars and shades represent ±s.e.m. * p < 0.05, ** p < 0.01, *** p < 0.001 indicate significant differences. The data underlying this Figure can be found in S2 Data.

To further examine the role of SLD^GABA^ neurons in sleep-wake control, we optically activated these neurons and assessed their impact on each arousal state (Fig 2). To do this, we bilaterally injected AAV-EF1a-DIO-ChETA-eYFP or AAV-EF1a-DIO-mCherry (control) into the SLD of male *VGAT-Cre* mice (Fig 2A). Optic fibers were positioned above SLD^GABA^ neurons and EEG/EMG readout was used to assess how the activation of SLD^GABA^ neurons affects arousal states. Light (478 nm) was delivered in pulses (5 ms) at 40 Hz to match the natural firing rates of SLD^GABA^ neurons reported in the literature [21–23].

Because silencing SLD^GABA^ neurons promotes wakefulness (Fig 1), we predicted that their activation would suppress wakefulness and induce sleep. Using a closed-loop protocol, we found that the activation of SLD^GABA^ neurons during NREM sleep significantly lengthened NREM sleep by preventing the entrance into wakefulness (Fig 2B–2D). In contrast, we found that the activation of SLD^GABA^ neurons during REM sleep did not prolong REM sleep (Fig 2E). We also found that neither cortical nor motor activity was affected by the activation during NREM or REM sleep (S3B–S3E Fig), indicating that the effect of the activation specifically prolongs the state of NREM sleep. Light (478 nm, 40 Hz) delivery to mCherry-expressing SLD^GABA^ neurons had no effect during NREM or REM sleep (S2 Fig).

Next, we found that the activation of SLD^GABA^ neurons suppressed the probability and amount of wakefulness by increasing NREM sleep but not REM sleep (Fig 2F–2I). In addition, we found that activating SLD^GABA^ neurons reduced the cortical and motor indices of wakefulness. Specifically, we found that activation of SLD^GABA^ neurons during wakefulness significantly increased and decreased EEG δ and γ powers, respectively, while simultaneously decreasing levels of muscle activity (i.e., EMG) (Figs 2J–2L and S3F–S3K). Together, these data show that the SLD^GABA^ neurons suppress wakefulness and the cortical and motor features that support it.

### SLD^GABA^ axonal projections are found in wake-promoting brain areas

Having shown that SLD^GABA^ neurons suppress wakefulness, we next wanted to explore their projection patterns. To do this, we injected a Cre-dependent anterograde viral tracer (AAV-EF1a-DIO-eArch3.0-eYFP) into the SLD of *VGAT-Cre* mice, then identified brain areas with traces of eYFP-expressing axonal projections.

**Fig 3:**
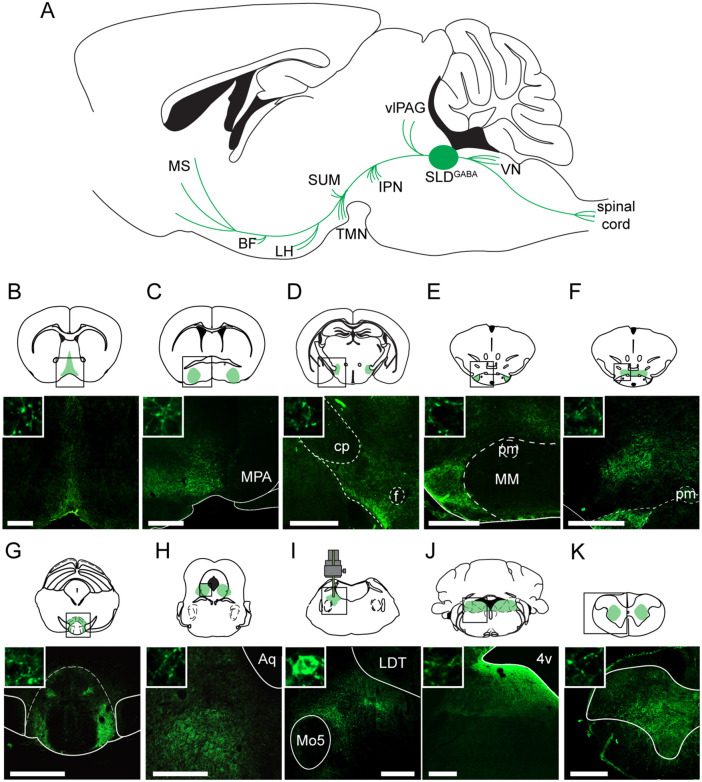
SLD^GABA^ axonal projections are found in wake-promoting nuclei. **(A)** A sagittal schematic of the brain illustrating the distribution of SLD^GABA^ axonal projections. **(B–K)** Schematics (***TOP***) and microscope images (***BOTTOM***) showing coronal section of each brain nuclei that express SLD^GABA^ axonal projections (green fluorescence). The smaller microscope images inserted on the top left are magnified images of the neural projections (25 μm x 25 μm in dimension). These brain nuclei are B) MS, C) BF, D) LH, E) TMN, F) SUM, G) IPN, H) vlPAG, I) SLD (injection site), J) VN, and K) spinal cord. Abbreviation: medial septum (MS), basal forebrain (BF), medial preoptic area (MPA), lateral hypothalamus (LH), cerebral peduncle (cp), fornix (f), tuberomammillary nucleus (TMN), principle mamillary tract (pm), medial mammillary body (MM), supramammilary nucleus (SuM), interpeduncular nucleus (IPN), ventrolateral periaqueductal gray (vlPAG), sublaterodorsal tegmental nucleus (SLD), aqueduct (Aq), laterodorsal tegmental nucleus (LDT), trigeminal motor nucleus (Mo5), vestibular nucleus (VN) and 4th ventricle (4V). Scale bar 400 μm. Sagittal and Coronal brain schematics are from Allen Brain Atlas 3rd edition.

We found traces of SLD^GABA^ axonal projections in brain regions previously implicated in the generation of wakefulness and/or engaging cortical and motor activity (Fig 3A) [24–29]. Specifically, traces of axonal projections were identified in the medial septum (MS), basal forebrain (BF), lateral hypothalamus (LH), tuberomammillary nucleus (TMN), supramammilary nucleus (SuM), interpeduncular nucleus(IPN), and the ventrolateral periaqueductal gray (vlPAG) many of which play key roles in promoting wakefulness and/or inducing cortical activation (Fig 3B–3H). We also identified traces neural projections in the vestibular nucleus (VN) and in the ventral horn of the spinal cord, which both play key roles in engaging motor activity (Fig 3J and 3K) [30–32]. These findings indicate that SLD^GABA^ neurons are anatomically positioned to modulate wakefulness, cortical activity, and motor activity, which is consistent with our optogenetic data.

### Activation of SLD^GABA^ neurons triggers sleep attacks in *orexin*^*−/−*^ (i.e., narcoleptic) mice

**Fig 4:**
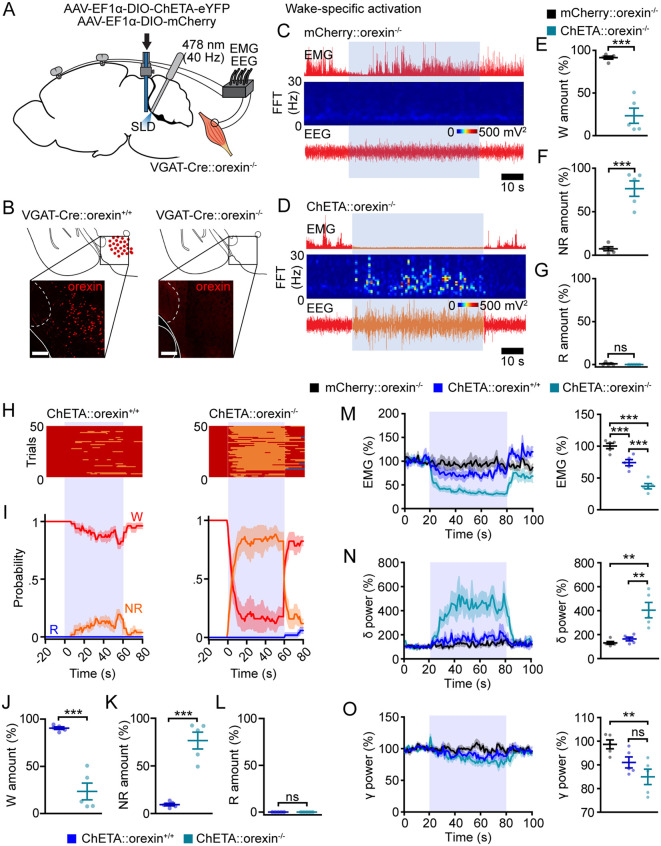
Activating SLD^GABA^ neurons in *orexin*^*−/−*^ mice potently suppresses wakefulness. **(A)** A schematic of optogenetic activation of SLD^GABA^ neurons coupled with EEG and EMG recordings in *VGAT::orexin*^*−/−*^ mice. **(B)** Schematic and microscope images showing the absence of orexin neuropeptide (red) from the lateral hypothalamus (LH) in *VGAT::orexin*^*−/−*^ (i.e., narcoleptic) mice. **(C** and **D)** Example polysomnogram with wake-specific activation of SLD^GABA^ neurons in *orexin*^*−/−*^ mice. Shown are EMG amplitude, EEG spectrogram, and EEG raw traces. **(E–G)** Mean percentages of wake, NREM, and REM sleep during 60 s wake-specific activation of SLD^GABA^ neurons (*mCherry::orexin*^*−/−*^ *n* = 5 and *ChETA::orexin*^*−/−*^ *n* = 5; unpaired *t* test). **(H)** Distribution of sleep-wake states in all wake-specific activation trials (60 s) aligned by the time of activation onset t = 0 s (*ChETA::orexin*^*+/+*^ *n* = 5 and *ChETA::orexin*^*−/−*^ *n* = 5). **(I)** Probability of wake, NREM, and REM sleep before, during and after the 60 s activation (*ChETA::orexin*^*+/+*^ *n* = 5 and *ChETA::orexin*^*−/−*^ *n* = 5). **(J–L)** Mean percentages of wake, NREM, and REM sleep during 60 s wake-specific activation (*ChETA::orexin*^*+/+*^ *n* = 5 and *ChETA::orexin*^*−/−*^ *n* = 5*;* unpaired *t* test). **(M) *LEFT*:** EMG activity before, during, and after 60 s wake-specific activation (*mCherry::orexin*^*−/−*^ *n* = 5, *ChETA::orexin*^*+/+*^ *n* = 5, and *ChETA::orexin*^*−/−*^ *n* = 5). ***RIGHT*:** Mean EMG activity during the 60 s wake-specific activation (*mCherry::orexin*^*+/+*^ *n* = 5, *ChETA::orexin*^*+/+*^ *n* = 5, and *ChETA::orexin*^*−/−*^ *n* = 5*;* unpaired *t* test). **(N** and **O) *LEFT*:** δ and γ EEG activity before, during and after 60 s wake-specific activation (*mCherry::orexin*^*−/−*^ *n* = 5, *ChETA::orexin*^*+/+*^ *n* = 5, and *ChETA::orexin*^*−/−*^ *n* = 5). ***Right*:** Mean δ and γ EEG activity during the 60 s wake-specific activation (*mCherry::orexin*^*−/−*^ *n* = 5, *ChETA::orexin*^*+/+*^ *n* = 5, and *ChETA::orexin*^*−/−*^ *n* = 5*;* unpaired *t* test). EEG bands: δ (delta, 0.5–4 Hz) and γ (gamma, 30-100 Hz). Blue patches indicate time of laser-ON period. All error bars and shades represent ±s.e.m. ** p < 0.01 *** p < 0.001 indicate significant differences. Coronal brain schematics are from Allen Brain Atlas 3rd edition. The data underlying this Figure can be found in S3 Data.

Orexin is a neurotransmitter produced by neurons in the LH, which send axonal projections across the brain to support wakefulness [16]. In narcolepsy, the loss of orexin signals destabilizes wakefulness, making this state vulnerable to sleep disturbances [15,33–35]. Previous work demonstrates that loss of orexin impacts how SLD neurons control arousal states [14,16,17]. For example, our recent work demonstrated that glutamate neurons in the SLD, which generate REM sleep muscle atonia in *orexin*^*+/+*^ (i.e., healthy) mice, promote cataplexy in *orexin*^*−/−*^ (i.e., orexin knockout, narcoleptic) mice. In this current study, we decided to investigate how the loss of orexin impacts the function of SLD^GABA^ neurons. We hypothesized that activation of the SLD^GABA^ neurons in *orexin*^*−/−*^ mice will potently suppress wakefulness and induce sleep attacks. To test this hypothesis, we bilaterally injected AAV-EF1a-DIO-ChETA-eYFP or AAV-EF1a-DIO-mCherry into the SLD of male *VGAT-Cre::orexin*^*−/−*^ mice. This newly developed mouse line behaves identically to ‘pure’ *orexin*^*−/−*^ mice and exhibits both sleep attacks and cataplexy [36]. EEG/EMG electrodes and optic fibers were implanted to manipulate GABA neurons in the SLD while monitoring sleep-wake states in *VGAT-Cre::orexin*^*−/−*^ mice (Fig 4A and 4B).

Using a closed-loop protocol, we optically activated SLD^GABA^ neurons (478 nm, 5 ms pulses at 40 Hz) during each arousal state in *orexin*^*−/−*^ mice. We found that activation of SLD^GABA^ neurons during wakefulness triggered a sudden entrance into NREM sleep, which was not observed in the mCherry control group (Fig 4C–4G). Intriguingly, we found that the effect of SLD^GABA^ neuron activation was different between *orexin*^*−/−*^ mice and *orexin*^*+/+*^ mice. Specifically, we show that the activation of SLD^GABA^ neurons during wakefulness had a stronger wake-suppressing effect in *orexin*^*−/−*^ than in *orexin*^*+/+*^ mice (Fig 4H–4L). In addition, we also found that activation caused a greater reduction in the cortical and motor activity in *orexin*^*−/−*^ mice (Figs 4M–4O and S4O–S4T). Together, these findings demonstrate that SLD^GABA^ neuron activation suppresses wakefulness and the cortical and motor features that support it, but the loss of orexin signaling potentiates these effects, causing the typical sleep-attack symptoms observed in narcoleptic patients.

We found that the wake→NREM sleep transitions induced by the activation of SLD^GABA^ neurons in *orexin*^*−/−*^ animals were distinguishable from the wake→NREM sleep transitions observed in *orexin*^*+/+*^ animals by how quickly they occurred. In *orexin*^*−/−*^ mice, the activation consistently and rapidly terminated wakefulness into NREM sleep (i.e., ~5 s) (Fig 4H–4L). The *orexin*^*−/−*^ mice remained in NREM sleep throughout the duration of activation (60 s) and consistently and rapidly returned to wakefulness at the offset of activation (Fig 4D, 4H, and 4I), similar to what happened during naturally occurring sleep-attacks. In addition, we found that activation in *orexin*^*−/−*^ mice could trigger a sleep attack regardless of the animal’s behavior and location at the onset of activation. In *orexin*^*+/+*^ mice, wake→NREM sleep transitions occur when animals are settled in their nests [37]. However, in *orexin*^*−/−*^ mice, the activation of SLD^GABA^ neurons induced a rapid entrance into NREM sleep even when the animals were active outside of the nest (S2 Video). S2 Video is a typical behavioral example showing that activation triggers an abrupt and direct wake→NREM sleep transition during a period of active wakefulness. Abrupt intrusions of NREM sleep into wakefulness are the defining feature of sleep attacks in human and murine narcolepsy [33,37,38]; therefore, we reasoned that the activation of SLD^GABA^ neurons induced sleep attacks in *orexin*^*−/−*^ mice.

Next, we investigated whether the cortical activity during the activation-induced NREM sleep resembles that of spontaneous sleep attacks in *orexin*^*−/−*^ mice. We found that the activation-induced NREM sleep had an EEG activity comparable to that of spontaneous sleep attacks, further supporting that the activation of SLD^GABA^ neurons induces sleep attacks in *orexin*^*−/−*^ mice (S5D–S5F Fig). By comparing the EEG activity, we further validated that activation of SLD^GABA^ neurons did not induce wake→REM sleep transitions, nor did it induce transition into cataplexy in *orexin*^*−/−*^ mice (S5A–S5C and S5G Fig). In addition, we found that the activation-induced state could immediately be terminated by tactile stimulation (i.e., touching a mouse with a paintbrush) (S5H and S5I Fig). We previously showed that an episode of cataplexy cannot be terminated by tactile stimulation [9]. Collectively, our findings demonstrate that activation of SLD^GABA^ neurons induces sleep attacks in *orexin*^*−/−*^ mice.

Finally, we activated SLD^GABA^ neurons selectively during both NREM and REM sleep in *orexin*^*−/−*^ mice. Here, we found no difference between genotypes in how activation of SLD^GABA^ neurons changed behavior during either NREM or REM sleep. As in *orexin*^*+/+*^ mice, we found that activation of SLD^GABA^ neurons during NREM sleep lengthened NREM sleep and delayed the entrance into wakefulness in *orexin*^*−/−*^ mice. The magnitude of this effect was similar in both genotypes (S4A–S4D Fig). Activating SLD^GABA^ neurons during REM sleep had no effect in either *orexin*^*+/+*^ or *orexin*^*−/−*^ mice (S4E and S4F Fig). Lastly, we found that neither motor nor cortical activity was impacted by their activation during NREM and REM sleep in either *orexin*^*+/+*^ or *orexin*^*−/−*^ mice (S4G–S4N Fig). These data, therefore, indicate that the activation of SLD^GABA^ neurons reinforces NREM sleep specifically.

### Silencing SLD^GABA^ neurons terminates sleep attacks by promoting wakefulness in *orexin*^*−/−*^ mice

**Fig 5:**
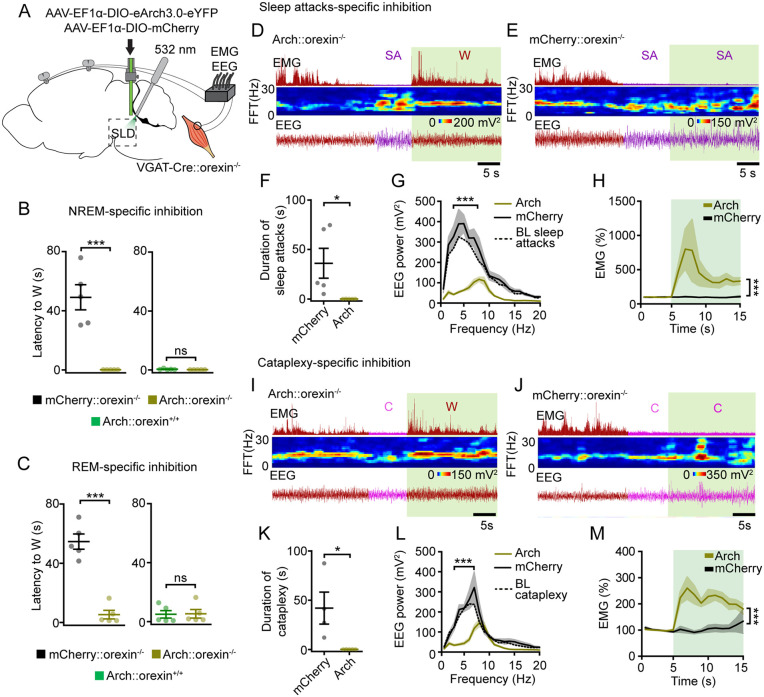
Silencing SLD^GABA^ neurons in orexin^−/−^ mice rescues the animals from sleep attacks and cataplexy. **(A)** A schematic of optogenetic silencing of SLD^GABA^ neurons coupled with EEG and EMG recordings in *VGAT::orexin*^*−/−*^ mice. **(B)** Latency to wake from NREM sleep upon inhibition (*mCherry::orexin*^*−/−*^ *n* = 5, *Arch::orexin*^*−/−*^ *n* = 5, and *Arch::orexin*^*+/+*^ *n* = 5; unpaired *t* test). **(C)** Latency to wake from REM sleep upon inhibition (*mCherry::orexin*^*−/−*^ *n* = 5, *Arch::orexin*^*−/−*^ *n* = 5, and *Arch::orexin*^*+/+*^ *n* = 5; unpaired *t* test). **(D** and **E)** Example polysomnograms with SLD^GABA^ neurons during sleep attacks. Shown are EMG amplitude, EEG spectrogram, and EEG raw traces. **(F)** Duration of sleep attacks upon inhibition (mCherry *n* = 5 and Arch *n* = 5; unpaired *t* test). **(G)** Power spectral density of EEG in response to the inhibition during sleep attacks (mCherry *n* = 5 and Arch *n* = 5; Two-way RM ANOVA with Bonferroni post-tests). **(H)** EMG activity before and during inhibition from sleep attacks (mCherry *n* = 5 and Arch *n* = 5; Two-way ANOVA with Bonferroni post-test). **(I** and **J)** Example polysomnograms with inhibition of SLD^GABA^ neurons during cataplexy. **(K)** Duration of cataplexy upon inhibition (mCherry *n* = 5 and Arch *n* = 5; unpaired *t* test). **(L)** Power spectral density of EEG in response to inhibition during cataplexy (mCherry *n* = 5 and Arch *n* = 5; Two-way RM ANOVA with Bonferroni post-tests). **(M)** EMG activity before and during inhibition from cataplexy (mCherry *n* = 5 and Arch *n* = 5; Two-way ANOVA with Bonferroni post-test). Green patches indicate time of laser-ON period. All error bars and shades represent ±s.e.m. * p < 0.05, ** p < 0.01, *** p < 0.001 indicate significant differences. The data underlying this Figure can be found in S4 Data.

Next, we wanted to determine if silencing SLD^GABA^ neurons can rescue narcoleptic animals from sleep attacks (Fig 5A). First, we investigated whether wakefulness produced by silencing SLD^GABA^ neurons differs between *orexin*^*−/−*^ and *orexin*^*+/+*^ mice. We found that silencing SLD^GABA^ neurons had similar wake-promoting effects in both *orexin*^*+/+*^ and *orexin*^*−/−*^ mice. As in *orexin*^*+/+*^ mice, we found that silencing SLD^GABA^ neurons triggered immediate wakefulness from both NREM and REM sleep in *orexin*^*−/−*^ mice and the latency to wakefulness did not differ between the genotypes (Fig 5B and 5C). In addition, we found that the wakefulness triggered by silencing SLD^GABA^ neurons had cortical activity and motor activity that was comparable between *orexin*^*−/−*^ and *orexin*^*+/+*^ mice (S6A–S6H Fig). We also found that silencing SLD^GABA^ neurons in *orexin*^*−/−*^ mice prolonged wakefulness and increased the probability of wakefulness across time. The magnitude of this effect was not significantly different from that observed in *orexin*^*+/+*^ mice (S6I and S6J Fig). Finally, we found that silencing SLD^GABA^ neurons during wakefulness enhanced both cortical and motor indices of wakefulness to the same extent in both *orexin*^*−/−*^ and *orexin*^*+/+*^ mice (S6K–S6P Fig). Together, these data show that the silencing SLD^GABA^ neurons promotes wakefulness and the cortical and motor features that support it, but these effects are unaltered by the loss of orexin signaling.

Because the activation of SLD^GABA^ neurons promotes sleep attacks in *orexin*^*−/−*^ mice, we wanted to test whether silencing these neurons could rescue animals from sleep attacks. We tested this by silencing SLD^GABA^ neurons at the onset of individual sleep attacks in *orexin*^*−/−*^ mice. We found that silencing SLD^GABA^ neurons immediately rescued the animals from sleep attacks into alert and motorically engaged wakefulness, which was not observed in the mCherry control group (Fig 5D–5H). These findings, in combination with the activation data (Fig 4), indicate that SLD^GABA^ neurons are both necessary and sufficient to generate sleep attacks.

Lastly, we wanted to determine if silencing SLD^GABA^ neurons could also rescue narcoleptic animals from cataplexy. Surprisingly, we found that silencing SLD^GABA^ neurons at the onset of cataplexy immediately rescued the animal into wakefulness (Fig 5I and 5K) by reinstating brain and motor activity (Fig 5L and 5M). Such behavioral effect was not observed in the mCherry control group (Fig 5J–5M). Because we showed that silencing SLD^GABA^ neurons enhances motor activity in both *orexin*^*−/−*^ and *orexin*^*+/+*^ mice (Figs 1Q and S6P), we propose that silencing SLD^GABA^ neurons rescues cataplexy by engaging motor activity.

## Discussion

**Fig 6:**
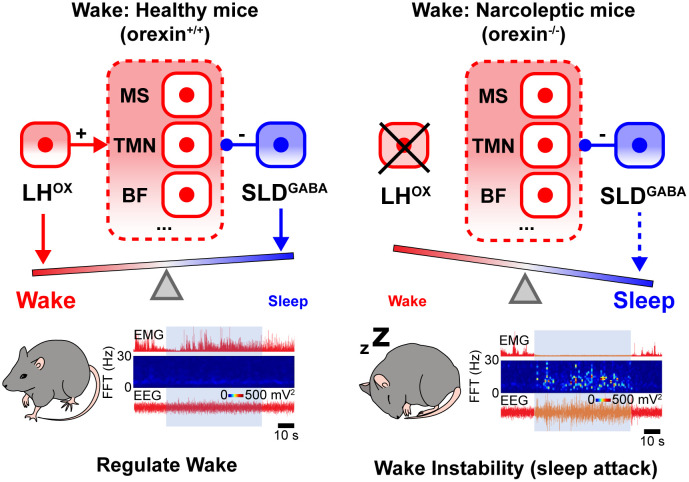
A proposed mechanism by which SLD^GABA^ neurons suppress wakefulness in healthy and narcoleptic animals. This schematic illustrates how the balance of wake-promoting signals from the LH orexin (LH^OX^) neurons and wake-suppressive signals from the SLD^GABA^ neurons may determine the changes in arousal states. In healthy mice (*orexin*^*+/+*^), the wake-promoting signal mediated by the LH^OX^ neurons predominates over the wake-suppressive signal mediated by the SLD^GABA^ neurons, generating a stable wakefulness. Conversely, in narcoleptic mice (*orexin*^*−/−*^), orexin-mediated wake-promoting signals are absent, allowing the SLD^GABA^ neurons to produce a disproportionately strong suppression of wakefulness, resulting in wake instability seen as sleep attacks.

Our study is the first to characterize the functional role of SLD^GABA^ neurons in sleep-wake control and establishes their link to the pathophysiology of narcolepsy. Through precise optogenetic manipulation, we found that SLD^GABA^ neurons function to suppress wakefulness (Figs 1 and 2). Next, we found traces of SLD^GABA^ axonal projections to wake-promoting brain regions, positioning SLD^GABA^ neurons as a potential nucleus in the neural circuits controlling arousal state transitions (Fig 3). Finally, we demonstrated that activation of SLD^GABA^ neurons induces pathological sleepiness, manifesting as sleep attacks in narcoleptic animals and that silencing of these neurons rescue them from this debilitating symptom (Figs 4–6).

The SLD has long been viewed as a core REM sleep-promoting structure. Specifically, GABA neurons in the SLD were hypothesized to promote REM sleep by inhibiting REM-off neurons in the vlPAG. This was supported by neuroanatomical and lesion-based evidence [14]. However, a subsequent study using a cell-specific genetic disruption approach showed that deletion of GABA neurotransmission in the SLD did not yield a clear effect on REM sleep [11]. Thus, the specific causal contribution of SLD^GABA^ neurons to arousal state control has remained speculative. By using cell-type-specific optogenetics in genetically defined populations, we demonstrate that SLD^GABA^ neurons suppress wakefulness rather than promote specific sleep states. Several lines of evidence support this conclusion. First, silencing SLD^GABA^ neurons promotes wakefulness from NREM and REM sleep, while activating the same neurons promotes NREM sleep from wakefulness (Figs 1 and 2), indicating that their influence extends beyond any single sleep stage. Second, silencing these neurons during existing wakefulness enhances arousal intensity by increasing cortical gamma activity and motor engagement (Figs 1 and 2). Together, these findings support a prominent wake-suppressive role for SLD^GABA^ neurons. However, our strategy broadly manipulated SLD^GABA^ neurons, which may have masked functional diversity within this population. We cannot exclude the possibility that distinct SLD^GABA^ subpopulations contribute to additional functions, including REM-state gating, or broader aspects of pontine state control [14,21,23,39]. Further experiments will need to dissect the functions of subpopulations of SLD^GABA^ neurons based on axonal projection profiles and cellular mechanisms.

This wake-suppressive function aligns with the broader organizational principles of sleep-wake control, where inhibitory mechanisms balance excitatory arousal systems to enable stable state transitions [14,40–43]. The SLD^GABA^ neurons appear to function as a “brake” on wakefulness, allowing controlled transitions into sleep states when arousal drive diminishes. This regulatory mechanism may be particularly important during vulnerable periods when wake stability is challenged, such as during sleep deprivation or circadian misalignment, or during pathologic conditions like narcolepsy [15,44,45]

Our genetically-assisted anatomical mapping reveals that SLD^GABA^ neurons possess the neuroanatomical potential to modulate multiple aspects of arousal. We found traces of SLD^GABA^ axonal projections to various wake-promoting nuclei across the brain [24–29] (Fig 3). Our findings suggest that SLD^GABA^ neurons may suppress cortical activation by targeting brain regions such as the MS and BF, while dampening global wake-promoting drive through targeting brain regions including LH and TMN [24,25,27,28,46]. Such widespread distribution of SLD inhibitory projections likely accounts for the potent wake-suppressive effects we observed. Our interpretation is further supported by a previous work which showed that the GABA-expressing Crhbp^+^ neurons in the SLD project to a wake-promoting region (i.e., the BF), and chemogenetic activation of these neurons suppresses wakefulness and promotes NREM sleep [47]. Traces of SLD^GABA^ axonal projections were also observed in motor-related areas, including the VN and the spinal cord. This may underlie the ability of SLD^GABA^ neurons to modulate motor activity during wakefulness [31]. Together, these distributed projections to both arousal and motor systems suggest a role for SLD^GABA^ neurons in maintaining stable state control and preventing partial or mixed arousal states that could destabilize sleep-wake boundaries.

However, anatomical tracing alone does not establish synaptic connectivity. In addition, many of these potential target brain regions are functionally heterogeneous and contribute to processes beyond sleep-wake regulation. For example, the BF is involved in attention, learning, and memory [48]; the LH regulates feeding and motivated behaviors [49]; the TMN participates in thermoregulation [50] and stress responses [51]. Furthermore, some of the target brain regions contain sleep-inducing neurons. For example, neurotensin neurons in vlPAG promote NREM sleep [52]; GABA neurons in BF promote NREM sleep [25]; Melanin concentrating hormone (MCH) neurons in LH promote REM sleep [53]. Thus, future studies will be required to determine which specific neural population in these target brain regions receives synaptic input from the SLD^GABA^ neurons and directly mediates wake suppression.

Previous work has shown that the SLD also receives inputs from the cortex, forebrain, midbrain, pons, and hindbrain [54], and these inputs may interact with SLD^GABA^ neurons to modulate wakefulness. Finally, the SLD^GABA^ neurons potentially receive inputs from the neighboring glutamate neurons within the SLD (SLD^GLU^ neurons). These neurons promote REM sleep and control aspects of the state [9–11,19,39,55,56]. Hence, integration at the level of the SLD may coordinate both REM sleep initiation with wake suppression, ensuring smooth state transitions without arousal interference.

The ability of SLD^GABA^ neurons to suppress wakefulness can be beneficial for sleep stability and normal sleep-wake regulation, but it can also pose a threat to wakefulness stability when engaged at the wrong time, as seen in narcoleptic patients [57]. The hypothalamic orexin system normally provides a strong excitatory drive to maintain wake stability [33,58]. We found that in orexin-deficient mice (*orexin*^*−/−*^*)*, activation of SLD^GABA^ neurons triggers rapid sleep attacks—abrupt intrusions of NREM sleep during active wakefulness that closely resemble the clinical presentation of human narcolepsy (Fig 4). The activation of SLD^GABA^ neurons in *orexin*^*−/−*^ mice rapidly initiates NREM sleep even amid active waking behavior (i.e., eating and moving) [15], bypassing stereotypical sleep-preparatory behaviors (e.g., grooming and nesting) (S2 Video). This effect is significantly enhanced compared to *orexin*^*+/+*^ animals, suggesting that orexin loss removes a critical brake on SLD^GABA^ neuron function (Fig 4). Importantly, this effect does not appear to be due to an increased sleep drive, as narcoleptic and control mice exhibit similar overall sleep-wake amounts (S4U Fig) [15]. Previous work has demonstrated that the fragmented wakefulness observed in orexin-deficient mice is not due to abnormal sleep homeostasis and increased sleep pressure [15]. Instead, the fragmented behavior of *orexin*^*−/−*^ mice is best described as behavioral-state instability, with low thresholds for transitions between states. We hypothesize that when orexin signaling is lost, SLD^GABA^ neurons override residual wake-promoting signals, creating windows of wake instability and triggering abnormal sleep intrusions (Fig 6). This hypothesis explains why narcoleptic patients experience sleep attacks even during stimulating activities. The loss of orexin signals allows wake-suppressive mechanisms to dominate inappropriately.

Given the well-established wake-promoting role of LH orexin neurons, we propose that these neurons may inhibit the wake-suppressing SLD^GABA^ neurons through an indirect pathway involving an intermediate GABAergic relay. Studies have shown that hypothalamic orexin neurons send projections to the SLD and surrounding regions, indicating the potential for neuromodulatory control of SLD neurons [16,59]. In addition, local infusion of orexin into the pontine reticular formation (PnO), which includes the SLD, suppresses PnO neurons by enhancing local GABAergic signaling and promoting wakefulness. [17] These findings raise the possibility that orexin inputs could suppress SLD^GABA^ neurons indirectly via local GABAergic interneurons, highlighting the possible heterogeneity of SLD^GABA^ neurons and the involvement of complex microcircuits within the SLD. In parallel, orexin neurons also strongly innervate the vlPAG, and vlPAG GABA neurons provide inhibitory GABAergic input to the SLD, suggesting another indirect pathway through which orexin neurons may regulate SLD activity [14,18,60]. Loss of orexin signaling could therefore disrupt SLD^GABA^ neurons through multiple indirect neural pathways. However, these indirect pathways remain speculative as the relevant synaptic connectivity and their functional role have not yet been established. The orexin neurons may also interact with SLD^GABA^ neurons at a postsynaptic level. Orexin neurons project broadly throughout the brain, many of which overlap with the projection pattern of the SLD^GABA^ neurons [16]. Orexin peptide has been shown to attenuate GABAergic inhibitory postsynaptic currents and reduce GABA_A_-receptor-mediated hyperpolarization via orexin-1 receptor activation [61]. This evidence suggests that orexin may counteract SLD^GABA^–mediated inhibition at the postsynaptic level. Together, these observations suggest that the relationship between LH orexin neurons and SLD^GABA^ neurons is complex and may involve the functional heterogeneity among SLD^GABA^ subpopulations, multiple indirect circuit mechanisms, and downstream postsynaptic interaction.

Finally, our discovery that silencing SLD^GABA^ neurons can rescue both sleep attacks and cataplexy (Fig 5) opens new therapeutic avenues for narcolepsy treatment. These two cardinal symptoms of narcolepsy –sleep attacks and cataplexy—are distinct physiological states [37]. The ability of SLD^GABA^ silencing to terminate both symptoms raises the possibility that these neurons can contribute to overlapping neural pathways. While SLD^GLU^ neurons drive cataplexy through REM sleep muscle atonia mechanisms, we hypothesize that the SLD^GABA^ neurons may interact with SLD^GLU^ neurons and contribute to generating cataplexy. Additional studies will be needed to define the precise circuit mechanism through which SLD^GABA^ neurons influence both sleep attacks and cataplexy. Although our optogenetic experiments demonstrated that acute silencing of the SLD^GABA^ neurons can rapidly terminate both sleep attacks and cataplexy, establishing their therapeutic potential requires determining whether sustained suppression of these neurons can reduce the overall emergence of these symptoms. Accordingly, an important future goal will be to employ a chemogenetic approach to test whether prolonged inhibition of SLD^GABA^ neurons can consolidate wakefulness and alleviate symptoms of narcolepsy.

Our study provides definitive evidence for SLD^GABA^ neuron function; however, several important questions remain. First, the precise mechanisms by which orexin loss potentiates SLD^GABA^ function require further investigation. While outside of the aim of our current study, direct recordings from SLD^GABA^ neurons in orexin-deficient animals could reveal whether orexin loss alters their intrinsic excitability, synaptic inputs, or both. Second, the upstream signals that normally regulate SLD^GABA^ activity in healthy animals remain unclear. Identifying these regulatory mechanisms could reveal how environmental and circadian factors influence sleep-wake stability. Third, the present study was conducted exclusively in male mice to maintain consistency with prior work; hence, it remains unknown whether sex-dependent mechanisms influence SLD^GABA^-medicated control of arousal and narcolepsy-related symptoms. Finally, the thorough investigation of the interaction between the SLD^GABA^ and SLD^GLU^ neurons in both health and disease states would identify what makes the SLD essential for the regulation of the sleep-wake cycle.

## Materials and methods

### Ethics statement

All procedures for this study were approved by the University of Toronto Animal Care Committee (*Protocol: 20011374*) and were in accordance with the Canadian Council on Animal Care.

### Animals and surgery

Experiments were conducted on 3 different strains of adult male mice (>5 weeks old, average weight: 21.2 ± 0.2 g). We used 33 *VGAT-Cre* mice (i.e., 129S6/SvEvTac background, provided by Dr. Adamantidis), 5 VGAT-Cre::TdTomato mice (i.e., VGAT-Cre mice crossed with Ai14 mice with C57BL6 background purchased from Jackson Laboratory), and 19 *VGAT-Cre::orexin*^*−/−*^ mice (i.e., VGAT-Cre mice crossed with *orexin*^*−/−*^ mice with background C57BL6 purchased from Jackson Laboratory). The genotype of the animals was verified using in-house genotyping, where we looked for the presence of a gene for Cre recombinase, TdTomato, and lacZ cassette. Male mice were used to maintain consistency with the majority of previous sleep-wake studies, which have predominantly focused on male animals due to concerns that fluctuations across the estrus cycle can influence sleep architecture [62]. This conservative approach was taken to allow direct comparison with our previously published work.

Animals were housed individually and maintained on a 12-hour light/dark cycle (lights on at 7:00). Both food and water were available ad libitum.

### Viral vector preparation

To optically excite or inhibit the activity of SLD^GABA^ neurons, we used viral vectors containing a transgene encoding for excitatory or inhibitory opsin. The viral vectors were developed by modifying the adeno-associated virus (AAV) to carry a gene encoding for either the ultra-fast-acting excitatory channel rhodopsin (i.e., ChETA) or the third-generation inhibitory archaerhodopsin (i.e., eArch3.0) under human elongation factor 1 alpha (EF1α) promoter primarily selective for neurons [63]. Both viral vectors were Cre-inducible (i.e., double-floxed inverted open reading frame, DIO). The transgenes encoding for ChETA and eArch3.0 were tagged with the fluorescent protein eYFP or mCherry, which assisted in validating the location of opsin expression from postmortem brain tissue. For sham control experiments, we used the same AAV construct but without the transgene encoding for the opsin. Thus, we used AAV5-EF1α-DIO-ChETA-eYFP for activation, AAV5-EF1α-DIO-eArch3.0-eYFP for inhibition, and AAV5-EF1α-DIO-mCherry for sham control. These viral vectors were packaged by the Viral Vector Core at the University of North Carolina. The final viral concentration was 4 × 10^12^ genome copies (gc) per mL.

### Viral vector injections

Under isoflurane anesthesia (0.5%–2%) and sterile conditions, appropriate AAV vector solution was loaded into a 50 µL Hamilton syringe and 200 nL of the AAV vector was infused using a microinfusion pump (Pump 11 Elite, Harvard Apparatus). The viral vectors were injected unilaterally or bilaterally through a cannula (Plastics One, Roanoke, VA) at a rate of 0.05 μL/min into the SLD region of *VGAT-Cre*, *VGAT-Cre;tdTomato*, or *VGAT-Cre;orexin*^*−/−*^ mice. The initial stereotaxic coordinate used for targeting the SLD was medio-lateral (ML): ± 0.9 mm; dorso-ventral (DV): 4.25 mm; rostro-caudal (RC): −5.34 mm based on the coordinates in the Allen brain atlas for rodents. However, to account for different variables that may exist between different stereotaxic apparatus and setups, the coordinates were empirically refined through pilot adjustments. After each experiment, the targeting accuracy was verified through histological confirmation (S1A Fig). Only animals with confirmed viral expression and optic fiber placement within the SLD were analyzed. These mice were given at least 2 weeks to recover, allowing the viral vector to spread in the target brain area.

### Polysomnographic electrodes and optical fibers implantation

After recovering from viral injection surgery, sterile surgery was performed to implant electroencephalogram (EEG) electrodes, electromyogram (EMG) electrodes and optic fibers. Electrodes and optic fibers were made prior to the implantation surgery. Four EEG electrodes and four EMG electrodes made of insulated, multi-stranded stainless-steel wire (AS 632; Cooner Wire) were soldered onto a custom-made microchip with gold-plated pins called “headplug” (Allied Electronics, Bristol, PA). Optic fibers were made using optic cables (200 μm, FT200EMT, ThorLabs) and ceramic ferrules (MM-FER2007C; PFP) or custom-made optic fibers were purchased (400 μm, Doric Lens). General anesthesia was induced and maintained via inhalation (isoflurane, 0.5%–2%). Craniotomy was made at RC: −5 mm, ML: ±0.9 mm from bregma for optic fiber implantations into the SLD and at RC: +1 mm, ML: ±1 mm and RC: −2 mm, ML: ± 3 mm from bregma for EEG electrode implantation. Premade optic fibers were lowered bilaterally or unilaterally above the SLD and secured with dental cement (C&B dental cement; Metabond). Four stainless-steel screws (P0090CE125; J.I. Morris) were used to implant four EEG electrodes. Two EEG electrodes were implanted into the frontal bone (RC: +1 mm, ML: ±1 mm from bregma), and two EEG electrodes were implanted into the parietal bone (RC: −2mm, ML ±3 mm from bregma). Two EMG electrodes were sutured into the right masseter muscles, and the other two were sutured into the nuchal muscles. Once all the electrodes were implanted, the headplug was secured on top of the skull using layers of dental cement (C&B dental cement; Metabond and Ketac cement; 3M ESPE). Upon completion of the surgery, mice were given at least two weeks to recover before undergoing behavior recording.

### Recording environment

During experiments, animals were housed in a custom-made plexiglass recording chamber (20 w x 35 l x 42 h cm) kept inside a sound-attenuated, ventilated, and illuminated 12:12 light/dark cycle (lights on 7:00; 110 lux) room. Studies have shown that emotional stimuli, including exercise and chocolate, increase the frequency of cataplexy in narcoleptic mice [38]. Thus, a running wheel was installed in the recording chamber for narcoleptic animals, and they received chocolate (4.5 g, Hershey’s Kisses) at the onset of the dark phase (19:00) when narcoleptic symptoms are most frequent [38].

### Optogenetics and sleep recording

#### Electrophysiological recordings.

EEG and EMG activities were recorded by attaching a lightweight electrogram cable (CW7117; CoonerWire) to the headplug which gives access to EEG and EMG electrodes. EEG and EMG signals were further amplified using Super-Z high impedance headstage preamplifier and BM-400 AC/DC bioamplifier (CWE). The EEG signal was bandpass filtered between 0.3 and 100 Hz. EMG signals were bandpass filtered between 30 Hz and 30 kHz. A 60 Hz notch filter was applied when necessary. All electrophysiological signals were sampled at 2 kHz, digitized (Spike2 software, 1,401 interface; Cambridge Electronic Design, Cambridge, UK), monitored and stored on a computer.

#### Optogenetic experiment.

The implanted optic fibers were connected to diode lasers (Laser Glow Technologies) using patch cords (200 μm diameter; Doric lens). 473 nm (blue, 10 mW/mm [2]) lasers were used for activation, and 532 nm (green, 10 mW/mm [2]) lasers were used for inhibition. Both lasers were connected to a computer through a 1401 interface (CED). Stimulation instructions were generated via Spike2 software (CED), which sent digital signals to the lasers to generate appropriate patterns of the light pulses. The Spike2 software program had the capacity to conduct video recording, electrophysiological recording, and optogenetic manipulation simultaneously, which allowed us to manipulate the SLD^GABA^ neurons selectively during a given arousal state in real-time. The animals underwent optogenetic manipulation during the light phase (14:00–17:00) and dark phase (20:00–23:00) to capture sufficient samples from all arousal states. The SLD^GABA^ neurons were stimulated with 5-ms blue light pulses at 40 Hz for activation or continuous green light pulse for inhibition. We decided to use 40 Hz pulse frequencies for activation because a previous study has demonstrated that SLD^GABA^ neurons fire up to 50 Hz in vitro [22] and these neurons can fire at 40 Hz in vivo when a continuous excitatory stimulus is delivered [23]. To ensure that light delivery followed stable transitions between arousal states, stimulation was triggered only after animals maintained a given state for a predefined duration: 20 s for NREM sleep, 5 s for REM sleep, and 10 s for wakefulness. These thresholds were determined based on the typical transition dynamic between arousal states [20]. The success rate of the closed-loop illumination (defined as accurate state detection and stimulus delivery within the intended arousal state, calculated from all optogenetic manipulation trials conducted in this study) was 99.16% (99.17% for NREM-specific illumination, 98.43% for REM-specific illumination and 100% for wake-specific illumination). Upon completion of closed-loop experiments, all inaccurate trials caused by human or system error have been removed from the analysis.

### Data analysis

#### State analysis.

In VGAT-Cre mice, three behavioral states were characterized—wakefulness, NREM, and REM sleep. Wakefulness was characterized by high-frequency and low-voltage EEG signals coupled with moderate to high levels of EMG activity. NREM sleep was characterized by high-voltage and low-frequency (0.5–4 Hz) EEG signals and minimal EMG activity. REM sleep was characterized by low-voltage and high-frequency theta-like (4–8 Hz) EEG activity, and muscle atonia with periodic muscle twitches. Arousal states were visually identified and scored in 5-s epochs (Spike2 Script) by an experimenter blinded to the experimental conditions. We verified the accuracy of our visual scoring by computing the power spectrum of EEG frequency bands (Fast Fourier Transform (FFT) analysis, Hamming windows, 1,024 sampling points). State probability was obtained by aligning scored states from all successive light manipulation trials by the time of light onsets and calculating the odds for each arousal state.

#### EMG analysis.

Raw EMG signals were full wave rectified, integrated, and quantified. Average EMG activity was quantified in 1-s epochs during each trial of light stimulation. EMG activity during the stimulation was normalized to the baseline EMG activity 10 s before the onset of the stimulation.

#### EEG analysis.

EEG power spectrum analysis was conducted using Spike2 software FFT, Hamming windows, FFT size: 1,024) to identify the frequency content of the EEG signals. EEG frequency bands were grouped into 0.5–4 Hz (delta, δ), 4–8 Hz (theta, θ), 8–12 Hz (alpha, α), 12–30 Hz (beta, β), and 30–100 Hz (gamma, γ) [64]. The changes in EEG power across time were sampled at 1 s intervals. The EEG power of a given frequency band during the stimulation was normalized to the baseline EEG power 10 s before the onset of the stimulation.

### Tissue preparation

After behavioral experiments, the animals were given an injection of tribromoethanol (300 mg/kg mouse, *i.p.*) followed by inhalation of isoflurane. Once the animals no longer responded to a strong toe pinch, they were transcardially perfused with 0.1 M PBS followed by a 4% solution of paraformaldehyde (PFA). The brain was extracted, stored in the 4% PFA solution overnight and then switched to a 30% sucrose in 0.1 PB for a few days for cryoprotection. Once the brain had sunk into the sucrose solution, the tissue was prepared for sectioning and immunohistochemistry.

### Immunohistochemistry

The fixed, cryoprotected brains were embedded in optimal cutting temperature (OCT) compound and fast-frozen on dry ice. Then, the brains were sectioned into 40 μm slices using a cryostat (Leica Microsystems). To visualize the proteins of interest, we performed immunohistochemistry on the sectioned tissue. The brain sections were washed in 0.1 M PBS (pH 7.4) three times for five min per wash then incubated in blocking buffer (10% goat serum, 0.4% Triton-X in PBS) for 1 hour in room temperature (RT). After washing out the buffer solution, the brain sections were incubated in a primary antiserum for 24–48 hours at 4 °C. For orexin-A staining, we used a rabbit polyclonal antiserum specific for orexin-A (1:2000; Phoenix Pharmaceuticals). To label the virally expressed opsin when necessary (i.e., when the innate fluorescence from the eYFP tag is weak), we used a monoclonal mouse antiserum specific for the eYFP proteins (1:2000; Millipore). Once complete, the sections were incubated in fluorescent secondary antiserum (Alexa-488 or Alexa-647; against appropriate species IgG; 1:500; Jackson Immunoresearch Labs) for 1 hour in RT then washed in 0.1 M PBS. Brain sections were mounted on glass slides and cover-slipped with aqua-mount (VWR) in preparation for microscopy. Images were obtained using a fluorescent microscope (Axio Imager Z1, Zeiss) or a confocal microscope (Laser Scanning Confocal Microscope LSM800, Zeiss) using Zen software, and minimally processed to enhance brightness and contrast using ImageJ and Adobe Photoshop CS3.

### Statistical analysis

The statistical tests used for analyses are included in the Results section. All statistical analyses were conducted using GraphPad Prism (Version 5.00) and applied a critical two-tailed α value of *p* < 0.05. One-way or Two-way analysis of variance (ANOVA) was followed by Bonferroni *post* *hoc* comparisons. All data are presented as either average + standard error of the mean (SEM) unless otherwise indicated.

## Supporting information

S1 Fig Silencing SLD^GABA^ neurons induces cortical and motor arousal. **(A)** Schematic and Microscope image of the rostral and caudal SLD (coronal section) showing the area of viral expression (Arch-GFP, green) and location of the implanted optic fiber above the SLD (white line). **(B)** A schematic showing the area of viral expression and location of implanted optic fiber tips in the SLD (*n* = 5). **(C** and **E)** Power spectral density of EEG before and during NREM-specific and REM-specific inhibition (*n* = 5; Two-way RM ANOVA with Bonferroni post-test). **(D** and **F)** EMG activity before and during NREM-specific and REM-specific inhibition (mCherry *n* = 5 and Arch *n* = 5; Two-way ANOVA with Bonferroni post-test). **(G) *LEFT*:** EMG activity before, during and after 20 s wake-specific inhibition (mCherry *n* = 5 and Arch *n* = 5). ***RIGHT*:** Mean EMG activity during the 20 s inhibition (mCherry *n* = 5 and Arch *n* = 5; unpaired *t* test). **(H–L) *LEFT*:** δ, θ, α, β, and γ EEG activity before, during and after 20 s wake-specific inhibition (mCherry *n* = 5 and Arch *n* = 5). ***RIGHT*:** Mean δ, θ, α, β, and γ EEG activity during the 20 s inhibition (mCherry *n* = 5 and Arch *n* = 5; unpaired *t* test). **(M)** Animal behavior during wake-specific inhibition (mCherry *n* = 5, 53 trials and Arch *n* = 5, 48 trials). EEG bands: δ (delta, 0.5–4 Hz), θ (theta, 4–8 Hz), α (alpha, 8–12 Hz), β (beta, 12–30 Hz), and γ (gamma, 30–100 Hz). Green patches indicate time of the inhibition (for eArch3.0) or sham laser inhibition (for mCherry). All error bars and shades represent ±s.e.m. * p < 0.05, ** p < 0.01, *** p < 0.001 indicate significant differences. Abbreviations: medio-lateral (ML), dorso-ventral (DV), rostro-caudal (RC), superior cerebellar peduncle (scp), trigeminal motor nucleus (Mo5), and 4th ventricle (4V). Atlas coordinates and coronal brain schematics are from Allen Brain Atlas version 3rd edition. The data underlying this Figure can be found in S5 Data.
(TIF)

S2 Fig Sleep-wake states were unaffected by green (532 nm, continuous) or blue (478 nm, 40 Hz, 5 ms pulses) laser stimulation to mCherry-expressing SLD^GABA^ neurons. **(A)** Average latency to wake from NREM sleep during baseline and 532 nm laser stimulation (continuous; *n* = 5; paired *t* test) **(B)** Power spectral density of EEG before and during 532 nm laser stimulation from NREM sleep (*n* = 5; Two-way RM ANOVA). **(C)** EMG activity across NREM sleep during baseline and 532 nm laser stimulation (*n* = 5; Two-way RM ANOVA)**. (D)** Average latency to wake from NREM sleep during baseline and 478 nm laser stimulation (*n* = 4; paired *t* test) **(E)** Power spectral density of EEG before and during 478 nm laser stimulation from NREM sleep (*n* = 4; Two-way RM ANOVA). **(F)** EMG activity across NREM sleep during baseline and 478 nm laser stimulation (*n* = 4; Two-way RM ANOVA). **(G)** Average latency to wake from REM sleep during baseline and 532 nm laser stimulation (*n* = 5; paired *t* test). **(H)** Power spectral density of EEG before and during 532 nm laser stimulation from REM sleep (*n* = 5; Two-way RM ANOVA). **(I)** EMG activity across REM sleep during baseline and 532 nm laser stimulation (*n* = 5; Two-way RM ANOVA). **(J)** Average latency to wake from REM sleep during baseline and 478 nm laser stimulation (*n* = 4; paired *t* test). **(K)** Power spectral density of EEG before and during 478 nm laser stimulation from REM sleep (*n* = 4; Two-way RM ANOVA). **(L)** EMG activity across REM sleep during baseline and 478 nm laser stimulation (*n* = 4; Two-way RM ANOVA). **(M) *LEFT*:** EMG activity before, during and after 532 nm laser stimulation during wakefulness compared to baseline wakefulness (*n* = 5; Two-way RM ANOVA). ***RIGHT*:** Mean EMG activity during the 532 nm laser stimulation compared to baseline wakefulness (*n* = 5; paired *t* test). **(N–R) *LEFT*:** δ, θ, α, β, and γ EEG activity before, during, and after 532 nm laser stimulation from wakefulness compared to baseline wakefulness (*n* = 5). ***RIGHT*:** Mean δ, θ, α, β, and γ EEG activity during the 532 nm laser stimulation compared to baseline wakefulness (*n* = 5; paired *t* test). **(S) *LEFT*:** EMG activity before, during and after 478 nm laser stimulation during wakefulness compared to baseline wakefulness (*n* = 5; Two-way RM ANOVA). ***RIGHT*:** Mean EMG activity during the 478 nm laser stimulation compared to baseline wakefulness (*n* = 5; paired *t* test). **(T–X) *LEFT*:** δ, θ, α, β, and γ EEG activity before, during, and after 478 nm laser stimulation from wakefulness compared to baseline wakefulness (*n* = 5). ***RIGHT*:** Mean δ, θ, α, β, and γ EEG activity during the 478 nm laser stimulation compared to baseline wakefulness (*n* = 5; paired *t* test). EEG bands: δ (delta, 0.5–4 Hz), θ (theta, 4–8 Hz), α (alpha 8–12 Hz), β (beta, 12–30 Hz), and γ (gamma, 30–100 Hz). Green/Blue patches indicate time of laser stimulation. All error bars and shades represent ±s.e.m. The data underlying this Figure can be found in S6 Data.
(TIF)

S3 Fig 40 Hz activation of SLD^GABA^ neurons suppresses cortical and motor arousal. **(A)** A schematic showing optogenetic activation (478 nm, 40 Hz, 5 ms pulses) of SLD^GABA^ neurons coupled with EEG and EMG recordings. **(B)** Power spectral density of EEG before and during NREM-specific activation (*n* = 5; Two-way RM ANOVA). **(C)** EMG activity before and during NREM-specific activation (mCherry *n* = 4 and ChETA *n* = 5; Two-way ANOVA with Bonferroni post-test). **(D)** Power spectral density of EEG before and during REM-specific activation (*n* = 5; Two-way RM ANOVA with Bonferroni post-test). **(E)** EMG activity before and during REM-specific activation (mCherry *n* = 4 and ChETA *n* = 5; Two-way ANOVA). **(F) *LEFT***: EMG activity before, during, and after wake-specific activation (mCherry *n* = 4 and ChETA *n* = 5). ***RIGHT***: Mean EMG activity during the activation (mCherry *n* = 4 and ChETA *n* = 5; unpaired *t* test). **(G–K) *LEFT*:** δ, θ, α, β, and γ EEG activity before, during, and after wake-specific activation (mCherry *n* = 4 and ChETA *n* = 5). ***RIGHT*:** Mean δ, θ, α, β, and γ activity during the activation (mCherry *n* = 4 and ChETA *n* = 5; unpaired *t* test). EEG bands: δ (delta, 0.5–4 Hz), θ (theta, 4–8 Hz), α (alpha, 8–12 Hz), β (beta, 12–30 Hz), and γ (gamma, 30–100 Hz). Blue patches indicate time of laser stimulation. All error bars and shades represent ±s.e.m. * p < 0.05, ** p < 0.01 indicate significant differences. The data underlying this Figure can be found in S7 Data.
(TIF)

S4 Fig Activation of SLD^GABA^ neurons in *orexin*^*−/−*^ mice suppresses wakefulness into NREM sleep more potently than in *orexin*^+/+^ mice. **(A** and **B)** Latency to wake from NREM sleep upon activation of SLD^GABA^ neurons (478 nm, 40 Hz; *mCherry::orexin*^*−/−*^ *n* = 5, *ChETA::orexin*^*−/−*^ *n* = 5, and *ChETA::orexin*^*+/+*^ *n* = 5; unpaired *t* test). **(C** and **D)** Duration of NREM sleep upon activation (*mCherry::orexin*^*−/−*^ *n* = 5, *ChETA::orexin*^*−/−*^ *n* = 5, and *ChETA::orexin*^*+/+*^ *n* = 5; unpaired *t* test). **(E** and **F)** Latency to wake from REM sleep upon activation (478 nm, 40 Hz; *mCherry::orexin*^*−/−*^ *n* = 5, *ChETA::orexin*^*−/−*^ *n* = 5, and *ChETA::orexin*^*+/+*^ *n* = 5; unpaired *t* test). **(G** and **H)** EMG activity before and during NREM-specific activation (*mCherry::orexin*^*−/−*^ *n* = 5, *ChETA::orexin*^*−/−*^ *n* = 5, and *ChETA::orexin*^*+/+*^ *n* = 5; Two-way ANOVA). **(I** and **J)** Power spectral density of EEG during NREM-specific activation (*mCherry::orexin*^*−/−*^ *n* = 5, *ChETA::orexin*^*−/−*^ *n* = 5, and *ChETA::orexin*^*+/+*^ *n* = 5; Two-way ANOVA). **(K** and **L)** EMG activity before and during REM-specific activation (*mCherry::orexin*^*−/−*^ *n* = 5, *ChETA::orexin*^*−/−*^ *n* = 5, and *ChETA::orexin*^*+/+*^ *n* = 5; Two-way ANOVA). **(M** and **N)** Power spectral density of EEG during REM-specific activation (mCherry::orexin^−/−^ *n* = 5, *ChETA::orexin*^*−/−*^ *n* = 5, and *ChETA::orexin*^*+/+*^ *n* = 5; Two-way ANOVA). **(O) *LEFT*:** EMG activity before, during, and after 60 s wake-specific activation (*mCherry::orexin*^*−/−*^ *n* = 5, *ChETA::orexin*^*+/+*^ *n* = 5, and *ChETA::orexin*^*−/−*^ *n* = 5). ***RIGHT*:** Mean EMG activity during the activation (*mCherry::orexin*^*−/−*^ *n* = 5, *ChETA::orexin*^*+/+*^ *n* = 5, and *ChETA::orexin*^*−/−*^ *n* = 5; unpaired *t* test). **(P–T) *LEFT***: δ, θ, α, β, and γ EEG activity before, during and after 60 s wake-specific activation (*mCherry::orexin*^*−/−*^ *n* = 5, *ChETA::orexin*^*+/+*^ *n* = 5, and *ChETA::orexin*^*−/−*^ *n* = 5). ***RIGHT*:** Mean δ, θ, α, β, and γ EEG activity during the activation (*mCherry::orexin*^*−/−*^ *n* = 5, *ChETA::orexin*^*+/+*^ *n* = 5, and *ChETA::orexin*^*−/−*^ *n* = 5; unpaired *t* test). **(U)** The amount of NREM, wake, and REM sleep is comparable between narcoleptic and healthy animals during 20:00–23:00 (*orexin*^*−/−*^ *n* = 5, *orexin*^*+/+*^ *n* = 5). EEG bands: δ (delta, 0.5–4 Hz), θ (theta, 4–8 Hz), α (alpha, 8–12 Hz), β (beta, 12–30 Hz), and γ (gamma, 30–100 Hz). Blue patches indicate time of laser stimulation. All error bars and shades represent ±s.e.m. * p < 0.05, ** p < 0.01, *** p < 0.001 indicate significant differences.The data underlying this Figure can be found in S8 Data.
(TIF)

S5 Fig Activation of SLD^GABA^ neurons in *orexin*^*−/−*^ mice induces sleep attacks and not cataplexy. **(A)** Example polysomnogram during cataplexy. Shown are EMG amplitude, EEG spectrogram, and EEG raw traces. **(B)** Magnified polysomnogram during the time window shown in dotted box in (A). **(C)** Power spectral density of EEG during activation of SLD^GABA^ neurons compared to spontaneous cataplexy attack (*n* = 5; Two-way RM ANOVA with Bonferroni post-test) **(D)** Example polysomnogram during sleep attacks. **(E)** Magnified polysomnogram during the time window shown in dotted box in (D). **(F)** Power spectral density of EEG during activation of SLD^GABA^ neurons compared to spontaneous sleep attacks (*n* = 5; Two-way RM ANOVA). **(G)** Power spectral density of EEG during activation of SLD^GABA^ neurons compared to spontaneous NREM and REM sleep (*n* = 5; Two-way RM ANOVA with Bonferroni post-test). **(H)** A schematic summarizing the observation made from delivering tactile stimuli (i.e., touching a mouse with a paintbrush) during the activation of SLD^GABA^ neurons in *orexin*^*−/−*^ mice. ① animal is in baseline wake state with no activation, ② activation of SLD^GABA^ neurons induces transition into NREM sleep, ③ during the activation, physical stimulus is delivered to the animal, and ④ despite the sustained activation, the animal responded to the physical stimuli by waking up. **(I)** Example polysomnogram showing *orexin*^*−/−*^ mice that are undergoing the procedure depicted from (H) (*n* = 5). Shown are EMG amplitude and EEG raw traces. All error bars and shades represent ±s.e.m. ** p < 0.01 *** p < 0.001 indicate significant differences.
(TIF)

S6 Fig Silencing of SLD^GABA^ neurons in orexin^−/−^ mice promotes wakefulness and enhances cortical and motor indices of wakefulness like in orexin^+/+^ mice. **(A** and **B)** EMG activity before and during NREM-specific inhibition (532 nm; mCherry::orexin^−/−^ *n* = 5, Arch::orexin^−/−^ *n* = 5, and Arch::orexin^+/+^ *n* = 5; Two-way ANOVA with Bonferroni post-test). **(C** and **D)** Power spectral density of EEG during NREM-specific inhibition (*mCherry::orexin*^*−/−*^ *n* = 5, *Arch::orexin*^*−/−*^ *n* = 5, and *Arch::orexin*^*+/+*^ *n* = 5; Two-way ANOVA). **(E** and **F)** EMG activity before and during REM-specific inhibition (*mCherry::orexin*^*−/−*^ *n* = 5, *Arch::orexin*^*−/−*^ *n* = 5, and *Arch::orexin*^*+/+*^ *n* = 5; Two-way ANOVA). **(G** and **H)** Power spectral density of EEG during REM-specific inhibition (*mCherry::orexin*^*−/−*^ *n* = 5, *Arch::orexin*^*−/−*^ *n* = 5, and *Arch::orexin*^*+/+*^ *n* = 5; Two-way ANOVA with Bonferroni post-test). **(I)** Duration of wakefulness induced by 10 and 20 s inhibition (*orexin*^*+/+*^ *n* = 5 and orexin^−/−^ *n* = 5; one-way ANOVA with Tukey’s Multiple Comparison Test). **(J)** Probability of wakefulness in response to 10 and 20 s inhibition during wakefulness (orexin^+/+^ *n* = 5 and orexin^−/−^ *n* = 5; two-way ANOVA). **(K–O) *LEFT***: δ, θ, α, β, and γ EEG activity before, during, and after 20 s wake-specific inhibition (*mCherry::orexin*^*−/−*^ *n* = 5, *Arch::orexin*^*−/−*^ *n* = 5, and *Arch::orexin*^*+/+*^ *n* = 5). ***RIGHT*:** Mean δ, θ, α, β, and γ EEG activity during the 20 s inhibition (*mCherry::orexin*^*−/−*^ *n* = 5, *Arch::orexin*^*−/−*^ *n* = 5, and *Arch::orexin*^*+/+*^ *n* = 5; unpaired *t* test). **(P) *LEFT***: EMG activity before, during, and after 20 s wake-specific inhibition (*mCherry::orexin*^*−/−*^ *n* = 5, *Arch::orexin*^*−/−*^ *n* = 5, and *Arch::orexin*^*+/+*^ *n* = 5). ***RIGHT*:** Mean EMG activity during the 20 s inhibition (*mCherry::orexin*^*−/−*^ *n* = 5, *Arch::orexin*^*−/−*^ *n* = 5, and *Arch::orexin*^*+/+*^ *n* = 5; unpaired *t* test). EEG power bands: δ (delta, 0.5–4 Hz), Lθ (theta, 4–8 Hz), Hθ (alpha, 8–12 Hz), β (beta, 12–30 Hz), and γ (gamma, 30–100 Hz). Green patches indicate time of laser stimulation. All error bars and shades represent ±s.e.m. All error bars and shades represent ±s.e.m. * p < 0.05, ** p < 0.01, *** p < 0.001 indicate significant differences. The data underlying this Figure can be found in S9 Data.
(TIF)

S1 Data Numerical data used in panels of Figure 1. (XLSX)

S2 Data Numerical data used in panels of Figure 2. (XLSX)

S3 Data Numerical data used in panels of Figure 4. (XLSX)

S4 Data Numerical data used in panels of Figure 5. (XLSX)

S5 Data Numerical data used in panels of Figure S1. (XLSX)

S6 Data Numerical data used in panels of Figure S2. (XLSX)

S7 Data Numerical data used in panels of Figure S3. (XLSX)

S8 Data Numerical data used in panels of Figure S4. (XLSX)

S9 Data Numerical data used in panels of Figure S6. (XLSX)

S1 Video Silencing of SLD^GABA^ neurons induces wakefulness from sleep.  This video shows real-time recordings of animal behavior with simultaneous EEG and EMG activity during optogenetic silencing of SLD^GABA^ neurons in a healthy mouse. (MP4)

S2 Video Activation of SLD^GABA^ neurons in orexin^−/−^ mice induces sleep attacks. This video shows real-time recordings of animal behavior with simultaneous EEG and EMG activity during optogenetic activation of SLD^GABA^ neurons in a narcoleptic mouse. (MP4)

## Abbreviations

AAV: adeno-associated virus
ANOVA: analysis of variance
BF: basal forebrain
DV: dorso-ventral
EEG: electroencephalogram
EF1α: elongation factor 1 alpha
EMG: electromyogram
FFT: Fast Fourier Transform
LH: lateral hypothalamus
ML: medio-lateral
MPA: medial preoptic area
MS: medial septum
NREM: non-REM
OCT: optimal cutting temperature
PFA: paraformaldehyde
PnO: pontine reticular formation
RC: rostro-caudal
REM: rapid eye movement
RT: room temperature
SEM: standard error of the mean
SLD: sublaterodorsal tegmental nucleus
SLD^GABA^: GABA SLD neurons
SLD^GLU^: Glutamate SLD
vlPAG: ventrolateral periaqueductal gray
VN: vestibular nucleus.

## References

[1] Magoun HW. Bulbar inhibition and facilitation of motor activity. Science. 1944;100(2607):549–50. doi: 10.1126/science.100.2607.549 17758676

[2] Moruzzi G, Magoun HW. Brain stem reticular formation and activation of the EEG. Electroencephalogr Clin Neurophysiol. 1949;1(4):455–73. doi: 10.1016/0013-4694(49)90219-9 18421835

[3] Jouvet M. Research on the neural structures and responsible mechanisms in different phases of physiological sleep. Arch Ital Biol. 1962;100:125–206. 14452612

[4] Carter ME, Yizhar O, Chikahisa S, Nguyen H, Adamantidis A, Nishino S, et al. Tuning arousal with optogenetic modulation of locus coeruleus neurons. Nat Neurosci. 2010;13(12):1526–33. doi: 10.1038/nn.2682 21037585 PMC3174240

[5] Anaclet C, Ferrari L, Arrigoni E, Bass CE, Saper CB, Lu J, et al. The GABAergic parafacial zone is a medullary slow wave sleep-promoting center. Nat Neurosci. 2014;17(9):1217–24. doi: 10.1038/nn.3789 25129078 PMC4214681

[6] Qiu MH, Chen MC, Fuller PM, Lu J. Stimulation of the pontine parabrachial nucleus promotes wakefulness via extra-thalamic forebrain circuit nodes. Curr Biol. 2016;26(17):2301–12. doi: 10.1016/j.cub.2016.07.054 27546576 PMC5025760

[7] Vanini G, Watson CJ, Lydic R, Baghdoyan HA. Gamma-aminobutyric acid-mediated neurotransmission in the pontine reticular formation modulates hypnosis, immobility, and breathing during isoflurane anesthesia. Anesthesiology. 2008;109(6):978–88. doi: 10.1097/ALN.0b013e31818e3b1b 19034094 PMC2743234

[8] Xi MC, Morales FR, Chase MH. Evidence that wakefulness and REM sleep are controlled by a GABAergic pontine mechanism. J Neurophysiol. 1999;82(4):2015–9. doi: 10.1152/jn.1999.82.4.2015 10515993

[9] Torontali ZA, Fraigne JJ, Sanghera P, Horner R, Peever J. The sublaterodorsal tegmental nucleus functions to couple brain state and motor activity during REM sleep and wakefulness. Curr Biol. 2019;29(22):3803-3813.e5. doi: 10.1016/j.cub.2019.09.026 31679942

[10] Valencia Garcia S, Libourel P-A, Lazarus M, Grassi D, Luppi P-H, Fort P. Genetic inactivation of glutamate neurons in the rat sublaterodorsal tegmental nucleus recapitulates REM sleep behaviour disorder. Brain. 2017;140(2):414–28. doi: 10.1093/brain/aww310 28007991

[11] Krenzer M, Anaclet C, Vetrivelan R, Wang N, Vong L, Lowell BB, et al. Brainstem and spinal cord circuitry regulating REM sleep and muscle atonia. PLoS One. 2011;6(10):e24998. doi: 10.1371/journal.pone.0024998 22043278 PMC3197189

[12] Ford B, Holmes CJ, Mainville L, Jones BE. GABAergic neurons in the rat pontomesencephalic tegmentum: codistribution with cholinergic and other tegmental neurons projecting to the posterior lateral hypothalamus. J Comp Neurol. 1995;363(2):177–96. doi: 10.1002/cne.903630203 8642069

[13] Minert A, Devor M. Brainstem node for loss of consciousness due to GABA(A) receptor-active anesthetics. Exp Neurol. 2016;275 Pt 1:38–45. doi: 10.1016/j.expneurol.2015.10.001 26436687

[14] Lu J, Sherman D, Devor M, Saper CB. A putative flip-flop switch for control of REM sleep. Nature. 2006;441(7093):589–94. doi: 10.1038/nature04767 16688184

[15] Mochizuki T, Crocker A, McCormack S, Yanagisawa M, Sakurai T, Scammell TE. Behavioral state instability in orexin knock-out mice. J Neurosci. 2004;24(28):6291–300. doi: 10.1523/JNEUROSCI.0586-04.2004 15254084 PMC6729542

[16] Peyron C, Tighe DK, van den Pol AN, de Lecea L, Heller HC, Sutcliffe JG, et al. Neurons containing hypocretin (orexin) project to multiple neuronal systems. J Neurosci. 1998;18(23):9996–10015. doi: 10.1523/JNEUROSCI.18-23-09996.1998 9822755 PMC6793310

[17] Watson CJ, Soto-Calderon H, Lydic R, Baghdoyan HA. Pontine reticular formation (PnO) administration of hypocretin-1 increases PnO GABA levels and wakefulness. Sleep. 2008;31(4):453–64. doi: 10.1093/sleep/31.4.453 18457232 PMC2279760

[18] Weber F, Hoang Do JP, Chung S, Beier KT, Bikov M, Saffari Doost M, et al. Regulation of REM and Non-REM Sleep by Periaqueductal GABAergic Neurons. Nat Commun. 2018;9(1):354. doi: 10.1038/s41467-017-02765-w 29367602 PMC5783937

[19] Fraigne JJ, Torontali ZA, Snow MB, Peever JH. REM Sleep at its Core - Circuits, Neurotransmitters, and Pathophysiology. Front Neurol. 2015;6:123. doi: 10.3389/fneur.2015.00123 26074874 PMC4448509

[20] Herrera CG, Cadavieco MC, Jego S, Ponomarenko A, Korotkova T, Adamantidis A. Hypothalamic feedforward inhibition of thalamocortical network controls arousal and consciousness. Nat Neurosci. 2016;19(2):290–8. doi: 10.1038/nn.4209 26691833 PMC5818272

[21] Boucetta S, Cissé Y, Mainville L, Morales M, Jones BE. Discharge profiles across the sleep-waking cycle of identified cholinergic, GABAergic, and glutamatergic neurons in the pontomesencephalic tegmentum of the rat. J Neurosci. 2014;34(13):4708–27. doi: 10.1523/JNEUROSCI.2617-13.2014 24672016 PMC3965793

[22] Brown RE, McKenna JT, Winston S, Basheer R, Yanagawa Y, Thakkar MM, et al. Characterization of GABAergic neurons in rapid-eye-movement sleep controlling regions of the brainstem reticular formation in GAD67-green fluorescent protein knock-in mice. Eur J Neurosci. 2008;27(2):352–63. doi: 10.1111/j.1460-9568.2008.06024.x 18215233 PMC2376819

[23] Cissé Y, Ishibashi M, Jost J, Toossi H, Mainville L, Adamantidis A, et al. Discharge and role of GABA pontomesencephalic neurons in cortical activity and sleep-wake states examined by optogenetics and juxtacellular recordings in mice. J Neurosci. 2020;40(31):5970–89. doi: 10.1523/JNEUROSCI.2875-19.2020 32576622 PMC7392501

[24] An S, Sun H, Wu M, Xie D, Hu S-W, Ding H-L, et al. Medial septum glutamatergic neurons control wakefulness through a septo-hypothalamic circuit. Curr Biol. 2021;31(7):1379-1392.e4. doi: 10.1016/j.cub.2021.01.019 33545041

[25] Xu M, Chung S, Zhang S, Zhong P, Ma C, Chang W-C, et al. Basal forebrain circuit for sleep-wake control. Nat Neurosci. 2015;18(11):1641–7. doi: 10.1038/nn.4143 26457552 PMC5776144

[26] Anaclet C, Pedersen NP, Ferrari LL, Venner A, Bass CE, Arrigoni E, et al. Basal forebrain control of wakefulness and cortical rhythms. Nat Commun. 2015;6:8744. doi: 10.1038/ncomms9744 26524973 PMC4659943

[27] Adamantidis AR, Zhang F, Aravanis AM, Deisseroth K, de Lecea L. Neural substrates of awakening probed with optogenetic control of hypocretin neurons. Nature. 2007;450(7168):420–4. doi: 10.1038/nature06310 17943086 PMC6744371

[28] Fujita A, Bonnavion P, Wilson MH, Mickelsen LE, Bloit J, de Lecea L, et al. Hypothalamic Tuberomammillary Nucleus Neurons: Electrophysiological Diversity and Essential Role in Arousal Stability. J Neurosci. 2017;37(39):9574–92. doi: 10.1523/JNEUROSCI.0580-17.2017 28874450 PMC5618271

[29] Pedersen NP, Ferrari L, Venner A, Wang JL, Abbott SBG, Vujovic N, et al. Supramammillary glutamate neurons are a key node of the arousal system. Nat Commun. 2017;8(1):1405. doi: 10.1038/s41467-017-01004-6 29123082 PMC5680228

[30] Voogd J. Deiters’ nucleus. Its role in cerebellar ideogenesis: the ferdinando rossi memorial lecture. Cerebellum 15, 54–66 (2016). 26054378 10.1007/s12311-015-0681-9 PMC4726724

[31] Murray AJ, Croce K, Belton T, Akay T, Jessell TM. Balance control mediated by vestibular circuits directing limb extension or antagonist muscle co-activation. Cell Rep. 2018;22(5):1325–38. doi: 10.1016/j.celrep.2018.01.009 29386118

[32] Fulton JF, Liddell EGT, Rioch DMCK. The influence of unilateral destruction of the vestibular nuclei upon posture and the knee-jerk. Brain. 1930;53(3):327–43. doi: 10.1093/brain/53.3.327

[33] Chemelli RM, Willie JT, Sinton CM, Elmquist JK, Scammell T, Lee C, et al. Narcolepsy in orexin knockout mice: molecular genetics of sleep regulation. Cell. 1999;98(4):437–51. doi: 10.1016/s0092-8674(00)81973-x 10481909

[34] Peyron C, Faraco J, Rogers W, Ripley B, Overeem S, Charnay Y, et al. A mutation in a case of early onset narcolepsy and a generalized absence of hypocretin peptides in human narcoleptic brains. Nat Med. 2000;6(9):991–7. doi: 10.1038/79690 10973318

[35] Thannickal TC, Moore RY, Nienhuis R, Ramanathan L, Gulyani S, Aldrich M, et al. Reduced number of hypocretin neurons in human narcolepsy. Neuron. 2000;27(3):469–74. doi: 10.1016/s0896-6273(00)00058-1 11055430 PMC8760623

[36] Snow MB, et al. GABA cells in the central nucleus of the amygdala promote cataplexy. J Neurosci. 2017;37:4007–22. 28209737 10.1523/JNEUROSCI.4070-15.2017 PMC6596591

[37] Willie JT, Chemelli RM, Sinton CM, Tokita S, Williams SC, Kisanuki YY, et al. Distinct narcolepsy syndromes in Orexin receptor-2 and Orexin null mice: molecular genetic dissection of Non-REM and REM sleep regulatory processes. Neuron. 2003;38(5):715–30. doi: 10.1016/s0896-6273(03)00330-1 12797957

[38] Scammell TE, Willie JT, Guilleminault C, Siegel JM, International Working Group on Rodent Models of Narcolepsy. A consensus definition of cataplexy in mouse models of narcolepsy. Sleep. 2009;32:111–6. 19189786 PMC2625315

[39] Cox J, Pinto L, Dan Y. Calcium imaging of sleep-wake related neuronal activity in the dorsal pons. Nat Commun. 2016;7:10763. doi: 10.1038/ncomms10763 26911837 PMC4773416

[40] McCarley RW, Hobson JA. Neuronal excitability modulation over the sleep cycle: a structural and mathematical model. Science. 1975;189:58–60. 1135627 10.1126/science.1135627

[41] McGinty D, Szymusiak R. The sleep-wake switch: a neuronal alarm clock. Nat Med. 2000;6(5):510–1. doi: 10.1038/74988 10802704

[42] Sapin E, Lapray D, Bérod A, Goutagny R, Léger L, Ravassard P, et al. Localization of the brainstem GABAergic neurons controlling paradoxical (REM) sleep. PLoS One. 2009;4(1):e4272. doi: 10.1371/journal.pone.0004272 19169414 PMC2629845

[43] Liu D, Dan Y. A motor theory of sleep-wake control: arousal-action circuit. Annu Rev Neurosci. 2019;42:27–46. doi: 10.1146/annurev-neuro-080317-061813 30699051

[44] Alam MA, Kumar S, McGinty D, Alam MN, Szymusiak R. Neuronal activity in the preoptic hypothalamus during sleep deprivation and recovery sleep. J Neurophysiol. 2014;111(2):287–99. doi: 10.1152/jn.00504.2013 24174649 PMC3921380

[45] Moore RY. Suprachiasmatic nucleus in sleep-wake regulation. Sleep Med. 2007;8 Suppl 3:27–33. doi: 10.1016/j.sleep.2007.10.003 18032104

[46] Kim T, Thankachan S, McKenna JT, McNally JM, Yang C, Choi JH, et al. Cortically projecting basal forebrain parvalbumin neurons regulate cortical gamma band oscillations. Proc Natl Acad Sci U S A. 2015;112(11):3535–40. doi: 10.1073/pnas.1413625112 25733878 PMC4371918

[47] Kashiwagi M, Beck G, Kanuka M, Arai Y, Tanaka K, Tatsuzawa C, et al. A pontine-medullary loop crucial for REM sleep and its deficit in Parkinson’s disease. Cell. 2024;187(22):6272-6289.e21. doi: 10.1016/j.cell.2024.08.046 39303715

[48] Everitt BJ, Robbins TW. Central cholinergic systems and cognition. Annu Rev Psychol. 1997;48:649–84. doi: 10.1146/annurev.psych.48.1.649 9046571

[49] Stuber GD, Wise RA. Lateral hypothalamic circuits for feeding and reward. Nat Neurosci. 2016;19(2):198–205. doi: 10.1038/nn.4220 26814589 PMC4927193

[50] Lomax P, Green MD. Histaminergic neurons in the hypothalamic thermoregulatory pathways. Fed Proc. 1981;40(13):2741–5. 6117483

[51] Blandina P, Provensi G, Munari L, Passani MB. Histamine neurons in the tuberomamillary nucleus: a whole center or distinct subpopulations? Front Syst Neurosci. 2012;6. 10.3389/fnsys.2012.00033 PMC3343474 22586376

[52] Zhong P, Zhang Z, Barger Z, Ma C, Liu D, Ding X, et al. Control of non-REM sleep by midbrain neurotensinergic neurons. Neuron. 2019;104(4):795-809.e6. doi: 10.1016/j.neuron.2019.08.026 31582313

[53] Vetrivelan R, Kong D, Ferrari LL, Arrigoni E, Madara JC, Bandaru SS, et al. Melanin-concentrating hormone neurons specifically promote rapid eye movement sleep in mice. Neuroscience. 2016;336:102–13. doi: 10.1016/j.neuroscience.2016.08.046 27595887 PMC5056843

[54] Boissard R, Fort P, Gervasoni D, Barbagli B, Luppi P-H. Localization of the GABAergic and non-GABAergic neurons projecting to the sublaterodorsal nucleus and potentially gating paradoxical sleep onset. Eur J Neurosci. 2003;18(6):1627–39. doi: 10.1046/j.1460-9568.2003.02861.x 14511341

[55] Clément O, Sapin E, Bérod A, Fort P, Luppi P-H. Evidence that neurons of the sublaterodorsal tegmental nucleus triggering paradoxical (REM) sleep are glutamatergic. Sleep. 2011;34(4):419–23. doi: 10.1093/sleep/34.4.419 21461384 PMC3064553

[56] Fraigne JJ, Adamantidis AR, Peever JH. Optogenetic investigation of rapid eye movement (REM) sleep circuitry. Sleep. 2014;37:A21.

[57] Fromherz S, Mignot E. Overview of human narcolepsy. In: Nishino S, Sakurai T, editors. The orexin/hypocretin system. Totowa, NJ: Humana Press; 2006. p. 221–31. doi: 10.1385/1-59259-950-8:221

[58] Lin L, Faraco J, Li R, Kadotani H, Rogers W, Lin X, et al. The sleep disorder canine narcolepsy is caused by a mutation in the hypocretin (orexin) receptor 2 gene. Cell. 1999;98(3):365–76. doi: 10.1016/s0092-8674(00)81965-0 10458611

[59] Feng H, Wen S-Y, Qiao Q-C, Pang Y-J, Wang S-Y, Li H-Y, et al. Orexin signaling modulates synchronized excitation in the sublaterodorsal tegmental nucleus to stabilize REM sleep. Nat Commun. 2020;11(1):3661. doi: 10.1038/s41467-020-17401-3 32694504 PMC7374574

[60] Date Y, Ueta Y, Yamashita H, Yamaguchi H, Matsukura S, Kangawa K, et al. Orexins, orexigenic hypothalamic peptides, interact with autonomic, neuroendocrine and neuroregulatory systems. Proc Natl Acad Sci U S A. 1999;96(2):748–53. doi: 10.1073/pnas.96.2.748 9892705 PMC15208

[61] Sachidanandan D, Reddy HP, Mani A, Hyde GJ, Bera AK. The neuropeptide orexin-a inhibits the GABAA receptor by PKC and Ca2+/CaMKII-dependent phosphorylation of its β1 subunit. J Mol Neurosci. 2017;61(4):459–67. doi: 10.1007/s12031-017-0886-0 28105535

[62] Schmidt MH, Bassetti CLA. Gender differences in narcolepsy: what are recent findings telling us? Sleep. 2022;45(12):zsac126. doi: 10.1093/sleep/zsac126 35640640

[63] Tsuchiya R, Yoshiki F, Kudo Y, Morita M. Cell type-selective expression of green fluorescent protein and the calcium indicating protein, yellow cameleon, in rat cortical primary cultures. Brain Res. 2002;956(2):221–9. doi: 10.1016/s0006-8993(02)03518-7 12445689

[64] Jia X, Kohn A. Gamma rhythms in the brain. PLoS Biol. 2011;9(4):e1001045. doi: 10.1371/journal.pbio.1001045 21556334 PMC3084194

