# Supplementary figures and images for: GABA neurons in the sublaterodorsal tegmental nucleus suppress wakefulness in healthy and narcoleptic mice
Source: PLoS Biol. 2026 Jul 8;24(7):e3003303. doi: 10.1371/journal.pbio.3003303 (PMC13345269; doi:10.1371/journal.pbio.3003303)

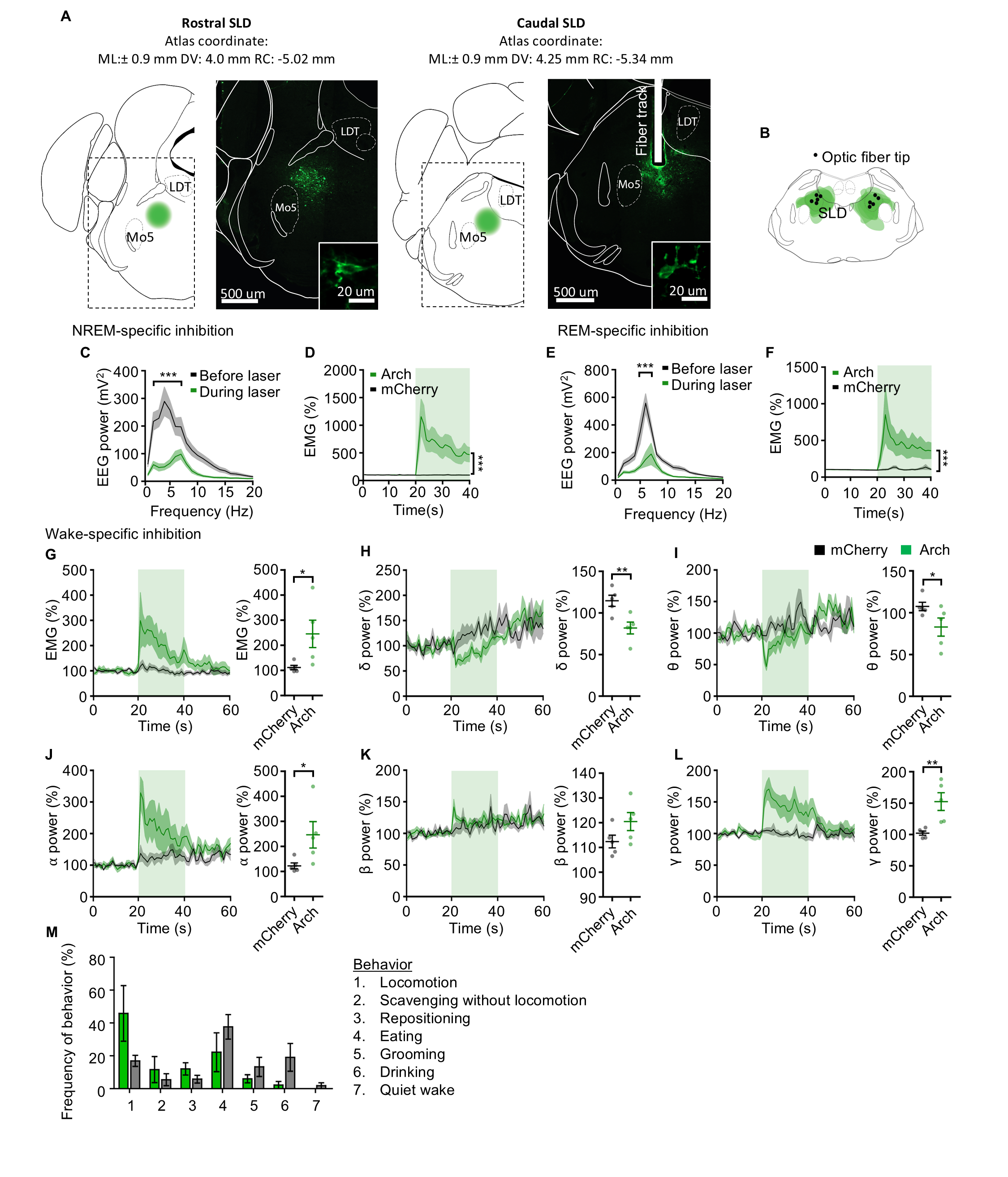

Supplement: S1 Fig — (A) Schematic and Microscope image of the rostral and caudal SLD (coronal section) showing the area of viral expression (Arch-GFP, green) and location of the implanted optic fiber above the SLD (white line). (B) A schematic showing the area of viral expression and location of implanted optic fiber tips in the SLD (n = 5). (C and E) Power spectral density of EEG before and during NREM-specific and REM-specific inhibition (n = 5; Two-way RM ANOVA with Bonferroni post-test). (D and F) EMG activity before and during NREM-specific and REM-specific inhibition (mCherry n = 5 and Arch n = 5; Two-way ANOVA with Bonferroni post-test). (G) LEFT: EMG activity before, during and after 20 s wake-specific inhibition (mCherry n = 5 and Arch n = 5). RIGHT: Mean EMG activity during the 20 s inhibition (mCherry n = 5 and Arch n = 5; unpaired t test). (H–L) LEFT: δ, θ, α, β, and γ EEG activity before, during and after 20 s wake-specific inhibition (mCherry n = 5 and Arch n = 5). RIGHT: Mean δ, θ, α, β, and γ EEG activity during the 20 s inhibition (mCherry n = 5 and Arch n = 5; unpaired t test). (M) Animal behavior during wake-specific inhibition (mCherry n = 5, 53 trials and Arch n = 5, 48 trials). EEG bands: δ (delta, 0.5–4 Hz), θ (theta, 4–8 Hz), α (alpha, 8–12 Hz), β (beta, 12–30 Hz), and γ (gamma, 30–100 Hz). Green patches indicate time of the inhibition (for eArch3.0) or sham laser inhibition (for mCherry). All error bars and shades represent ±s.e.m. * p < 0.05, ** p < 0.01, *** p < 0.001 indicate significant differences. Abbreviations: medio-lateral (ML), dorso-ventral (DV), rostro-caudal (RC), superior cerebellar peduncle (scp), trigeminal motor nucleus (Mo5), and 4th ventricle (4V). Atlas coordinates and coronal brain schematics are from Allen Brain Atlas version 3rd edition. The data underlying this Figure can be found in S5 Data. (TIF) [file pbio.3003303.s001.tif]

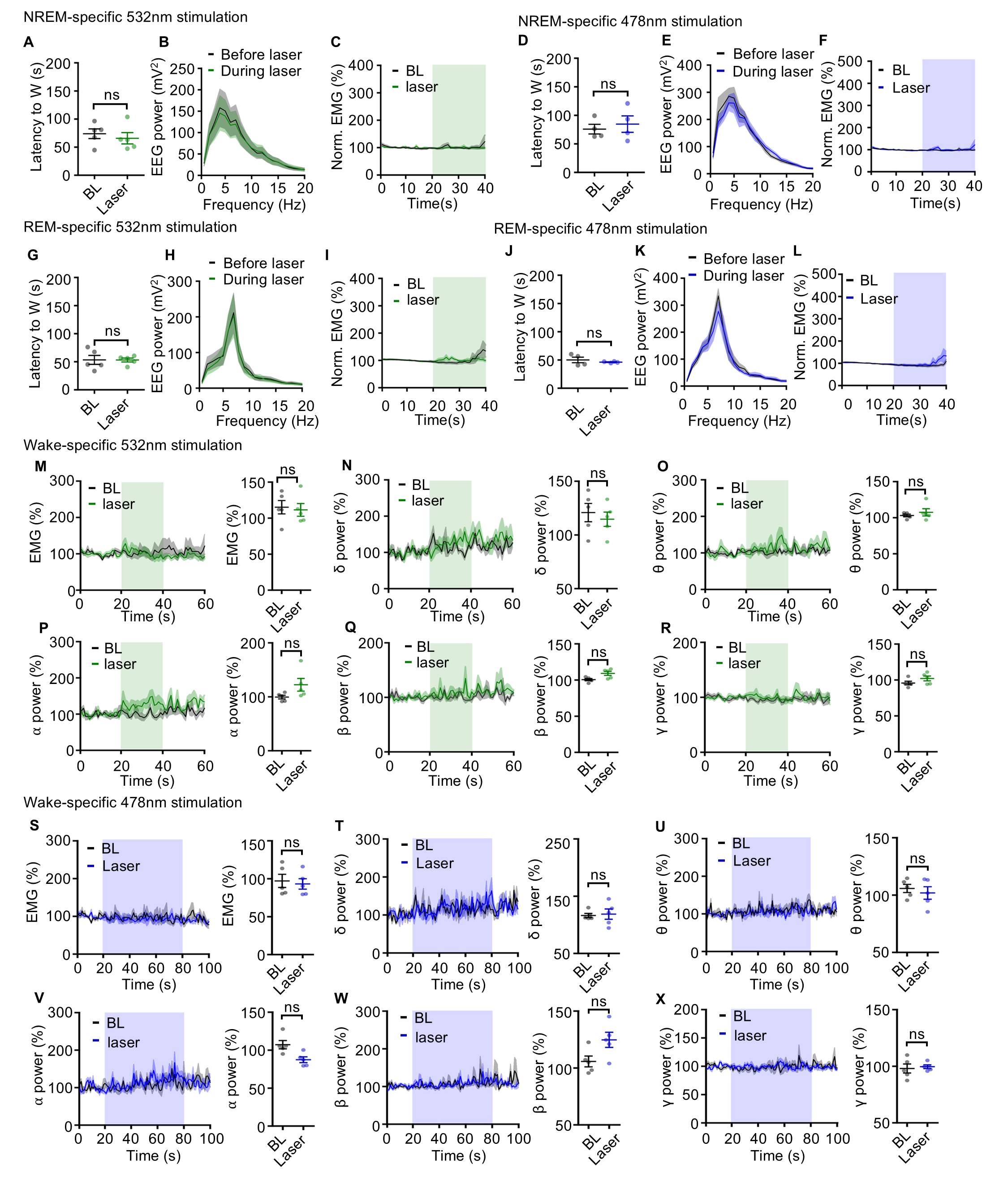

Supplement: S2 Fig — (A) Average latency to wake from NREM sleep during baseline and 532 nm laser stimulation (continuous; n = 5; paired t test) (B) Power spectral density of EEG before and during 532 nm laser stimulation from NREM sleep (n = 5; Two-way RM ANOVA). (C) EMG activity across NREM sleep during baseline and 532 nm laser stimulation (n = 5; Two-way RM ANOVA). (D) Average latency to wake from NREM sleep during baseline and 478 nm laser stimulation (n = 4; paired t test) (E) Power spectral density of EEG before and during 478 nm laser stimulation from NREM sleep (n = 4; Two-way RM ANOVA). (F) EMG activity across NREM sleep during baseline and 478 nm laser stimulation (n = 4; Two-way RM ANOVA). (G) Average latency to wake from REM sleep during baseline and 532 nm laser stimulation (n = 5; paired t test). (H) Power spectral density of EEG before and during 532 nm laser stimulation from REM sleep (n = 5; Two-way RM ANOVA). (I) EMG activity across REM sleep during baseline and 532 nm laser stimulation (n = 5; Two-way RM ANOVA). (J) Average latency to wake from REM sleep during baseline and 478 nm laser stimulation (n = 4; paired t test). (K) Power spectral density of EEG before and during 478 nm laser stimulation from REM sleep (n = 4; Two-way RM ANOVA). (L) EMG activity across REM sleep during baseline and 478 nm laser stimulation (n = 4; Two-way RM ANOVA). (M) LEFT: EMG activity before, during and after 532 nm laser stimulation during wakefulness compared to baseline wakefulness (n = 5; Two-way RM ANOVA). RIGHT: Mean EMG activity during the 532 nm laser stimulation compared to baseline wakefulness (n = 5; paired t test). (N–R) LEFT: δ, θ, α, β, and γ EEG activity before, during, and after 532 nm laser stimulation from wakefulness compared to baseline wakefulness (n = 5). RIGHT: Mean δ, θ, α, β, and γ EEG activity during the 532 nm laser stimulation compared to baseline wakefulness (n = 5; paired t test). (S) LEFT: EMG activity before, during and after 478 nm laser stimulation duri [file pbio.3003303.s002.tif]

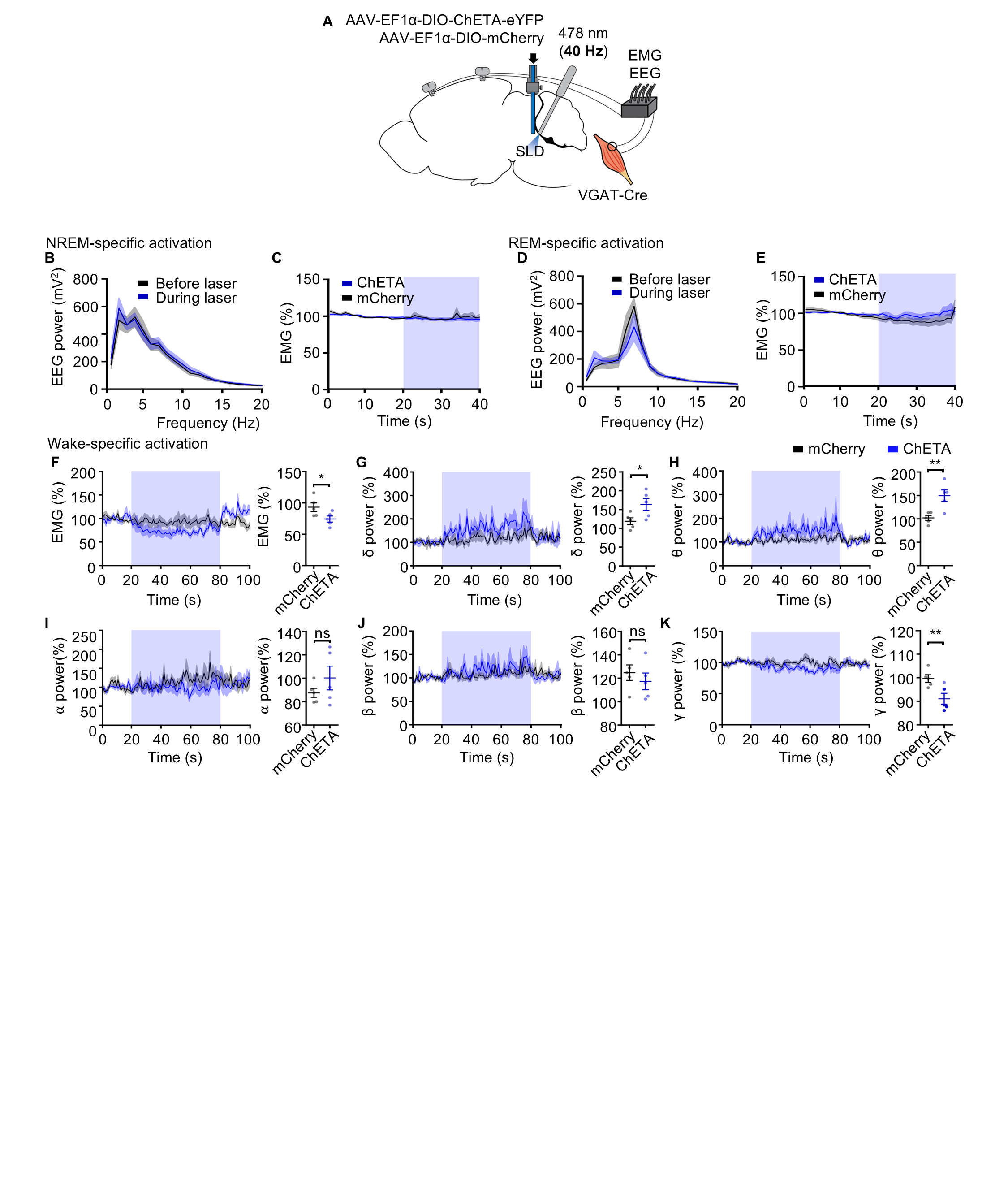

Supplement: S3 Fig — (A) A schematic showing optogenetic activation (478 nm, 40 Hz, 5 ms pulses) of SLDGABA neurons coupled with EEG and EMG recordings. (B) Power spectral density of EEG before and during NREM-specific activation (n = 5; Two-way RM ANOVA). (C) EMG activity before and during NREM-specific activation (mCherry n = 4 and ChETA n = 5; Two-way ANOVA with Bonferroni post-test). (D) Power spectral density of EEG before and during REM-specific activation (n = 5; Two-way RM ANOVA with Bonferroni post-test). (E) EMG activity before and during REM-specific activation (mCherry n = 4 and ChETA n = 5; Two-way ANOVA). (F) LEFT: EMG activity before, during, and after wake-specific activation (mCherry n = 4 and ChETA n = 5). RIGHT: Mean EMG activity during the activation (mCherry n = 4 and ChETA n = 5; unpaired t test). (G–K) LEFT: δ, θ, α, β, and γ EEG activity before, during, and after wake-specific activation (mCherry n = 4 and ChETA n = 5). RIGHT: Mean δ, θ, α, β, and γ activity during the activation (mCherry n = 4 and ChETA n = 5; unpaired t test). EEG bands: δ (delta, 0.5–4 Hz), θ (theta, 4–8 Hz), α (alpha, 8–12 Hz), β (beta, 12–30 Hz), and γ (gamma, 30–100 Hz). Blue patches indicate time of laser stimulation. All error bars and shades represent ±s.e.m. * p < 0.05, ** p < 0.01 indicate significant differences. The data underlying this Figure can be found in S7 Data. (TIF) [file pbio.3003303.s003.tif]

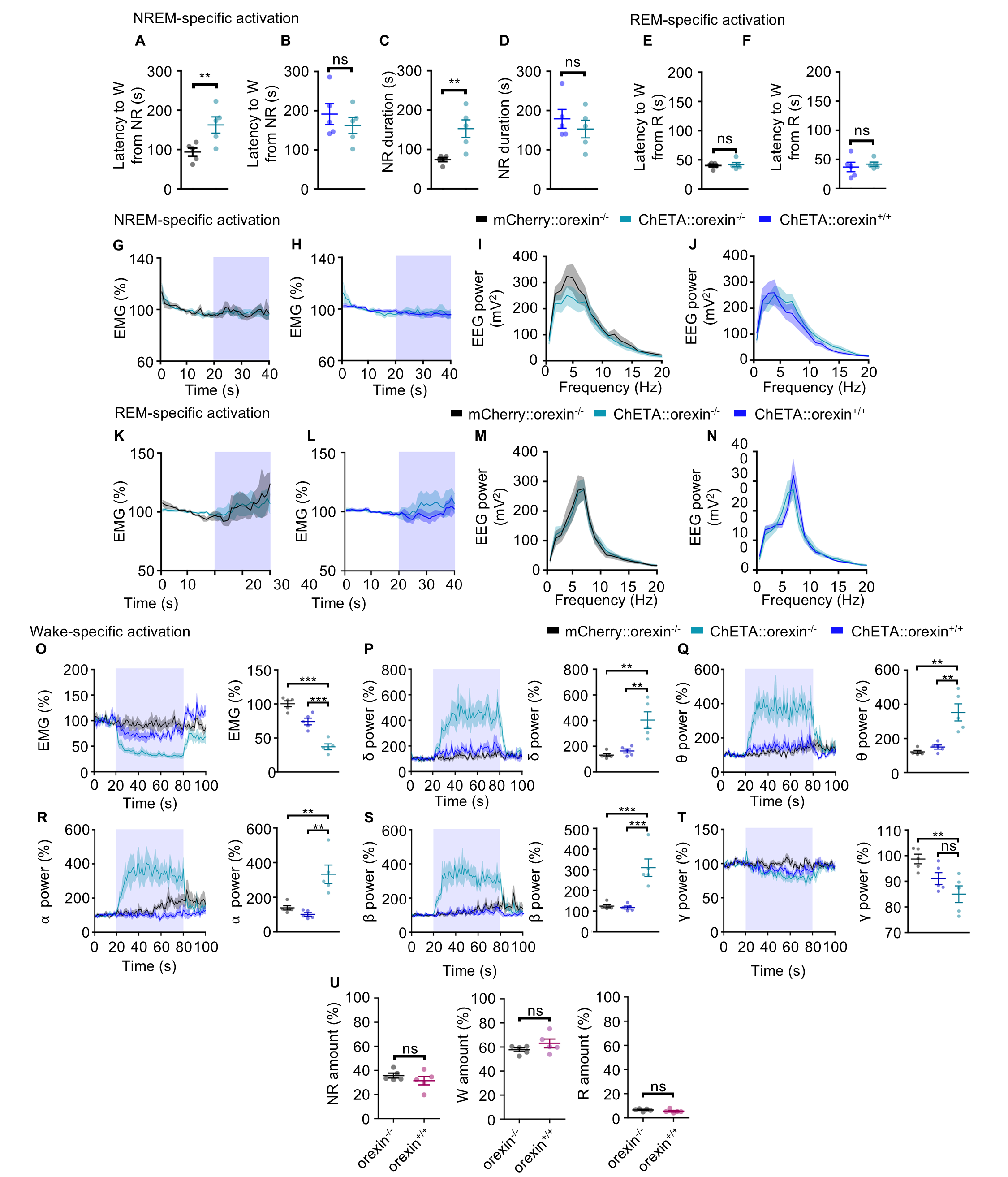

Supplement: S4 Fig — (A and B) Latency to wake from NREM sleep upon activation of SLDGABA neurons (478 nm, 40 Hz; mCherry::orexin−/− n = 5, ChETA::orexin−/− n = 5, and ChETA::orexin+/+ n = 5; unpaired t test). (C and D) Duration of NREM sleep upon activation (mCherry::orexin−/− n = 5, ChETA::orexin−/− n = 5, and ChETA::orexin+/+ n = 5; unpaired t test). (E and F) Latency to wake from REM sleep upon activation (478 nm, 40 Hz; mCherry::orexin−/− n = 5, ChETA::orexin−/− n = 5, and ChETA::orexin+/+ n = 5; unpaired t test). (G and H) EMG activity before and during NREM-specific activation (mCherry::orexin−/− n = 5, ChETA::orexin−/− n = 5, and ChETA::orexin+/+ n = 5; Two-way ANOVA). (I and J) Power spectral density of EEG during NREM-specific activation (mCherry::orexin−/− n = 5, ChETA::orexin−/− n = 5, and ChETA::orexin+/+ n = 5; Two-way ANOVA). (K and L) EMG activity before and during REM-specific activation (mCherry::orexin−/− n = 5, ChETA::orexin−/− n = 5, and ChETA::orexin+/+ n = 5; Two-way ANOVA). (M and N) Power spectral density of EEG during REM-specific activation (mCherry::orexin−/− n = 5, ChETA::orexin−/− n = 5, and ChETA::orexin+/+ n = 5; Two-way ANOVA). (O) LEFT: EMG activity before, during, and after 60 s wake-specific activation (mCherry::orexin−/− n = 5, ChETA::orexin+/+ n = 5, and ChETA::orexin−/− n = 5). RIGHT: Mean EMG activity during the activation (mCherry::orexin−/− n = 5, ChETA::orexin+/+ n = 5, and ChETA::orexin−/− n = 5; unpaired t test). (P–T) LEFT: δ, θ, α, β, and γ EEG activity before, during and after 60 s wake-specific activation (mCherry::orexin−/− n = 5, ChETA::orexin+/+ n = 5, and ChETA::orexin−/− n = 5). RIGHT: Mean δ, θ, α, β, and γ EEG activity during the activation (mCherry::orexin−/− n = 5, ChETA::orexin+/+ n = 5, and ChETA::orexin−/− n = 5; unpaired t test). (U) The amount of NREM, wake, and REM sleep is comparable between narcoleptic and healthy animals during 20:00–23:00 (orexin−/− n = 5, orexin+/+ n = 5). EEG bands: δ (delta, 0.5–4 Hz), θ (theta, 4–8 [file pbio.3003303.s004.tif]

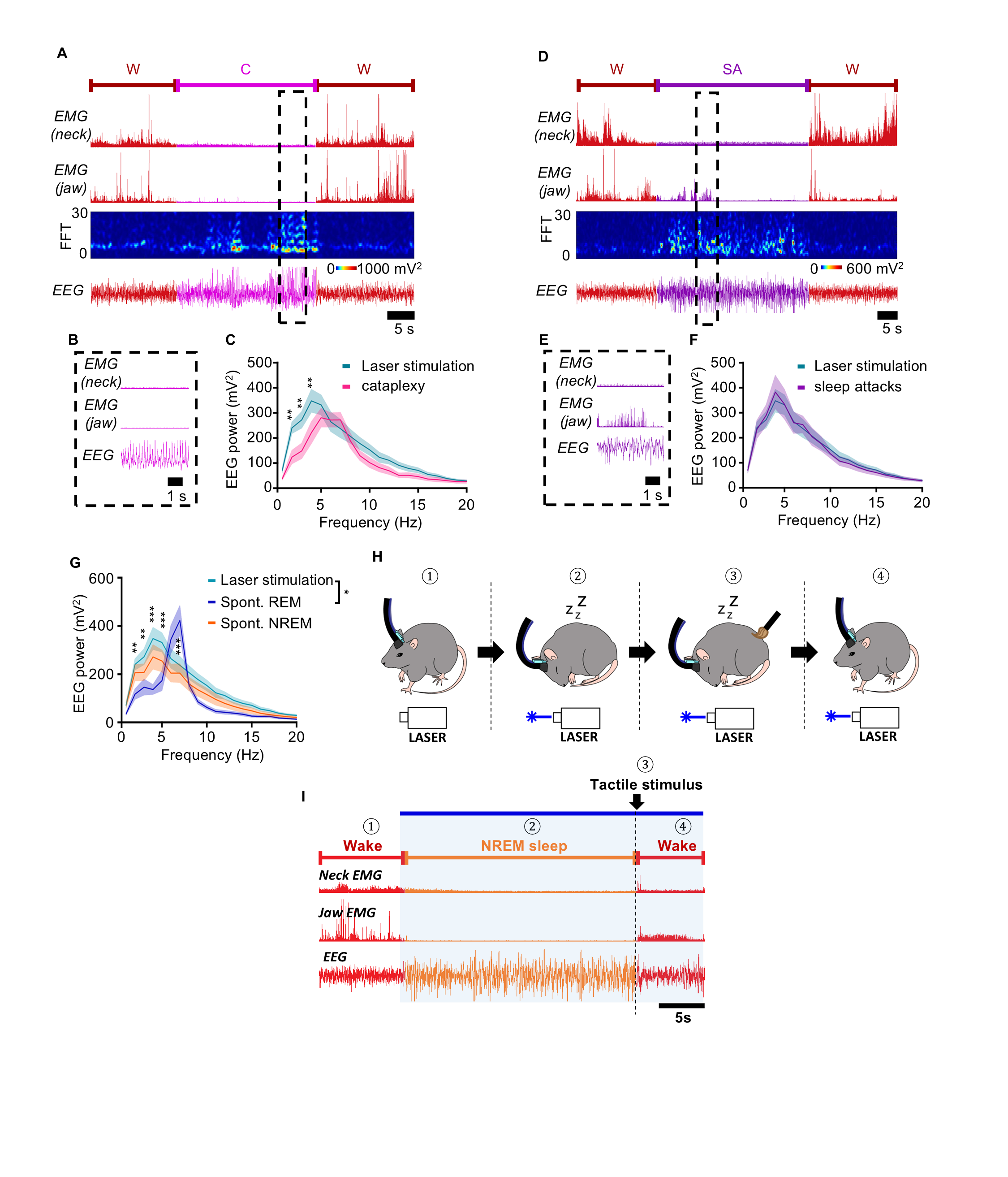

Supplement: S5 Fig — (A) Example polysomnogram during cataplexy. Shown are EMG amplitude, EEG spectrogram, and EEG raw traces. (B) Magnified polysomnogram during the time window shown in dotted box in (A). (C) Power spectral density of EEG during activation of SLDGABA neurons compared to spontaneous cataplexy attack (n = 5; Two-way RM ANOVA with Bonferroni post-test) (D) Example polysomnogram during sleep attacks. (E) Magnified polysomnogram during the time window shown in dotted box in (D). (F) Power spectral density of EEG during activation of SLDGABA neurons compared to spontaneous sleep attacks (n = 5; Two-way RM ANOVA). (G) Power spectral density of EEG during activation of SLDGABA neurons compared to spontaneous NREM and REM sleep (n = 5; Two-way RM ANOVA with Bonferroni post-test). (H) A schematic summarizing the observation made from delivering tactile stimuli (i.e., touching a mouse with a paintbrush) during the activation of SLDGABA neurons in orexin−/− mice. ① animal is in baseline wake state with no activation, ② activation of SLDGABA neurons induces transition into NREM sleep, ③ during the activation, physical stimulus is delivered to the animal, and ④ despite the sustained activation, the animal responded to the physical stimuli by waking up. (I) Example polysomnogram showing orexin−/− mice that are undergoing the procedure depicted from (H) (n = 5). Shown are EMG amplitude and EEG raw traces. All error bars and shades represent ±s.e.m. ** p < 0.01 *** p < 0.001 indicate significant differences. (TIF) [file pbio.3003303.s005.tif]

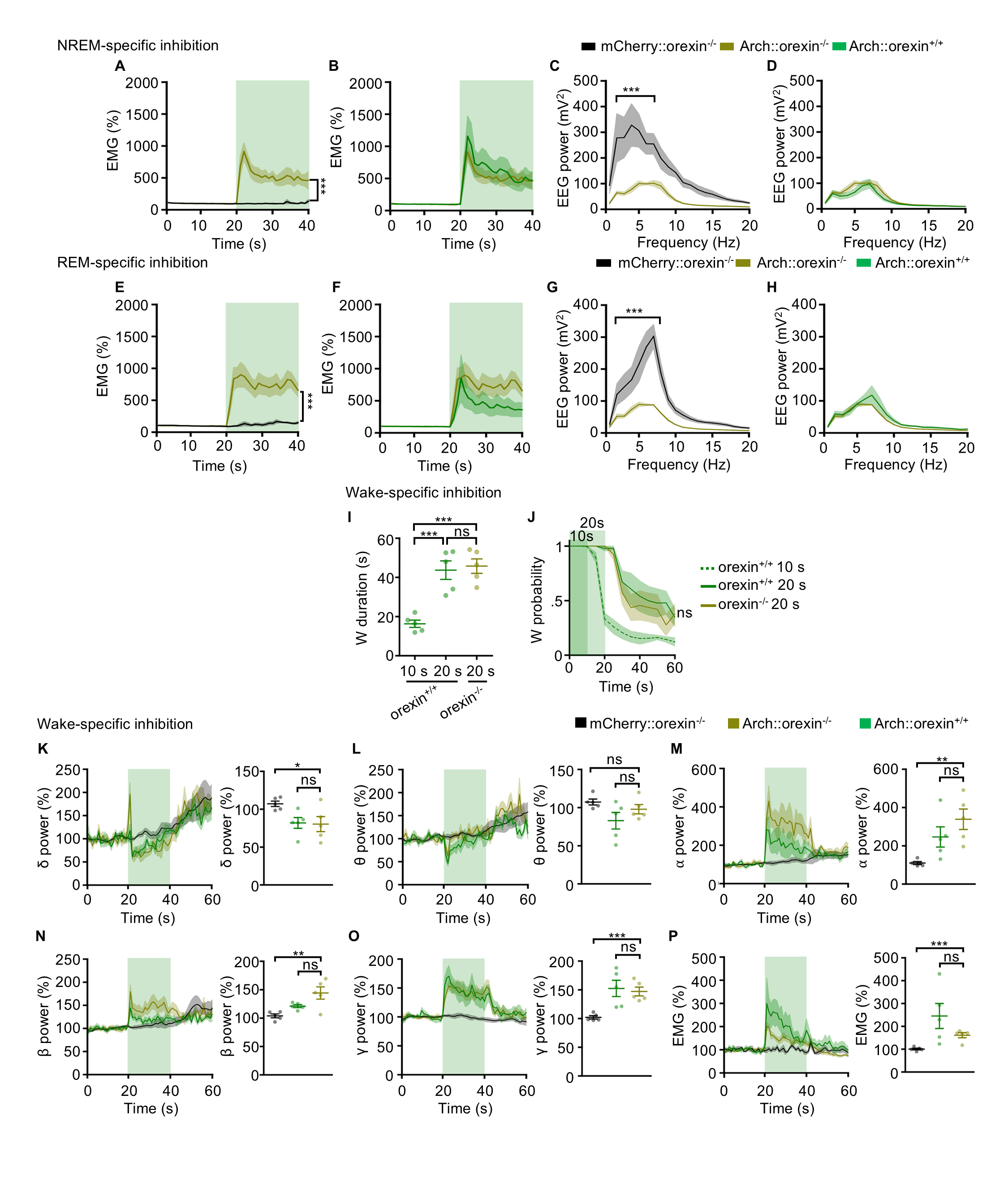

Supplement: S6 Fig — (A and B) EMG activity before and during NREM-specific inhibition (532 nm; mCherry::orexin−/− n = 5, Arch::orexin−/− n = 5, and Arch::orexin+/+ n = 5; Two-way ANOVA with Bonferroni post-test). (C and D) Power spectral density of EEG during NREM-specific inhibition (mCherry::orexin−/− n = 5, Arch::orexin−/− n = 5, and Arch::orexin+/+ n = 5; Two-way ANOVA). (E and F) EMG activity before and during REM-specific inhibition (mCherry::orexin−/− n = 5, Arch::orexin−/− n = 5, and Arch::orexin+/+ n = 5; Two-way ANOVA). (G and H) Power spectral density of EEG during REM-specific inhibition (mCherry::orexin−/− n = 5, Arch::orexin−/− n = 5, and Arch::orexin+/+ n = 5; Two-way ANOVA with Bonferroni post-test). (I) Duration of wakefulness induced by 10 and 20 s inhibition (orexin+/+ n = 5 and orexin−/− n = 5; one-way ANOVA with Tukey’s Multiple Comparison Test). (J) Probability of wakefulness in response to 10 and 20 s inhibition during wakefulness (orexin+/+ n = 5 and orexin−/− n = 5; two-way ANOVA). (K–O) LEFT: δ, θ, α, β, and γ EEG activity before, during, and after 20 s wake-specific inhibition (mCherry::orexin−/− n = 5, Arch::orexin−/− n = 5, and Arch::orexin+/+ n = 5). RIGHT: Mean δ, θ, α, β, and γ EEG activity during the 20 s inhibition (mCherry::orexin−/− n = 5, Arch::orexin−/− n = 5, and Arch::orexin+/+ n = 5; unpaired t test). (P) LEFT: EMG activity before, during, and after 20 s wake-specific inhibition (mCherry::orexin−/− n = 5, Arch::orexin−/− n = 5, and Arch::orexin+/+ n = 5). RIGHT: Mean EMG activity during the 20 s inhibition (mCherry::orexin−/− n = 5, Arch::orexin−/− n = 5, and Arch::orexin+/+ n = 5; unpaired t test). EEG power bands: δ (delta, 0.5–4 Hz), Lθ (theta, 4–8 Hz), Hθ (alpha, 8–12 Hz), β (beta, 12–30 Hz), and γ (gamma, 30–100 Hz). Green patches indicate time of laser stimulation. All error bars and shades represent ±s.e.m. All error bars and shades represent ±s.e.m. * p < 0.05, ** p < 0.01, *** p < 0.001 indicate significant differences. The data underly [file pbio.3003303.s006.tif]
