# Supplementary material for: GABA neurons in the sublaterodorsal tegmental nucleus suppress wakefulness in healthy and narcoleptic mice
Source: PLoS Biol. 2026 Jul 8;24(7):e3003303. doi: 10.1371/journal.pbio.3003303 (PMC13345269; doi:10.1371/journal.pbio.3003303)
Supplement: Attachment — Submitted filename: Response to the reviewer-PLOSBiology-Mar-2026-FINAL.docx [file pbio.3003303.s020.docx]

# Reviewer #1

Akihiro Yamanaka (note, reviewer 1 has signed this review): This study focuses on the SLDGABA neurons in the regulation of sleep/wakefulness. Through axonal tracing, the authors demonstrated that SLDGABA projects to multiple wake-promoting brain regions, providing an anatomical basis for its function. The most novel aspect of this work is the investigation of the role of SLDGABA neurons in regulating sleep attacks in narcolepsy. In ORX-/- mice, activation of SLDGABA induces sleep attacks, whereas inhibition of SLDGABA causes immediate arousal in mice undergoing a sleep attack. Moreover, silencing of SLDGABA also terminates cataplexy, restoring motor activity. The finding that SLDGABA is both sufficient and necessary for the regulation of sleep attacks highlights its potential as a promising therapeutic target for narcolepsy. This reviewer suggests the following points to improve the manuscripts.

We thank Dr. Yamanaka for his review and useful comments.

Major comments

1)      Activation of SLDGABA immediately induced NREM sleep in orexin-/- mice is interesting. However, it is not clear whether this is because sleep pressure is higher in these mice or the orexin signal affects the base activity of these neurons. To make it clear, please test whether the activation of SLDGABA in the mild sleep deprivation condition in orexin+/+ mice shortens the latency to NREM sleep or not.

**Response:** Thank you for your comment. A previous study by Mochizuki et al. (2004) demonstrated that the fragmented wakefulness observed in orexin-deficient mice is not due to abnormal sleep homeostasis and increased sleep pressure^1^. Instead, the fragmented behaviour of orexin^-/-^ mice is best described as behavioural-state instability, with low thresholds for transitions between states. We have conducted additional analysis to show that the orexin^-/-^ animals exhibit comparable amounts of sleep and wake amounts to wildtype animals during the time of GABA SLD neuron activation (Figure 1), suggesting that both groups of mice have similar sleep pressure. We have discussed this point further in the discussion of the manuscript.

2)      Figure 1, Open-loop inhibition, it is clear that light off increased the EMG signal as well. Is this because rebound firing induces wakefulness?

**Response**: We apologize for the confusion. Activation of SLD^GABA^ neurons and not inhibition decreases EMG activity. As noted by the reviewer, termination of the activation induces a rebound of EMG activity (a feature of wakefulness). This is evident in both the orexin^+/+^ and orexin^-/-^ mice (Manuscript Figure 2J and 4M). We speculate that terminating the activation of SLD^GABA^ neurons induces rebound firing of downstream wake-promoting neurons, thereby inducing a rebound of wakefulness and wake features (i.e., increase in brain activity and muscle activity). We have clarified this in the discussion.

3)      Figure 1, open-loop stimulation and closed-loop stimulation in the Figure is confusing since it is an inhibition, and Figure 1H, 1J and 1L indicate inhibition.

**Response:** Thank you for your valuable feedback. We revised the figure and figure caption for better clarity. We have revised the manuscript to clearly indicate closed-loop (i.e., state-specific) or open-loop (i.e., state-independent) inhibition or activation, and we refrained from using the term “stimulation” (which can be inhibition/activation depending on the context) and used “inhibition” or “activation” in its place.

4)      Figure 1L, inhibition during wake induced theta activity. Is this wakefulness comparable to the normal wakefulness?

**Response:** Thank you for your question. Upon further investigation of our data, we found that inhibition of GABA SLD neurons during wakefulness typically induced more active behaviour, predominantly locomotion (eArch3.0:eYFP; 43.8%:19.6%), which is typically coupled with an increase in theta activity. This has been included in the result section for SLD^GABA^ neuron inhibition in healthy mice.

5)      Please add the success rate of closed-loop illumination in each state. This means how accurately the algorithm predicts the state of the animal.

**Response:** Our method yielded near-perfect accuracy in state detection and timing of light delivery. The success rate of the closed-loop illumination was 99.16%. Upon completion of closed-loop experiments, all inaccurate trials caused by human or system error have been removed from the analysis. We have clarified our approach in the method section.

6)      Zoltan A. Torontali, John Peever et al., Curr Biol 2019 showed that inhibition of SLD neurons inhibited cataplexy in orexin-/- mice. Similarly, does inhibition of SLD GABA inhibit the number of cataplexy episodes?

**Response:** Thank you for your insightful question. Our optogenetic inhibition of SLD^GABA^ neurons produced immediate termination of ongoing cataplexy and sleep attacks, restoring wakefulness. Optogenetics is a method that excels at probing short-term, fast-acting neural change. However, using optogenetics for prolonged manipulation poses a risk of photodamage. This is why we did not use optogenetics to address whether prolonged and sustained inhibition of SLD^GABA^ neurons could reduce the number of cataplexy and sleep attacks.

In contrast, our previous work (Torontali et al., 2019) used a chemogenetic approach, which is more suitable for long-lasting silencing of neuronal firing, to test the involvement of SLD glutamate (SLD^GLU^) neurons in cataplexy regulation. We agree with the reviewer that the question of the role of SLD^GABA^ neurons in the occurrence of these symptoms is of interest. We propose that a future study using a chemogenetic approach would answer this question, and we have integrated this point in our discussion.

7)      Figure 3 showed that SLD GABA neurons densely innervated many brain areas. However, the authors are just focusing on orexin neurons in the LH, which is not the densest innervation. It's better to add some descriptions of the reason to focus on this pathway.

**Response:** Thank you for your comment. We have now clarified in our manuscript that our study does not propose the lateral hypothalamus (LH) as the only cardinal wake center through which SLD^GABA^ neurons suppress wakefulness. In fact, our findings show that activation of SLD^GABA^ neurons in the absence of LH orexin produces an even stronger suppression of wakefulness.

We have clarified in our manuscript that the tracing experiment for SLD^GABA^ neural projections (Figure 3) was conducted as an exploratory analysis, and this finding did not contribute to our decision to study the SLD in orexin^-/-^ animals. Our rationale for testing the role of SLD^GABA^ neurons in orexin^-/-^ animals stems from longstanding hypotheses that the loss of orexin signalling —directly or indirectly affecting the SLD— underlies the pathophysiology of narcolepsy, and this information is highlighted in the first paragraph of the orexin^-/-^ activation result section. In addition, we agree that the inclusion of a schematic summary figure (as suggested in Comment #9) will further clarify this point.

8)      Kashiwagi et al., Cell 2024, Figure 4 have already indicated that activation of GABAergic projection from SLD to BF induced NREM sleep. This study confirms this. The author should mention this.

**Response:** Thank you for your comment. We agree that the Kashiwagi et al., paper is a critical paper that further strengthens and supports our findings. We revised the discussion section to include this study.

9)      This reviewer suggests that the author include a schematic drawing of the summary. **Response:** A summary figure has been generated to ease the comprehension of the study results. This figure is added as **Figure 6.**

Minor comments:

1)      All Figures and text, add a space between the number and the unit.

**Response:** All figures and texts have been revised to have a space between numbers and units.

2)      Figure 1O, 1P and 1Q. Please standardize the way of writing, Arch, and mCherry.

**Response:** Revisions have been made to standardize “Arch” and “mCherry”.

3)      Figure 4C, This is closed-loop stimulation, probably not the inhibition.

**Response:** To prevent confusion with the term “stimulation”, we revised the manuscript to specifically indicate inhibition or activation for each experiment. In addition, instead of using the term “closed-loop” for all figures, we used the term: Wake-specific, NREM-specific or REM-specific activation or inhibition. We have clarified these terms to avoid any confusion.

4)      Figure 1G should indicate which color indicates which state.

**Response**: The colors in Figure 1G have been labelled

# Reviewer #2

In this study, Lee and colleagues explored the role of GABAergic neurons within the sublaterodorsal tegmental nucleus (SLD) in sleep-wake regulation, in wild-type and narcoleptic mice. This is novel and timely. SLD glutamatergic neurons play a key role in arousal state control, especially REM sleep. By contrast, whether SLD GABA neurons modulate sleep-wake states remains unknown. The experiments, including optogenetic manipulation and anterograde viral tracing mapping of efferent projections, were rigorously conducted and well controlled. The data are compelling and clearly presented. The main findings are that SLD GABAergic neurons suppress wakefulness in wild type mice. In the narcolepsy mouse model, their activation during wakefulness produced rapid NREM onset ("sleep attacks" characteristic of narcolepsy), but it did not trigger or worsen cataplexy. Optical silencing of these neurons reduced the sleep attacks and cataplexy. Thus, these results suggest a role for SLD GABAergic neurons in the regulation of sleep attacks or excessive sleepiness in narcolepsy. Please find a few minor comments below:

We thank the reviewer for their supportive comments about our work.

1) Typo in Intro, line 20: "systems"

**Response:** We have corrected the typo.

2)      For more clarity, I suggest that "wild-type" mice is defined the first time it is used. I was initially confused by reading wild-type when referring to non-orexin deficient, transgenic Vgat-Cre mice.

**Response**: We understand the confusion with the term “wild-type”. Given that the animal with normal orexin signalling (i.e., orexin^+/+^) will be a comparison against a disease model (i.e., narcoleptic mice), we have edited the manuscript to refer to *healthy* (i.e., orexin^+/+^) mice and *narcoleptic* (i.e., orexin^-/-^) mice and defined these terms at their first mention to avoid ambiguity.

3)      What is the justification for using/including male mice only?

**Response:** The majority of previous studies have focused on the sleep of male mice, with the rationale that changes in the oestrus cycle have a direct effect on sleep architecture^2^. Our original intention was to be conservative and able to compare our current work with our previously published work. However, several recent studies have highlighted that narcoleptic female mice have more cataplexy, with earlier onset of cataplexy in some models. We have now discussed this limitation in our manuscript.

4)      "AAV vector (200 nl) was loaded into a 50 μL Hamilton syringe…" Was 200 nL the volume injected into the SLD?

**Response:** Yes, 200 nL was the volume injected. We have revised the methods section to clarify this point.

5)      Avertin is the trade name. Please provide pharmacological one and source.

**Response**: We have revised the manuscript to state tribromoethanol instead of avertin.

6)      Figure 2, panel F: Title says open-loop and description in legend reads closed-loop. Please fix.

**Response:** For better clarity, we revised the manuscript to specifically indicate inhibition or activation for each experiment. In addition, instead of using the term “closed-loop” for all figures, we used the term: Wake-specific, NREM-specific or REM-specific activation or inhibition. We have clarified these terms to avoid any confusion.

7)      Figure 3, schematic C: Green shaded area seems over the LPO, not the MPA.

**Response:** Yes, the MPA is labelling the empty space in the image. The expression itself is on the lateral side compartment of the Basal forebrain. Thank you for your comments. We have revised the label of the figure to make this clearer.

Reviewer #3

This study employs optogenetic techniques to demonstrate that SLD GABAergic neurons play a role in suppressing wakefulness in both wild-type and narcoleptic mice. The findings, including rapid wakefulness induction upon neuronal silencing and the promotion of NREM sleep upon activation, are both intriguing and potentially significant. The manuscript is well-written, with clear and logically organized figures. However, the study remains predominantly descriptive, and several critical issues need to be addressed. These include the need for more rigorous anatomical validation, greater specificity in projection analysis, a deeper interpretation within the context of existing literature, and the incorporation of mechanistic insights to enhance the study's overall impact.

We thank the reviewer for their thoughtful description of our findings, and we have aimed to address the issues raised.

Major Comments

1. Uncertainty in targeting accuracy. The authors report using stereotactic coordinates (AP -5.0, ML ±0.9, DV -4.25 mm from bregma), which, according to standard mouse brain atlases such as Paxinos & Franklin, correspond more closely to the dorsomedial tegmental nucleus (DMTg) rather than the SLD. This raises a serious concern about potential mistargeting of viral injections and optic fiber placements. As a result, the observed effects on sleep-wake regulation and muscle tone might actually reflect DMTg activation or suppression, rather than specific effects mediated by SLD GABAergic neurons. More precise anatomical validation (e.g., low-magnification overviews, SLD-specific markers) is essential to interpret the findings reliably.

**Response:** We appreciate the reviewer’s concern regarding targeting accuracy. We have added more descriptions of our targeting strategies and figures confirming the localization of the viral expression and optic fiber placement. We have not based our targeting strategy solely on standard Atlas, but on previously published literature by us and others. Pioneer studies have found that lesioning a small brain structure in the dorsal pons disrupts REM sleep. This particular brain region is located under the laterodorsal tegmental nucleus (LDT) in rodents, and thus got the name sublaterodorsal tegmental nucleus (SLD) ^3–5^. The distinct anatomical boundaries or specific molecular markers that define the SLD are still under investigation. However, we focused our targeting based on where REM-active cells (e.g., expressing c-fos during REM sleep rebound) have been reported. It is generally characterized as a dorsal pontine structure situated between the laterodorsal tegmental nucleus and the trigeminal nucleus^4,6–8^. Our coordinate targeted mostly the caudal section of the SLD, consistent with prior studies of SLD^GABA^ neurons ^7,8^. These coordinates correspond to ML:± 0.9 mm DV: 4.25 mm RC: -5.34 mm based on the Allen brain atlas 3^rd^ edition. However, to account for different variables that may exist between different stereotaxic apparatus and setups, the coordinates were empirically refined through pilot adjustments. After each experiment, we verified targeting accuracy through histological confirmation. We have clarified these points in the method section of the manuscript, and we have added clear low-magnification example panels to Supplementary Figure 1 to depict the spread of the viral vector across the rostro-caudal, medio-lateral and dorso-ventral axes of the SLD and how the optic fibers were placed above the SLD. This will allow the reader to appreciate our targeting and compare the data to what has been previously published by us and others^9,10^.

2. Anatomical validation of the viral targeting and fiber placement is insufficient. The manuscript provides only a high-magnification image of viral expression in Figure 1, with no overview of the full injection spread or fiber tract location. This makes it difficult to confirm that manipulations were restricted to the SLD. Comprehensive validation, including representative low-power images across experimental animals, is needed to demonstrate accurate and consistent targeting.

**Response:** As described in our response above, we have included representative low-magnification images illustrating the extent of viral expression and the placement of optic fibers within the SLD. These images will accompany the high-magnification figure currently presented to provide a comprehensive validation of anatomical targeting. This figure can be found in Supplementary Figure 1 A.

3. Similar issues arise in the tracing experiment: the authors should also show viral expression within the SLD to confirm correct injection sites. Moreover, projection images are shown only at low magnification, making it unclear whether observed fibers represent synaptic terminals or passing axons. High-magnification images showing terminal boutons in downstream targets are needed. In addition, the authors focus mainly on wake-related projections, but other well-established SLD targets—such as the ventromedial medulla (VMM)—which plays a critical role in REM atonia, should be examined. A more comprehensive whole-brain projection map would allow readers to better evaluate the output profile of SLD GABAergic neurons.

**Response:** We thank the reviewer for their comment. We added a figure showing the injection site for the tracing data in Figure 3I. We also added high-magnification images showing the neural projection and potential boutons in the downstream target regions (Figure 3). We agree with the reviewer’s comments regarding the limitations of our tracing data. The current tracing experiment provides only preliminary information about the connection network of SLD^GABA^ neurons. Specifically, it is constrained by (1) uncertainty as to whether observed branches in distal regions represent passing fibers or true synaptic connections and (2) lack of confirmation that inputs from SLD^GABA^ neurons modulate activity of downstream targets.

The inclusion of the tracing dataset was intended as an exploratory analysis to illustrate the anatomical diversity of SLD^GABA^ axonal projections and to introduce future research directions. Our primary objective in this study was to characterize the functional role of SLD^GABA^ neurons in sleep–wake regulation in both healthy and narcoleptic mice. We would like to highlight that identifying the functional connectivity of all candidate brain regions is beyond the scope of our current study, nor is it feasible within the time provided for revision. We explicitly acknowledge these limitations and considerations in our discussion of future studies.

Importantly, our current tracing experiment did not reveal evidence of SLD^GABA^ projections to the ventromedial medulla (VMM). Previous studies indicate that SLD projections to the VMM are primarily mediated by glutamate neurons, while SLD^GABA^ neurons instead project to the spinal cord^7,11,12^.

4. Interpretation of Projections Lacks Functional Specificity. The authors report that SLD GABAergic neurons project to numerous wake- and motor-related regions—including the medial septum, basal forebrain, lateral hypothalamus (LH), tuberomammillary nucleus (TMN), supramammillary nucleus, interpeduncular nucleus, ventrolateral periaqueductal gray, vestibular nuclei, and even the spinal cord ventral horn—Based on this, they position SLD GABAergic neurons as a key nucleus in the neural circuits controlling arousal state transitions (Page36, Line37).
However, this interpretation is problematic. Many of these downstream targets are functionally heterogeneous. For example, the LH includes both wake-promoting orexin neurons and sleep-promoting MCH and GABAergic neurons, which are spatially intermingled. Without identifying the specific cell types targeted by SLD GABA inputs, it is unclear whether these projections suppress arousal, promote sleep, or exert a more complex effect.

Similar concerns apply to other regions such as the basal forebrain and TMN, which contain cholinergic, GABAergic, and glutamatergic neurons with distinct and sometimes opposing roles. Thus, projection anatomy alone is insufficient to support functional conclusions.
Moreover, the vlPAG is canonically regarded as a REM-off area that inhibits the SLD. The finding that activation of SLD GABAergic neurons has no detectable effect on REM sleep, despite projections to the vlPAG, is unexpected and warrants further explanation.

To strengthen the interpretation of whether these projections are functionally relevant, it would be helpful if the authors considered projection-specific manipulations. For example, selectively activating or inhibiting SLD GABAergic terminals in specific downstream targets (such as TMN, LH, or MS) and measuring behavioral or physiological outcomes could clarify whether suppression of wakefulness is mediated by a dominant downstream site or distributed effects.

**Response:** We appreciate the reviewer’s thoughtful comments. We revised the manuscript to further elaborate on the diverse and complex roles of SLD GABAergic projections.

We agree with the reviewers that the SLD^GABA^ neurons are heterogeneous in nature. Many of which have functions beyond sleep-wake regulation. We will highlight this in the discussion to be transparent with the readers about the complexity of SLD^GABA^ network and the need for further validation of the complete neural network of SLD^GABA^ neurons in wake-suppression.

Our tracing data provide only a general overview of projection patterns. It was not our intention to identify specific synaptic contacts or the functional significance of individual pathways. Future studies will employ projection-specific manipulations to examine how SLD GABAergic neurons interact with distinct downstream targets—such as the TMN, LH, and MS—to regulate wakefulness and sleep. Given the extensive and distributed nature of these projections, a comprehensive functional dissection of all pathways is beyond the scope of our current work. Our manuscript already contains 6 main figures and 6 supplementary figures with an extensive number of panels. We believe that additional projection-specific experiments would dilute the main message on the functional role of SLD^GABA^ neurons in both healthy and narcoleptic mice.

Finally, the vlPAG^GABA^ neurons are the main REM-off neurons in the brainstem, while the SLD^GABA^ neurons were thought to induce REM sleep by silencing vlPAG^GABA^ neurons (Lu et al. 2006)^7^. In this pioneer work, they reported that lesioning the “GABAergic field” within the SLD suppressed REM sleep; however, this lesion was not cell type-specific. Then, in a follow-up study, the same Lab (Krenzer et. al., 2011) showed that genetic deletion of GABA neurotransmission (VGAT) from the SLD did not produce any effect on REM sleep^11^. While informative, this early work had limitations for assessing sleep-wake function that we discuss in our manuscript. Although the “flip-flop” switch model has been widely influential, the specific functional contribution of SLD^GABA^ neurons to REM sleep control has remained unresolved. We address this issue in the Discussion of the revised manuscript

5. Previous studies have extensively investigated the neural circuits through which orexin system dysfunction leads to narcolepsy, including pathways involving the SLD. Beyond the well-characterized orexin-vlPAG/LPT-SLD circuit, anatomical evidence shows that orexinergic neurons also project directly to the SLD, suggesting both direct and indirect pathways through which orexin may modulate SLD activity related to REM sleep and REM sleep atonia.
Although the authors briefly acknowledge the limitation that "the precise mechanisms by which orexin loss potentiates SLDGABA function require further investigation," it is strongly recommended that they incorporate a more detailed discussion of how the loss of orexin signaling—particularly the elimination of direct inputs to SLD neurons—may alter local GABAergic and glutamatergic dynamics and contribute to the observed sleep attacks and cataplexy.

**Response:** We agree with the reviewer’s recommendation to elaborate on how orexin loss may potentiate GABA SLD neuron function. Previous studies have demonstrated that orexin neurons send direct projections to the SLD, and immunostaining confirms that these projections contain orexin peptides^13,14^. Furthermore, Watson et al. (2008) reported that orexin infusion into the SLD enhances local GABAergic neurotransmission and promotes wakefulness, indicating a direct regulatory pathway^15^.

Indirect interactions may also occur through the ventrolateral periaqueductal gray (vlPAG), which receives dense orexinergic input and, in turn, sends inhibitory GABAergic projections to the SLD ^7,16,17^. Thus, the loss of orexin signalling could disrupt this modulatory relay, leading to disinhibited SLD activity. Finally, orexin has been shown to attenuate GABAergic inhibitory postsynaptic currents^18^ and to reduce GABA_A_ receptor–mediated hyperpolarization via orexin-1 receptor activation^19^. These mechanisms suggest that orexin normally counteracts GABA SLD–mediated inhibition at the postsynaptic level. Taken together, we propose that one or a combination of these mechanisms—direct, indirect, or postsynaptic—may explain how orexin loss enhances GABA SLD neuron–mediated suppression of wakefulness, and we fully discuss these points in our discussion.

6. In this study, the roles and mechanisms of SLD GABAergic neurons are based on artificial manipulations of cell activity. Investigating when SLD GABAergic neurons are naturally activated or inhibited under physiological or pathological conditions holds greater significance and is worthy of further exploration.

**Response:** We acknowledge the significance of characterizing the natural activity of SLD^GABA^ neurons. Prior research has already demonstrated that these neurons display state-dependent firing patterns, with distinct subpopulations exhibiting activity profiles of either REM > NREM > Wake or Wake > REM > NREM ^20,21^.

Our optogenetic results complement this evidence by showing that activation of SLD^GABA^ neurons suppresses wakefulness and muscle tone, while their inhibition promotes arousal. Our findings suggest that SLD^GABA^ neurons function to suppress wake-related features during both sleep and wakefulness, by fine-tuning motor output and brain activity across the states. However, whether these functions arise from distinct subtypes of SLD^GABA^ neurons remains to be determined and is beyond the scope of our study.

While prior studies have characterized their activity under normal sleep–wake conditions, the dynamics of these neurons in pathological states, such as narcolepsy, remain largely unexplored. We plan to address this gap in future work by employing calcium imaging with microendoscope^21^ to get a more precise activity pattern of SLD^GABA^ neurons, as general fiber photometry recordings would not distinguish between several subpopulations. This will help elucidate how the loss of orexin signalling alters the natural activation patterns of these neurons. However, this is a challenging experiment as the SLD is deep in the brainstem and requires the objective to go through a big portion of the cerebellum, and it is not realistic to complete and include this data set in time for the revision. However, we discuss this future research direction in the last paragraph of this manuscript.

**References**

1.	Mochizuki, T. *et al.* Behavioral state instability in orexin knock-out mice. *J Neurosci* **24**, 6291–6300 (2004).

2.	Schmidt, M. H. & Bassetti, C. L. A. Gender differences in narcolepsy: What are recent findings telling us? *Sleep* **45**, zsac126 (2022).

3.	Boissard, R., Fort, P., Gervasoni, D., Barbagli, B. & Luppi, P.-H. Localization of the GABAergic and non-GABAergic neurons projecting to the sublaterodorsal nucleus and potentially gating paradoxical sleep onset. *Eur J Neurosci* **18**, 1627–1639 (2003).

4.	Boissard, R. *et al.* The rat ponto-medullary network responsible for paradoxical sleep onset and maintenance: a combined microinjection and functional neuroanatomical study: REM sleep brainstem structures in rats. *European Journal of Neuroscience* **16**, 1959–1973 (2002).

5.	Luppi, P.-H. *et al.* Paradoxical (REM) sleep genesis: The switch from an aminergic–cholinergic to a GABAergic–glutamatergic hypothesis. *J Physiol Paris* **100**, 271–283 (2007).

6.	Torontali, Z. A., Fraigne, J. J., Sanghera, P., Horner, R. & Peever, J. The Sublaterodorsal Tegmental Nucleus Functions to Couple Brain State and Motor Activity during REM Sleep and Wakefulness. *Curr Biol* **29**, 3803-3813.e5 (2019).

7.	Lu, J., Sherman, D., Devor, M. & Saper, C. B. A putative flip-flop switch for control of REM sleep. *Nature* **441**, 589–594 (2006).

8.	Maloney, K. J., Mainville, L. & Jones, B. E. Differential c-Fos Expression in Cholinergic, Monoaminergic, and GABAergic Cell Groups of the Pontomesencephalic Tegmentum after Paradoxical Sleep Deprivation and Recovery. *J Neurosci* **19**, 3057–3072 (1999).

9.	Torontali ZA, Fraigne JJ, Sanghera P, Horner R and Peever J. The Sublaterodorsal Tegmental Nucleus Functions to Couple Brain State and Motor Activity during REM Sleep and Wakefulness. Curr Bio. (2020) 29:3803-3813.

10.	Ngo, S. *et al.* Identification of glutamatergic inputs to the sublaterodorsal tegmental nucleus and their role in REM sleep control. *Sleep* zsag014 (2026) doi:10.1093/sleep/zsag014.

11.	Krenzer, M. *et al.* Brainstem and Spinal Cord Circuitry Regulating REM Sleep and Muscle Atonia. *PLoS ONE* **6**, e24998 (2011).

12.	Valencia Garcia, S. *et al.* Genetic inactivation of glutamate neurons in the rat sublaterodorsal tegmental nucleus recapitulates REM sleep behaviour disorder. *Brain* **140**, 414–428 (2017).

13.	Peyron, C. *et al.* Neurons containing hypocretin (orexin) project to multiple neuronal systems. *J Neurosci* **18**, 9996–10015 (1998).

14.	Feng, H. *et al.* Orexin signaling modulates synchronized excitation in the sublaterodorsal tegmental nucleus to stabilize REM sleep. *Nat Commun* **11**, 3661 (2020).

15.	Watson, C. J., Soto-Calderon, H., Lydic, R. & Baghdoyan, H. A. Pontine reticular formation (PnO) administration of hypocretin-1 increases PnO GABA levels and wakefulness. *Sleep* **31**, 453–464 (2008).

16.	Date, Y. *et al.* Orexins, orexigenic hypothalamic peptides, interact with autonomic, neuroendocrine and neuroregulatory systems. *Proc Natl Acad Sci U S A* **96**, 748–753 (1999).

17.	Weber, F. *et al.* Regulation of REM and Non-REM Sleep by Periaqueductal GABAergic Neurons. *Nat Commun* **9**, 354 (2018).

18.	Xi, M., Fung, S. J., Yamuy, J. & Chase, M. H. Interactions between hypocretinergic and GABAergic systems in the control of activity of neurons in the cat pontine reticular formation. *Neuroscience* **298**, 190–199 (2015).

19.	Sachidanandan, D., Reddy, H. P., Mani, A., Hyde, G. J. & Bera, A. K. The Neuropeptide Orexin-A Inhibits the GABAA Receptor by PKC and Ca2+/CaMKII-Dependent Phosphorylation of Its β1 Subunit. *J Mol Neurosci* **61**, 459–467 (2017).

20.	Boucetta, S., Cissé, Y., Mainville, L., Morales, M. & Jones, B. E. Discharge profiles across the sleep-waking cycle of identified cholinergic, GABAergic, and glutamatergic neurons in the pontomesencephalic tegmentum of the rat. *J Neurosci* **34**, 4708–4727 (2014).

21.	Cox, J., Pinto, L. & Dan, Y. Calcium imaging of sleep–wake related neuronal activity in the dorsal pons. *Nat Commun* **7**, 10763 (2016).
