# Supplementary material for: GABA neurons in the sublaterodorsal tegmental nucleus suppress wakefulness in healthy and narcoleptic mice
Source: PLoS Biol. 2026 Jul 8;24(7):e3003303. doi: 10.1371/journal.pbio.3003303 (PMC13345269; doi:10.1371/journal.pbio.3003303)
Supplement: Attachment — Submitted filename: PLOSbio response to reviewer-revision2-FINAL.docx [file pbio.3003303.s021.docx]

Reviewer #1 [Akihiro Yamanaka]: This revised manuscript is improved in several respects. The anatomical clarification, revised terminology, and summary schematic make the study easier to follow. The finding that SLD GABAergic neurons suppress wakefulness and can precipitate sleep attacks in orexin-deficient mice is interesting and potentially important.

We thank Dr. Yamanaka for his review and useful comments. We have followed the recommendations and addressed each point below.

1. Closed-loop performance still requires more transparent reporting.
The manuscript states that the success rate of closed-loop illumination was 99.16%. It is unclear whether this value represents the overall accuracy across all state-dependent closed-loop conditions or only the best-performing condition. Please report the accuracy separately for each vigilance state. In addition, please provide the latency from state detection to light delivery for each condition.

**Response:** Thank you for your comment. As requested by the reviewer, we added the following descriptions in the method section: 1) The success rate was calculated from the pool of all optogenetic stimulation trials in this study, 2) we now provide the success rate of closed-loop illumination for each arousal state and 3) we have clarified how a stable transition into each arousal state is identified, and provided the latency to light delivery.

2. The mechanistic discussion of SLD GABA neurons remains too definitive.
The prior literature does not yet establish a single physiological role for SLD GABA neurons. Rather, existing works suggest functional heterogeneity, with possible contributions to REM-state gating, wakefulness, and other aspects of pontine network activity. The current findings are interesting, but the Discussion should better acknowledge this complexity and avoid overly definitive statements.

**Response:** We appreciate this concern and agree that the prior literature does not support a single physiological role for all SLD^GABA^ neurons. In response, we revised the Discussion to temper our interpretation and to explicitly acknowledge the likely functional heterogeneity of the SLD^GABA^ population. We removed overly definitive language suggesting that the entire SLD^GABA^ population serves a single primary function and replaced it with more cautious wording. We also added a discussion point that our approach might have masked functional diversity and that subpopulations of GABA neurons might contribute to REM-state gating or broader pontine network activity.

3. The discussion of orexin signaling should be corrected and clarified.
The manuscript states that local orexin infusion into the SLD enhances local GABAergic signaling and promotes wakefulness, but the cited study appears to have examined infusion into the pontine reticular formation rather than the SLD itself. This should be corrected. More broadly, the Discussion should more clearly distinguish among direct effects of orexin within the SLD, indirect circuit effects, and possible downstream postsynaptic interactions.

**Response:** We thank the Reviewer for identifying this issue. We revised the Discussion section to correct our prior wording regarding the *Watson et al.* study. We now clarify that the study examined orexin infusion into the pontine reticular formation (PnO) rather than the SLD itself. We further revised this section to clarify two potential indirect pathways through which lateral hypothalamic (LH) orexin neurons may influence wake-suppressing SLD^GABA^ neurons. First, based on the findings from *Watson et al.* study, orexin may influence wake-suppressing SLD^GABA^ neurons indirectly through local SLD GABAergic interneurons, rather than through a direct action onto the wake-suppressing SLD^GABA^ neurons themselves. Second, we clarified that orexin neurons may also influence SLDG^ABA^ neurons indirectly though the activation of ventrolateral periaqueductal grey (vlPAG), which send inhibitory input to the SLD. We also have clarified how orexin may also modify SLD^GABA^-associated signaling at a postsynaptic level. Finally, we tempered the language throughout this section to make clear that these mechanisms remain speculative, and that the relevant synaptic pathways and cell-type-specific effects have not yet been directly established.

4. The discrepancy between the orexin literature and the present optogenetic results should be addressed more directly. In the current model, orexin is thought to promote wakefulness, whereas optogenetic activation of SLD GABA neurons suppresses wakefulness and triggers sleep attacks in orexin-deficient mice. This suggests that the circuitry is more complex than the current Discussion implies, possibly because of regional differences, cell-type heterogeneity, or differences between local neuromodulation and synchronous optogenetic activation. This point should be discussed explicitly.

**Response:** We agree with the reviewer and have revised the Discussion section to address these points. We now explicitly discuss that the relationship between wake-promoting orexin neurons and wake-suppressing SLD^GABA^ neurons is complex. As mentioned in the question above, we further discussed that orexin may regulate SLD^GABA^ neurons through several indirect circuit mechanisms, rather than through a direct mechanism. We also expand on the possible functional heterogeneity of the SLD^GABA^ neurons, and on the possible downstream postsynaptic modulation of SLD^GABA^ neurons by orexin-mediated mechanism.

5. The "final common pathway" interpretation remains overstated. Although the silencing experiments are provocative, the current data do not yet establish that SLD GABA neurons constitute a final common pathway for both sleep attacks and cataplexy. The relevant downstream targets and neuronal subtypes remain unresolved, and the conclusions should therefore be tempered.

**Response:** We thank the reviewer for this comment and have revised the relevant paragraph to substantially soften this interpretation. We now state that the ability of SLD^GABA^ silencing to terminate both symptoms raises the possibility that these neurons contribute to overlapping circuit mechanisms, while acknowledging that the current data do not establish such a pathway definitively. We propose that additional studies will be required to define the precise circuit mechanisms linking SLD^GABA^ neurons to both sleep attacks and cataplexy.
