# Supplementary material for: GABA neurons in the sublaterodorsal tegmental nucleus suppress wakefulness in healthy and narcoleptic mice
Source: PLoS Biol. 2026 Jul 8;24(7):e3003303. doi: 10.1371/journal.pbio.3003303 (PMC13345269; doi:10.1371/journal.pbio.3003303)
Supplement: Attachment — Submitted filename: PLOSbio response to reviewer-revision3-FINAL.docx [file pbio.3003303.s022.docx]

Title: We would like to remove the '(SLD)' abbreviation from the title, in accordance with our stylistic preferences. Is this acceptable to you?

Response: We agree with the change, and we have removed the abbreviation from the title.

Financial disclosure statement: Please confirm that all relevant grant numbers have been included in the Financial disclosure statement in the manuscript details. Please also add links to the funding agencies to the statement.

Response: We confirm that all relevant grant numbers have been included in our statement.

Ethics: Please create a new, first sub-heading of the Materials and Methods section titled "Ethics statement". Please move all relevant ethical approval information under this sub-heading, including the protocol/permit/project license.

Response: We have created a sub-heading section and moved the relevant information under it.

Data: Please supply the numerical values either in a supplementary excel file or as a permanent DOI’d deposition for the following figures panels, and ensure that these are accessible now:

1EIKNOPQ
2CDEIJKL (right subpanels)
4EFGJKLMNO (right subpanels)
5BCFK
S1GHIJKL (right subpanels)
S2ADGJMNOPQRSTUVWXY (right subpanels)
S3FGHIJK (right subpanels)
S4ABCDEFOPQRSTU (right subpanels)
S6IKLMNOP (right subpanels)

Please cite the location of the data clearly in all relevant main and supplementary Figure legends

Response: We have added the requested data to individual Excel files for each listed figure, and each panel is on a separate tab for the corresponding figure. We have stated for each figure legend the corresponding supplementary data (i.e., S_Data_Fig). 

Supplement format: Please move the Materials and Methods into the main manuscript document. Please also move the supplementary figure legends into the main text, and upload each supplementary figure file individually, as "Supporting Information". Please also integrate the supplementary references into the main reference list, and remove any redundancies.

Response: We have moved the information to the main manuscript as requested

 Code availability: Per journal policy, if you have generated any custom code or scripts during the course of this investigation, we require that you make it available without restrictions. Please ensure that the code is sufficiently well documented and reusable, and that your Data Statement in the Editorial Manager submission system accurately describes where your code can be found. Please also ensure that you choose a license for your code and include a Readme file.

Response: We did not generate any custom code for this manuscript.
