# Supplementary material for: GABA neurons in the sublaterodorsal tegmental nucleus suppress wakefulness in healthy and narcoleptic mice
Source: PLoS Biol. 2026 Jul 8;24(7):e3003303. doi: 10.1371/journal.pbio.3003303 (PMC13345269; doi:10.1371/journal.pbio.3003303)
Supplement: Peer review history

# Editor report: Decision Letter 0

**DOI:** 10.1371/journal.pbio.3003303.r001

**Authors:** Taylor Hart, PhD

7 Jul 2025

Dear Dr Fraigne,

Thank you for submitting your manuscript entitled "GABA neurons in the sublaterodorsal tegmental (SLD) nucleus suppress wakefulness in wild-type and narcoleptic mice" for consideration as a Research Article by PLOS Biology.

Thank you also for your patience while we discussed your submission. Your manuscript has now been evaluated by the PLOS Biology editorial staff, as well as by an academic editor with relevant expertise, and I am writing to let you know that we would like to send your submission out for external peer review.

However, before we can send your manuscript to reviewers, we need you to complete your submission by providing the metadata that is required for full assessment. To this end, please login to Editorial Manager where you will find the paper in the 'Submissions Needing Revisions' folder on your homepage. Please click 'Revise Submission' from the Action Links and complete all additional questions in the submission questionnaire.

Once your full submission is complete, your paper will undergo a series of checks in preparation for peer review. After your manuscript has passed the checks it will be sent out for review. To provide the metadata for your submission, please Login to Editorial Manager (https://www.editorialmanager.com/pbiology) within two working days, i.e. by Jul 09 2025 11:59PM.

If your manuscript has been previously peer-reviewed at another journal, PLOS Biology is willing to work with those reviews in order to avoid re-starting the process. Submission of the previous reviews is entirely optional and our ability to use them effectively will depend on the willingness of the previous journal to confirm the content of the reports and share the reviewer identities. Please note that we reserve the right to invite additional reviewers if we consider that additional/independent reviewers are needed, although we aim to avoid this as far as possible. In our experience, working with previous reviews does save time.

If you would like us to consider previous reviewer reports, please edit your cover letter to let us know and include the name of the journal where the work was previously considered and the manuscript ID it was given. In addition, please upload a response to the reviews as a 'Prior Peer Review' file type, which should include the reports in full and a point-by-point reply detailing how you have or plan to address the reviewers' concerns.

During the process of completing your manuscript submission, you will be invited to opt-in to posting your pre-review manuscript as a bioRxiv preprint. Visit http://journals.plos.org/plosbiology/s/preprints for full details. If you consent to posting your current manuscript as a preprint, please upload a single Preprint PDF.

Feel free to email us at plosbiology@plos.org if you have any queries relating to your submission.

Kind regards,

Taylor

Taylor Hart, PhD,

Associate Editor

PLOS Biology

thart@plos.org

# Peer review: Decision Letter 1

**DOI:** 10.1371/journal.pbio.3003303.r002

**Authors:** Taylor Hart, PhD

19 Aug 2025

Dear Dr Fraigne,

My name is Luke Smith - I am writing on behalf of my colleague, Dr. Taylor Hart while she is away on vacation, to send you the reviewer feedback for your PLOS Biology submission, "GABA neurons in the sublaterodorsal tegmental (SLD) nucleus suppress wakefulness in wild-type and narcoleptic mice". Your manuscript has been evaluated by the PLOS Biology editors, an Academic Editor with relevant expertise, and by several independent reviewers.

As you will see in the reviewer reports, which can be found at the end of this email, although the reviewers find the work potentially interesting, they have also raised a substantial number of important concerns. Based on their specific comments and following discussion with the Academic Editor, it is clear that a substantial amount of work would be required to meet the criteria for publication in PLOS Biology. However, given our and the reviewer interest in your study, we would be open to inviting a comprehensive revision of the study that thoroughly addresses all the reviewers' comments. Given the extent of revision that would be needed, we cannot make a decision about publication until we have seen the revised manuscript and your response to the reviewers' comments. Your revised manuscript would need to be seen by the reviewers again, but please note that we would not engage them unless their main concerns have been addressed.

Having discussed the reviews with the Academic Editor, we think that a revised manuscript will need to thoroughly strengthen the main findings reported here, including by providing additional controls, validation studies, analyses, and clarifications as detailed by the reviewers. We also encourage you to experimentally address reviewer 3's point 6, and provide further mechanistic insights in physiologically relevant conditions, if possible.

In addition to these revisions, you will need to complete some formatting changes, which you will receive in a follow up email. A member of our team will be in touch with a set of requests shortly.

We appreciate that these requests represent a great deal of extra work, and we are willing to relax our standard revision time to allow you 6 months to revise your study. Please email us (plosbiology@plos.org) if you have any questions or concerns, or envision needing a (short) extension.

At this stage, your manuscript remains formally under active consideration at our journal; please notify us by email if you do not intend to submit a revision so that we may withdraw it.

**IMPORTANT - SUBMITTING YOUR REVISION**

Your revisions should address the specific points made by each reviewer. Please submit the following files along with your revised manuscript:

1. A 'Response to Reviewers' file - this should detail your responses to the editorial requests, present a point-by-point response to all of the reviewers' comments, and indicate the changes made to the manuscript.

*NOTE: In your point by point response to the reviewers, please provide the full context of each review. Do not selectively quote paragraphs or sentences to reply to. The entire set of reviewer comments should be present in full and each specific point should be responded to individually, point by point.

You should also cite any additional relevant literature that has been published since the original submission and mention any additional citations in your response.

2. In addition to a clean copy of the manuscript, please also upload a 'track-changes' version of your manuscript that specifies the edits made. This should be uploaded as a "Revised Article with Changes Highlighted " file type.

*Resubmission Checklist*

When you are ready to resubmit your revised manuscript, please refer to this resubmission checklist: https://plos.io/Biology_Checklist

To submit a revised version of your manuscript, please go to https://www.editorialmanager.com/pbiology/ and log in as an Author. Click the link labelled 'Submissions Needing Revision' where you will find your submission record.

Please make sure to read the following important policies and guidelines while preparing your revision:

*Published Peer Review*

Please note while forming your response, if your article is accepted, you may have the opportunity to make the peer review history publicly available. The record will include editor decision letters (with reviews) and your responses to reviewer comments. If eligible, we will contact you to opt in or out. Please see here for more details:

https://blogs.plos.org/plos/2019/05/plos-journals-now-open-for-published-peer-review/

*PLOS Data Policy*

Please note that as a condition of publication PLOS' data policy (http://journals.plos.org/plosbiology/s/data-availability) requires that you make available all data used to draw the conclusions arrived at in your manuscript. If you have not already done so, you must include any data used in your manuscript either in appropriate repositories, within the body of the manuscript, or as supporting information (N.B. this includes any numerical values that were used to generate graphs, histograms etc.). For an example see here: http://www.plosbiology.org/article/info%3Adoi%2F10.1371%2Fjournal.pbio.1001908#s5

*Blot and Gel Data Policy*

We require the original, uncropped and minimally adjusted images supporting all blot and gel results reported in an article's figures or Supporting Information files. We will require these files before a manuscript can be accepted so please prepare them now, if you have not already uploaded them. Please carefully read our guidelines for how to prepare and upload this data: https://journals.plos.org/plosbiology/s/figures#loc-blot-and-gel-reporting-requirements

*Protocols deposition*

To enhance the reproducibility of your results, we recommend that if applicable you deposit your laboratory protocols in protocols.io, where a protocol can be assigned its own identifier (DOI) such that it can be cited independently in the future. Additionally, PLOS ONE offers an option for publishing peer-reviewed Lab Protocol articles, which describe protocols hosted on protocols.io. Read more information on sharing protocols at https://plos.org/protocols?utm_medium=editorial-email&utm_source=authorletters&utm_campaign=protocols

Thank you again for your submission to our journal. We hope that our editorial process has been constructive thus far, and we welcome your feedback at any time. Please don't hesitate to contact us if you have any questions or comments.

Sincerely,

Luke

Lucas Smith, PhD

Senior Editor

PLOS Biology

lsmith@plos.org

on behalf of

Taylor Hart, PhD,

Associate Editor

PLOS Biology

thart@plos.org

-----------------------------------------

REVIEWS:

Reviewer #1, Akihiro Yamanaka (note, reviewer 1 has signed this review): This study focuses on the SLDGABA neurons in the regulation of sleep/wakefulness. Through axonal tracing, the authors demonstrated that SLDGABA projects to multiple wake-promoting brain regions, providing an anatomical basis for its function. The most novel aspect of this work is the investigation of the role of SLDGABA neurons in regulating sleep attacks in narcolepsy. In ORX-/- mice, activation of SLDGABA induces sleep attacks, whereas inhibition of SLDGABA causes immediate arousal in mice undergoing a sleep attack. Moreover, silencing of SLDGABA also terminates cataplexy, restoring motor activity. The finding that SLDGABA is both sufficient and necessary for the regulation of sleep attacks highlights its potential as a promising therapeutic target for narcolepsy. This reviewer suggests the following points to improve the manuscripts.

Major comments

1)Activation of SLDGABA immediately induced NREM sleep in orexin-/- mice is interesting. However, it is not clear whether this is because sleep pressure is higher in these mice or the orexin signal affects the base activity of these neurons. To make it clear, please test whether the activation of SLDGABA in the mild sleep deprivation condition in orexin+/+ mice shortens the latency to NREM sleep or not.

2)Figure 1, Open-loop inhibition, it is clear that light off increased the EMG signal as well. Is this because rebound firing induces wakefulness?

3)Figure 1, open-loop stimulation and closed-loop stimulation in the Figure is confusing since it is an inhibition, and Figure 1H, 1J and 1L indicate inhibition.

4)Figure 1L, inhibition during wake induced theta activity. Is this wakefulness comparable to the normal wakefulness?

5)Please add the success rate of closed-loop illumination in each state. This means how accurately the algorithm predicts the state of the animal.

6)Zoltan A. Torontali, John Peever et al., Curr Biol 2019 showed that inhibition of SLD neurons inhibited cataplexy in orexin-/- mice. Similarly, does inhibition of SLD GABA inhibit the number of cataplexy episodes?

7)Figure 3 showed that SLD GABA neurons densely innervated many brain areas. However, the authors are just focusing on orexin neurons in the LH, which is not the densest innervation. It's better to add some descriptions of the reason to focus on this pathway.

8)Kashiwagi et al., Cell 2024, Figure 4 have already indicated that activation of GABAergic projection from SLD to BF induced NREM sleep. This study confirms this. The author should mention this.

9)This reviewer suggests that the author include a schematic drawing of the summary.

Minor comments:

1)All Figures and text, add a space between the number and the unit.

2)Figure 1O, 1P and 1Q. Please standardize the way of writing, Arch, and mCherry.

3)Figure 4C, This is closed-loop stimulation, probably not the inhibition.

4)Figure 1G should indicate which color indicates which state.

Reviewer #2: In this study, Lee and colleagues explored the role of GABAergic neurons within the sublaterodorsal tegmental nucleus (SLD) in sleep-wake regulation, in wild-type and narcoleptic mice. This is novel and timely. SLD glutamatergic neurons play a key role in arousal state control, especially REM sleep. By contrast, whether SLD GABA neurons modulate sleep-wake states remains unknown. The experiments, including optogenetic manipulation and anterograde viral tracing mapping of efferent projections, were rigorously conducted and well controlled. The data are compelling and clearly presented. The main findings are that SLD GABAergic neurons suppress wakefulness in wild type mice. In the narcolepsy mouse model, their activation during wakefulness produced rapid NREM onset ("sleep attacks" characteristic of narcolepsy), but it did not trigger or worsen cataplexy. Optical silencing of these neurons reduced the sleep attacks and cataplexy. Thus, these results suggest a role for SLD GABAergic neurons in the regulation of sleep attacks or excessive sleepiness in narcolepsy.

Please find a few minor comments below:

1)Typo in Intro, line 20: "systems"

2)For more clarity, I suggest that "wild-type" mice is defined the first time it is used. I was initially confused by reading wild-type when referring to non-orexin deficient, transgenic Vgat-Cre mice.

3)What is the justification for using/including male mice only?

4)"AAV vector (200 nl) was loaded into a 50 μL Hamilton syringe…" Was 200 nL the volume injected into the SLD?

5)Avertin is the trade name. Please provide pharmacological one and source.

6)Figure 2, panel F: Title says open-loop and description in legend reads closed-loop. Please fix.

7)Figure 3, schematic C: Green shaded area seems over the LPO, not the MPA.

Reviewer #3: This study employs optogenetic techniques to demonstrate that SLD GABAergic neurons play a role in suppressing wakefulness in both wild-type and narcoleptic mice. The findings, including rapid wakefulness induction upon neuronal silencing and the promotion of NREM sleep upon activation, are both intriguing and potentially significant. The manuscript is well-written, with clear and logically organized figures. However, the study remains predominantly descriptive, and several critical issues need to be addressed. These include the need for more rigorous anatomical validation, greater specificity in projection analysis, a deeper interpretation within the context of existing literature, and the incorporation of mechanistic insights to enhance the study's overall impact.

Major Comments

1. Uncertainty in targeting accuracy. The authors report using stereotactic coordinates (AP -5.0, ML ±0.9, DV -4.25 mm from bregma), which, according to standard mouse brain atlases such as Paxinos & Franklin, correspond more closely to the dorsomedial tegmental nucleus (DMTg) rather than the SLD. This raises a serious concern about potential mistargeting of viral injections and optic fiber placements. As a result, the observed effects on sleep-wake regulation and muscle tone might actually reflect DMTg activation or suppression, rather than specific effects mediated by SLD GABAergic neurons. More precise anatomical validation (e.g., low-magnification overviews, SLD-specific markers) is essential to interpret the findings reliably.

2. Anatomical validation of the viral targeting and fiber placement is insufficient. The manuscript provides only a high-magnification image of viral expression in Figure 1, with no overview of the full injection spread or fiber tract location. This makes it difficult to confirm that manipulations were restricted to the SLD. Comprehensive validation, including representative low-power images across experimental animals, is needed to demonstrate accurate and consistent targeting.

3. Similar issues arise in the tracing experiment: the authors should also show viral expression within the SLD to confirm correct injection sites. Moreover, projection images are shown only at low magnification, making it unclear whether observed fibers represent synaptic terminals or passing axons. High-magnification images showing terminal boutons in downstream targets are needed. In addition, the authors focus mainly on wake-related projections, but other well-established SLD targets—such as the ventromedial medulla (VMM)—which plays a critical role in REM atonia, should be examined. A more comprehensive whole-brain projection map would allow readers to better evaluate the output profile of SLD GABAergic neurons.

4. Interpretation of Projections Lacks Functional Specificity. The authors report that SLD GABAergic neurons project to numerous wake- and motor-related regions—including the medial septum, basal forebrain, lateral hypothalamus (LH), tuberomammillary nucleus (TMN), supramammillary nucleus, interpeduncular nucleus, ventrolateral periaqueductal gray, vestibular nuclei, and even the spinal cord ventral horn—Based on this, they position SLD GABAergic neurons as a key nucleus in the neural circuits controlling arousal state transitions (Page36, Line37).

However, this interpretation is problematic. Many of these downstream targets are functionally heterogeneous. For example, the LH includes both wake-promoting orexin neurons and sleep-promoting MCH and GABAergic neurons, which are spatially intermingled. Without identifying the specific cell types targeted by SLD GABA inputs, it is unclear whether these projections suppress arousal, promote sleep, or exert a more complex effect.

Similar concerns apply to other regions such as the basal forebrain and TMN, which contain cholinergic, GABAergic, and glutamatergic neurons with distinct and sometimes opposing roles. Thus, projection anatomy alone is insufficient to support functional conclusions.

Moreover, the vlPAG is canonically regarded as a REM-off area that inhibits the SLD. The finding that activation of SLD GABAergic neurons has no detectable effect on REM sleep, despite projections to the vlPAG, is unexpected and warrants further explanation.

To strengthen the interpretation of whether these projections are functionally relevant, it would be helpful if the authors considered projection-specific manipulations. For example, selectively activating or inhibiting SLD GABAergic terminals in specific downstream targets (such as TMN, LH, or MS) and measuring behavioral or physiological outcomes could clarify whether suppression of wakefulness is mediated by a dominant downstream site or distributed effects.

5. Previous studies have extensively investigated the neural circuits through which orexin system dysfunction leads to narcolepsy, including pathways involving the SLD. Beyond the well-characterized orexin-vlPAG/LPT-SLD circuit, anatomical evidence shows that orexinergic neurons also project directly to the SLD, suggesting both direct and indirect pathways through which orexin may modulate SLD activity related to REM sleep and REM sleep atonia.

Although the authors briefly acknowledge the limitation that "the precise mechanisms by which orexin loss potentiates SLDGABA function require further investigation," it is strongly recommended that they incorporate a more detailed discussion of how the loss of orexin signaling—particularly the elimination of direct inputs to SLD neurons—may alter local GABAergic and glutamatergic dynamics and contribute to the observed sleep attacks and cataplexy.

6. In this study, the roles and mechanisms of SLD GABAergic neurons are based on artificial manipulations of cell activity. Investigating when SLD GABAergic neurons are naturally activated or inhibited under physiological or pathological conditions holds greater significance and is worthy of further exploration.

# Author comment: Author response to Decision Letter 2

**DOI:** 10.1371/journal.pbio.3003303.r003

5 Mar 2026

Attachment Submitted filename: Response to the reviewer-PLOSBiology-Mar-2026-FINAL.docx

# Peer review: Decision Letter 2

**DOI:** 10.1371/journal.pbio.3003303.r004

**Authors:** Taylor Hart, PhD

1 Apr 2026

Dear Dr Fraigne,

Thank you for your patience while we considered your revised manuscript "GABA neurons in the sublaterodorsal tegmental (SLD) nucleus suppress wakefulness in healthy and narcoleptic mice" for consideration as a Research Article at PLOS Biology. Your revised study has now been evaluated by the PLOS Biology editors, the Academic Editor, and one of the original reviewers.

In light of the reviews, which you will find at the end of this email, we are pleased to offer you the opportunity to address the remaining points from Reviewer 1 in a revision that we anticipate should not take you very long. We will then assess your revised manuscript and your response to the reviewers' comments with our Academic Editor aiming to avoid further rounds of peer-review, although we might need to consult with the reviewers, depending on the nature of the revisions.

In addition to these revisions, you may need to complete some formatting changes, which you will receive in a follow up email. A member of our team will be in touch with a set of requests shortly. If you do not receive a separate email within a few days, please assume that checks have been completed, and no additional changes are required.

We expect to receive your revised manuscript within 1 month. Please email us (plosbiology@plos.org) if you have any questions or concerns, or would like to request an extension.

At this stage, your manuscript remains formally under active consideration at our journal; please notify us by email if you do not intend to submit a revision so that we withdraw the manuscript.

**IMPORTANT - SUBMITTING YOUR REVISION**

Your revisions should address the specific points made by each reviewer. Please submit the following files along with your revised manuscript:

1. A 'Response to Reviewers' file - this should detail your responses to the editorial requests, present a point-by-point response to all of the reviewers' comments, and indicate the changes made to the manuscript.

*NOTE: In your point-by-point response to the reviewers, please provide the full context of each review. Do not selectively quote paragraphs or sentences to reply to. The entire set of reviewer comments should be present in full and each specific point should be responded to individually.

You should also cite any additional relevant literature that has been published since the original submission and mention any additional citations in your response.

2. In addition to a clean copy of the manuscript, please also upload a 'track-changes' version of your manuscript that specifies the edits made. This should be uploaded as a "Revised Article with Changes Highlighted " file type.

*Resubmission Checklist*

When you are ready to resubmit your revised manuscript, please refer to this resubmission checklist: https://plos.io/Biology_Checklist

To submit a revised version of your manuscript, please go to https://www.editorialmanager.com/pbiology/ and log in as an Author. Click the link labelled 'Submissions Needing Revision' where you will find your submission record.

Please make sure to read the following important policies and guidelines while preparing your revision:

*Published Peer Review*

Please note while forming your response, if your article is accepted, you may have the opportunity to make the peer review history publicly available. The record will include editor decision letters (with reviews) and your responses to reviewer comments. If eligible, we will contact you to opt in or out. Please see here for more details:

https://blogs.plos.org/plos/2019/05/plos-journals-now-open-for-published-peer-review/

*PLOS Data Policy*

Please note that as a condition of publication PLOS' data policy (http://journals.plos.org/plosbiology/s/data-availability) requires that you make available all data used to draw the conclusions arrived at in your manuscript. If you have not already done so, you must include any data used in your manuscript either in appropriate repositories, within the body of the manuscript, or as supporting information (N.B. this includes any numerical values that were used to generate graphs, histograms etc.). For an example see here: http://www.plosbiology.org/article/info%3Adoi%2F10.1371%2Fjournal.pbio.1001908#s5

*Blot and Gel Data Policy*

We require the original, uncropped and minimally adjusted images supporting all blot and gel results reported in an article's figures or Supporting Information files. We will require these files before a manuscript can be accepted so please prepare them now, if you have not already uploaded them. Please carefully read our guidelines for how to prepare and upload this data: https://journals.plos.org/plosbiology/s/figures#loc-blot-and-gel-reporting-requirements

*Protocols deposition*

To enhance the reproducibility of your results, we recommend that if applicable you deposit your laboratory protocols in protocols.io, where a protocol can be assigned its own identifier (DOI) such that it can be cited independently in the future. Additionally, PLOS ONE offers an option for publishing peer-reviewed Lab Protocol articles, which describe protocols hosted on protocols.io. Read more information on sharing protocols at https://plos.org/protocols?utm_medium=editorial-email&utm_source=authorletters&utm_campaign=protocols

Thank you again for your submission to our journal. We hope that our editorial process has been constructive thus far, and we welcome your feedback at any time. Please don't hesitate to contact us if you have any questions or comments.

Sincerely,

Taylor

Taylor Hart, PhD,

Associate Editor

PLOS Biology

thart@plos.org

----------------------------------------------------------------

REVIEW:

Reviewer #1 [Akihiro Yamanaka]: This revised manuscript is improved in several respects. The anatomical clarification, revised terminology, and summary schematic make the study easier to follow. The finding that SLD GABAergic neurons suppress wakefulness and can precipitate sleep attacks in orexin-deficient mice is interesting and potentially important. However, several concerns remain.

1. Closed-loop performance still requires more transparent reporting.

The manuscript states that the success rate of closed-loop illumination was 99.16%. It is unclear whether this value represents the overall accuracy across all state-dependent closed-loop conditions or only the best-performing condition. Please report the accuracy separately for each vigilance state. In addition, please provide the latency from state detection to light delivery for each condition.

2. The mechanistic discussion of SLD GABA neurons remains too definitive.

The prior literature does not yet establish a single physiological role for SLD GABA neurons. Rather, existing works suggest functional heterogeneity, with possible contributions to REM-state gating, wakefulness, and other aspects of pontine network activity. The current findings are interesting, but the Discussion should better acknowledge this complexity and avoid overly definitive statements.

3. The discussion of orexin signaling should be corrected and clarified.

The manuscript states that local orexin infusion into the SLD enhances local GABAergic signaling and promotes wakefulness, but the cited study appears to have examined infusion into the pontine reticular formation rather than the SLD itself. This should be corrected. More broadly, the Discussion should more clearly distinguish among direct effects of orexin within the SLD, indirect circuit effects, and possible downstream postsynaptic interactions.

4. The discrepancy between the orexin literature and the present optogenetic results should be addressed more directly.

In the current model, orexin is thought to promote wakefulness, whereas optogenetic activation of SLD GABA neurons suppresses wakefulness and triggers sleep attacks in orexin-deficient mice. This suggests that the circuitry is more complex than the current Discussion implies, possibly because of regional differences, cell-type heterogeneity, or differences between local neuromodulation and synchronous optogenetic activation. This point should be discussed explicitly.

5. The "final common pathway" interpretation remains overstated.

Although the silencing experiments are provocative, the current data do not yet establish that SLD GABA neurons constitute a final common pathway for both sleep attacks and cataplexy. The relevant downstream targets and neuronal subtypes remain unresolved, and the conclusions should therefore be tempered.

Overall, the study remains interesting, but these interpretive issues should be addressed more carefully.

# Author comment: Author response to Decision Letter 3

**DOI:** 10.1371/journal.pbio.3003303.r005

28 Apr 2026

Attachment Submitted filename: PLOSbio response to reviewer-revision2-FINAL.docx

# Editor report: Decision Letter 3

**DOI:** 10.1371/journal.pbio.3003303.r006

**Authors:** Taylor Hart, PhD

13 May 2026

Dear Dr Fraigne,

Thank you for your patience while we considered your revised manuscript "GABA neurons in the sublaterodorsal tegmental (SLD) nucleus suppress wakefulness in healthy and narcoleptic mice" for publication as a Research Article at PLOS Biology. This revised version of your manuscript has been evaluated by the PLOS Biology editors and the Academic Editor.

Based on our Academic Editor's assessment of your revision, we are likely to accept this manuscript for publication, provided that you address the following data and other policy-related requests.

**********************

IMPORTANT: Please ensure that your next revision addresses all of the following points:

**Title:

We would like to remove the '(SLD)' abbreviation from the title, in accordance with our stylistic preferences. Is this acceptable to you?

**Financial disclosure statement:

Please confirm that all relevant grant numbers have been included in the Financial disclosure statement in the manuscript details. Please also add links to the funding agencies to the statement.

**Ethics:

Please create a new, first sub-heading of the Materials and Methods section titled "Ethics statement". Please move all relevant ethical approval information under this sub-heading, including the protocol/permit/project license.

**Data:

You may be aware of the PLOS Data Policy, which requires that all data be made available without restriction: http://journals.plos.org/plosbiology/s/data-availability. For more information, please also see this editorial: http://dx.doi.org/10.1371/journal.pbio.1001797

Please supply the numerical values either in a supplementary excel file or as a permanent DOI’d deposition for the following figures panels, and ensure that these are accessible now:

1EIKNOPQ

2CDEIJKL (right subpanels)

4EFGJKLMNO (right subpanels)

5BCFK

S1GHIJKL (right subpanels)

S2ADGJMNOPQRSTUVWXY (right subpanels)

S3FGHIJK (right subpanels)

S4ABCDEFOPQRSTU (right subpanels)

S6IKLMNOP (right subpanels)

NOTE: the numerical data provided should include all replicates AND the way in which the plotted mean and errors were derived (it should not present only the mean/average values).

Please cite the location of the data clearly in all relevant main and supplementary Figure legends, e.g. “The data underlying this Figure can be found in S1 Data” or “The data underlying this Figure can be found in https://doi.org/10.5281/zenodo.XXXXX”

**Supplement format:

Please move the Materials and Methods into the main manuscript document.

Please also move the supplementary figure legends into the main text, and upload each supplementary figure file individually, as "Supporting Information".

Please also integrate the supplementary references into the main reference list, and remove any redundancies.

Supplementary files (e.g., excel). Please ensure that all data files are uploaded as 'Supporting Information' and are invariably referred to (in the manuscript, figure legends, and the Description field when uploading your files) using the following format verbatim: S1 Data, S2 Data, etc. Multiple panels of a single or even several figures can be included as multiple sheets in one excel file that is saved using exactly the following convention: S1_Data.xlsx (using an underscore).

**Code availability:

Per journal policy, if you have generated any custom code or scripts during the course of this investigation, we require that you make it available without restrictions. Please ensure that the code is sufficiently well documented and reusable, and that your Data Statement in the Editorial Manager submission system accurately describes where your code can be found. Please also ensure that you choose a license for your code and include a Readme file.

Please note that we cannot accept sole deposition of code in GitHub, as this could be changed after publication. However, you can archive this version of your publicly available GitHub code to Zenodo. Once you do this, it will generate a DOI number, which you will need to provide in the Data Accessibility Statement (you are welcome to also provide the GitHub access information). See the process for doing this here: https://docs.github.com/en/repositories/archiving-a-github-repository/referencing-and-citing-content

**********************

As you address these items, please take this last chance to review your reference list to ensure that it is complete and correct. If you have cited papers that have been retracted, please include the rationale for doing so in the manuscript text, or remove these references and replace them with relevant current references. Any changes to the reference list should be mentioned in the cover letter that accompanies your revised manuscript.

In addition to these revisions, you may need to complete some formatting changes, which you will receive in a follow up email. A member of our team will be in touch with a set of requests shortly. If you do not receive a separate email within a few days, please assume that checks have been completed, and no additional changes are required.

We expect to receive your revised manuscript within two weeks.

To submit your revision, please go to https://www.editorialmanager.com/pbiology/ and log in as an Author. Click the link labelled 'Submissions Needing Revision' to find your submission record. Your revised submission must include the following:

- a cover letter that should detail your responses to any editorial requests, if applicable, and whether changes have been made to the reference list

- a Response to Reviewers file that provides a detailed response to the reviewers' comments (if applicable, if not applicable please do not delete your existing 'Response to Reviewers' file.)

- a track-changes file indicating any changes that you have made to the manuscript.

NOTE: If Supporting Information files are included with your article, note that these are not copyedited and will be published as they are submitted. Please ensure that these files are legible and of high quality (at least 300 dpi) in an easily accessible file format. For this reason, please be aware that any references listed in an SI file will not be indexed. For more information, see our Supporting Information guidelines:

https://journals.plos.org/plosbiology/s/supporting-information

*Published Peer Review History*

Please note that you may have the opportunity to make the peer review history publicly available. The record will include editor decision letters (with reviews) and your responses to reviewer comments. If eligible, we will contact you to opt in or out. Please see here for more details:

https://plos.org/published-peer-review-history/

*Press*

Should you, your institution's press office or the journal office choose to press release your paper, please ensure you have opted out of Early Article Posting on the submission form. We ask that you notify us as soon as possible if you or your institution is planning to press release the article.

*Protocols deposition*

To enhance the reproducibility of your results, we recommend that if applicable you deposit your laboratory protocols in protocols.io, where a protocol can be assigned its own identifier (DOI) such that it can be cited independently in the future. Additionally, PLOS ONE offers an option for publishing peer-reviewed Lab Protocol articles, which describe protocols hosted on protocols.io. Read more information on sharing protocols at https://plos.org/protocols?utm_medium=editorial-email&utm_source=authorletters&utm_campaign=protocols

Please do not hesitate to contact me should you have any questions.

Sincerely,

Taylor

Taylor Hart, PhD,

Associate Editor

thart@plos.org

PLOS Biology

# Author comment: Author response to Decision Letter 4

**DOI:** 10.1371/journal.pbio.3003303.r007

27 May 2026

Attachment Submitted filename: PLOSbio response to reviewer-revision3-FINAL.docx

# Editor report: Decision Letter 4

**DOI:** 10.1371/journal.pbio.3003303.r008

**Authors:** Taylor Hart, PhD

10 Jun 2026

Dear Dr Fraigne,

Thank you for the submission of your revised Research Article "GABA neurons in the sublaterodorsal tegmental nucleus suppress wakefulness in healthy and narcoleptic mice" for publication in PLOS Biology. On behalf of my colleagues and the Academic Editor, Jozsef Csicsvari, I am pleased to say that we can in principle accept your manuscript for publication, provided you address any remaining formatting and reporting issues. These will be detailed in an email you should receive within 2-3 business days from our colleagues in the journal operations team; no action is required from you until then. Please note that we will not be able to formally accept your manuscript and schedule it for publication until you have completed any requested changes.

Please take a minute to log into Editorial Manager at http://www.editorialmanager.com/pbiology/, click the "Update My Information" link at the top of the page, and update your user information to ensure an efficient production process.

PRESS

We frequently collaborate with press offices. If your institution or institutions have a press office, please notify them about your upcoming paper at this point, to enable them to help maximise its impact. If the press office is planning to promote your findings, we would be grateful if they could coordinate with biologypress@plos.org. If you have previously opted in to the early version process, we ask that you notify us immediately of any press plans so that we may opt out on your behalf.

We also ask that you take this opportunity to read our Embargo Policy regarding the discussion, promotion and media coverage of work that is yet to be published by PLOS. As your manuscript is not yet published, it is bound by the conditions of our Embargo Policy. Please be aware that this policy is in place both to ensure that any press coverage of your article is fully substantiated and to provide a direct link between such coverage and the published work. For full details of our Embargo Policy, please visit http://www.plos.org/about/media-inquiries/embargo-policy/.

Thank you again for choosing PLOS Biology for publication and supporting Open Access publishing. We look forward to publishing your study.

Sincerely,

Taylor

Taylor Hart, PhD,

Associate Editor

PLOS Biology

thart@plos.org
